# Effectiveness of continuing professional development training of welfare professionals on outcomes for children and young people: A systematic review

**DOI:** 10.1002/cl2.1060

**Published:** 2019-11-07

**Authors:** Trine Filges, Carole Torgerson, Louise Gascoine, Jens Dietrichson, Chantal Nielsen, Bjørn A. Viinholt

**Affiliations:** ^1^ VIVE‐Campbell Copenhagen Denmark; ^2^ School of Education Durham University Durham UK

## 1 PLAIN LANGUAGE SUMMARY

### 1.1 Little evidence of the effectiveness of continuing professional development (CPD)

CPD aims to improve outcomes for the children and young people with whom educational and welfare professionals work. There is no clear evidence that CPD in education improves student academic outcomes.

### 1.2 What is this review about?

CPD is delivered in a variety of settings by different kinds of “trainers” or educators for differing lengths of time and differing intensity. There are many methods of delivery such as coaching sessions, feedback based on observations or videotapes of classroom practice, and feedback and reflection workshops.

This review looked at the effects of CPD approaches for education and welfare practitioners (preschool teachers, pedagogues, school teachers, social workers, psychologists, police officers) on educational, social, crime and justice outcomes for children and young people; and—as secondary outcomes—any effects on the professional practice of practitioners in these fields. For the purposes of this review, the CPD must involve the development of core professional skills.

**What is the aim of this review?**

This Campbell systematic review (SR) examines the effects of CPD approaches for education and welfare practitioners on: educational and social outcomes for children and young people; and outcomes for practitioners. The review summarises evidence from 51 moderate‐quality studies, including 48 randomised controlled trials (RCTs) and three quasiexperiments.

### 1.3 What studies are included?

This review includes studies that evaluate the effects of CPD on children's or young people's and professionals' outcomes. Fifty‐one studies were identified, all related to education. No eligible studies were identified for social welfare or crime and justice.

The 51 education studies were grouped into three subtopic areas: 12 studies (reporting 10 trials) considered CPD in social and emotional development interventions (in daycare, kindergarten, preschool and school settings); 38 studies (reporting 33 trials) dealt with CPD in language and literacy development interventions; one study looked at CPD in stress reduction. Most (48) studies used experimental designs with random assignment.

Only 26 of the 51 studies were included in the meta‐analyses. The reduction was caused by studies reporting on the same trial (five studies), insufficient reporting of outcomes to calculate an effect size (four studies) and studies being rated to have too high risk of bias. In total 16 studies were assessed not to be of sufficient methodological quality to be included in the meta‐analyses.

The studies spanned the period 1999–2018. Thirty‐three trials were undertaken in the United States, two in the UK and one in each of the following countries: Denmark, Ireland, the Netherlands, New Zealand, Portugal, Australia, Chile and Germany.

### 1.4 What are the main findings of this review?

Social and emotional development interventions (nine studies)

A very small body of evidence for social and emotional development interventions (in daycare, kindergarten, preschool and school settings) finds no effect of CPD on student academic outcomes (four studies). Results from only two individual studies could be combined in a single meta‐analysis of other student outcomes (i.e., nonacademic) and teacher outcomes, precluding any conclusions concerning effectiveness or ineffectiveness of this type of CPD on these outcomes.

Language and literacy development interventions (17 studies)

A moderate body of evidence for language and literacy development interventions finds no effect for CPD on student academic outcomes (13 studies). The results from only three individual studies could be combined in a single meta‐analysis of teacher outcomes, thus precluding any conclusions concerning effectiveness or ineffectiveness of this type of CPD on teacher outcomes.

Stress reduction (one study)

It is not possible to draw conclusions from the one study placed in the subtopic of stress reduction.

### 1.5 What do the findings of this review mean?

There is insufficient evidence for conclusions to be drawn, with the exception of language and literacy development interventions. For this type of CPD, there seems to be no effect on student academic outcomes.

The dominance of the United States as the main country in which the types of CPD interventions covered by this review have been evaluated clearly limits the generalisability of the findings. Moreover, the limited number of studies means that it was not possible to conduct an analysis of specific CPD‐approaches across cultures, professions/service‐deliverer types, organisations and service‐receiver types.

Agencies should consider conducting a large RCT (or a series of large RCTs) evaluating the effectiveness of a CPD intervention in countries outside the United States.

### 1.6 How up‐to‐date is this review?

The review authors searched for studies up to December 2018.

## 2 EXECUTIVE SUMMARY/ABSTRACT

### 2.1 Background

The quality of the CPD of education and welfare professionals working with children and young people is of key importance to policy makers and practitioners in these fields. In order to inform education and welfare professions about the nature and effectiveness of a diversity of approaches to CPD, a SR of the international literature was undertaken.

In western societies, there is an increasing acknowledgement of the value of working with evidence‐informed approaches and methods. Therefore, the results of this SR are of utmost relevance.

The review aimed to systematically search for, locate, quality appraise and synthesise all the available effectiveness studies which evaluated relevant interventions using rigorous designs.

### 2.2 Objectives

The research questions were:

- What are the effects of CPD approaches for education and welfare practitioners (preschool teachers, pedagogues, school teachers, social workers, psychologists, police officers) on: educational, social, crime and justice outcomes for children and young people; and on outcomes for practitioners
- What empirical evidence is there on the external validity of specific CPD‐approaches across cultures, across professions/service‐deliverer types, across organisations and across service‐receiver types

### 2.3 Search methods

The search was concluded in December 2018. Relevant studies were identified through electronic searches of bibliographic databases, specific targeted relevant online repositories and internet search engines. We searched to identify both published and unpublished literature. Reference lists of included studies and reviews were also searched.

### 2.4 Design and methods; selection criteria

The design of the review is a full SR. Studies that can adequately address the primary research question (which is an effectiveness question) are high‐quality evaluations of CPD interventions to improve educational and social outcomes for children and young people and professional practice outcomes for practitioners using experimental designs: RCTs, quasirandomised trials, and studies of quasiexperimental designs (QEDs).

Studies that utilised other approaches were not included in the review due to the absence of adequate control group conditions.

Studies were only included if they included at least one valid and reliable outcome (a standardised, validated test) that had been standardised on a different population.

### 2.5 Data collection and analysis

The electronic searches identified 5,146 potentially relevant studies for screening of titles, abstracts and full papers using the inclusion/exclusion criteria. After three stages of independent double screening, 51 studies were included in the review: all were in the area of education. The studies could be grouped into three subtopic areas according to the focus of the professional development (PD) being investigated, although most (50) were in two of these subtopic areas: 38 studies dealt with PD in language and literacy development interventions and outcomes; 12 studies investigated social and emotional development interventions and outcomes. One study looked at PD interventions related to stress reduction.

In the social and emotional development subtopic area two trials were reported in two papers each, thus the number of trials was 10. In language and literacy, the number of trials was 33; two trials were reported in two papers each and one trial was reported in four papers.

Thirty‐four trials were conducted in the United States, with only one study undertaken in each of the following countries: Australia, Chile, Denmark, Germany, Ireland, the Netherlands, New Zealand and Portugal; and two trials were undertaken in the UK.

The professional participants in the evaluations of PD interventions were exclusively preschool teachers (pedagogues) and teachers. The other participants were exclusively children and young people attending preschool (including “day care”), kindergarten (nursery) or school settings.

All of the included studies met a minimum threshold for quality due to the inclusion criterion for this review. The meta‐analyses focused on the social and emotional development subtopic area and the language and literacy subtopic area.

All except three (in the language and literacy development area) of the studies in the review were RCTs. Overall, the included studies varied on risk of bias judgements and no single study could be characterised as a robust RCT with low risk of bias on all assessed risk of bias items. In total, 17 studies, the one evaluating stress reduction and all the remaining in the language and literacy area, were given a score of 5 on at least one of the risk of bias items, corresponding to a risk of bias so high that the findings should not be considered in the meta‐analysis.

Random effects models were used to pool data across the studies. We used the standardised mean difference (SMD); Hedges' *g* was used for estimating the SMD and we applied the small *N* correction. Pooled estimates were weighted with inverse variance methods, and 95% confidence intervals (CIs) were used. Funnel plots were used to assess the possibility of publication bias. Sensitivity analysis was used to evaluate whether the pooled effect sizes were robust to cluster correction and across study design and components of methodological quality.

### 2.6 Results

We used homogeneity of professional and student outcomes in the two subtopic areas as the basis of the meta‐analyses. Control conditions were very similar and tended to comprise business as usual PD.

All, except three studies in the language and literacy development area and one in the social and emotional development area, reported either student or teacher outcomes that enabled the calculation of a SMD and standard error approximately by the end of the intervention. Twenty‐six studies were left for meta‐analysis; nine in the social and emotional development area and 17 in the language and literacy area.

#### 2.6.1 Social and emotional development

The sample sizes reported in the studies used in the meta‐analyses in the social and emotional development topic area varied between 99 students to 1,685 students with an average of 914 students; 22 classes to 224 classes with an average of 95 and nine schools to 58 schools with an average of 26 schools.

Four studies could be combined in a meta‐analysis of student academic outcomes. There seems to be no effect on student academic outcomes. The weighted average SMD was 0.05 (95% CI [−0.07, 0.16]) and not statistically significant. There was evidence of some heterogeneity between the studies.

At most the results from two individual studies could be combined in a single meta‐analysis of other student outcomes and teacher outcomes. The weighted average SMD of student social competences was 0.13 (95% CI [0.03, 0.24]) and 0.22 (95% CI [0.08, 0.37]) for student's socioemotional skills.

Three studies reported outcomes on various other student measures that were too different to be combined.

Teacher outcomes were reported on the three subscales of CLASS (Positive climate, Negative climate and Behavioural management). The weighted average SMD of Positive climate is 0.61 (95% CI [0.08, 1.14]); for Negative climate it is 0.18 (95% CI [−0.73, 1.08]) and for Behaviour management it is 0.30 (95% CI [−0.14, 0.73]).

#### 2.6.2 Language and literacy

The sample sizes reported in the studies used in the meta‐analyses in the language and literacy area varied between 164 students to 4,078 students with an average of 1,632 students; 24 classes to 324 classes with an average of 113; and four schools to 224 schools with an average of 58 schools.

Thirteen studies reported results on student academic outcomes in the language and literacy development topic area. There seems to be no effect on student academic outcomes. The weighted average SMD was 0.04 (95% CI [−0.01, 0.10]). The result was somewhat sensitive due to the removal of studies with scores of 4 on the blinding component; the weighted average effect became larger and statistically significant when studies with blinding scores of 4 where removed. Note, however, that only four studies contributed to the average. There was no evidence of heterogeneity. No other student outcomes were reported.

At most the results from three individual studies could be combined in a single meta‐analysis of teacher outcomes.

There seem to be a positive effect on teacher outcomes measured by Early Language and Literacy Classroom Observation (ELLCO), the weighted average SMD was 0.45 (95% CI [0.16, 0.74]) and there was a small amount of heterogeneity between the studies.

There also seems to be a positive effect on teacher outcomes measured by three summary CLASS measures: Emotional support, Instructional support and Classroom organisation. The weighted average SMD of Emotional support was 0.30 (95% CI [0.11, 0.49]); for Classroom organisation it was 0.23 (95% CI [0.04, 0.43]) and for Instructional support it was 0.20 (95% CI [0.01, 0.39]). There was no evidence of heterogeneity between the studies. The weighted average of Instructional support lost statistical significance in the sensitivity analysis of cluster correction, otherwise none of the results changed.

One study further reported results from two ELLCO subscales and one study reported results on mathematics teaching practices.

We did not find any adverse effects.

### 2.7 Authors' conclusions

A moderate body of experimental evidence exists in relation to the effect of PD in the topic area of education; similar evidence does not appear to exist in the topic areas of social welfare and crime and justice.

A small body of evidence exists in relation to the effect of PD in social and emotional development interventions on students and teachers. The majority of studies do not report on student outcomes while the teacher outcomes reported are, with few exceptions, too different to be combined.

A moderate number of experimental evaluations of PD in language and literacy have been undertaken, mainly in the United States. The number of studies to be used in the meta‐analysis was reduced from 38 to 17. The reduction was caused by studies reporting on the same trial (two studies), insufficient reporting of outcomes to calculate an effect size (three studies) and studies being rated to have too high risk of bias. In total 16 studies were judged to have a very high risk of bias (5 on the scale) and, in accordance with the protocol, we excluded these from the meta‐analysis on the basis that they would be more likely to mislead than inform.

In short, the result of the review is that there is currently insufficient evidence for conclusions to be drawn except for students in the language and literacy subtopic area, where there seem to be no effect on student academic outcomes; the weighted average effect is very small and not statistically significant.

Otherwise, the small number of available studies reporting similar outcomes precludes any conclusions concerning effectiveness or ineffectiveness of PD. Moreover, the limited number of studies prevented an analysis of specific PD‐approaches across cultures, across professions/service‐deliverer types, across organisations and across service‐receiver types.

The vast majority of studies were undertaken in the United States. The dominance of the United States as the main country in which PD interventions meeting our criteria have been evaluated using rigorous methods and within our specific parameters clearly limits the generalisability of the findings. None of the studies, however, was considered to be of overall high quality in our risk of bias assessment and the process of excluding studies with too high risk of bias from the meta‐analysis applied in this review left us with only 17 of a total of 33 possible studies to synthesise in the language and literacy area.

This is a finding in its own right, entailing important information for stakeholders on the degree of confidence to place on the expected gains from PD in the language and literacy area.

Given the limited number of rigorous studies available from countries other than the United States, it would be natural to consider conducting a large RCT (or a series of large RCTs) evaluating the effectiveness of a PD intervention in the topic area of social and emotional development or language/literacy development in countries outside of the United States. The trial(s) should be designed, conducted and reported according to methodological criteria for rigour in respect of internal and external validity in order to achieve robust results.

## 3 BACKGROUND

### 3.1 The problem, condition or issue

The quality of the professional development of education and welfare professionals working with children and young people (e.g., preschool teachers or pedagogues, school teachers, social workers, psychologists, police officers, etc.) is of key importance to policy makers and practitioners in these fields. The general wellbeing of a country's citizens and the provision of better opportunities in terms of educational and social welfare outcomes (e.g., participation in higher education and reduction of anti‐social behaviour) have been linked to the quality of PD available to the welfare professionals. Conversely, a potential barrier to achieving these education and welfare aspirations is the variable quality of the professional training delivered to the educational and/or welfare practitioners, due to the challenges of designing and implementing high quality PD and this could mean that the education and training of these groups of professionals may, sometimes, be less than optimal.

In order to inform education and welfare professions—policy makers and practitioners—about the nature and effectiveness of a diversity of approaches to CPD, a SR of the international, high quality causal literature was undertaken.

Following the conceptualisation proposed by Buysse and Hollingsworth (2009), one can think of professional development programmes in terms of *who* (providers and learners), *what* (the content) and *how* (the organisation and facilitation of the learning experiences). In relation to this present review, the learners we consider (the *who*), are recipients of CPD, that is, professionals, who have already completed their initial training as professionals and are thus fully qualified and in employment. CPD can be thought of as a specific type of PD. For the purposes of this review we focus on CPD and use the terms CPD and PD interchangeably.

As will be clear in the following, we only found studies that fulfilled our inclusion criteria in the field of education. Hence, the examples provided in the literature contextualisation section focuses on this topic area.

In terms of content (the *what*), many PD programmes that would be considered relevant for this review, will focus broadly on training to improve adult‐child interactions and caregiving since this is the strongest predictor of children's skill development (NICHD Early Child Care Research Network, 2002). Moreover, since teacher‐child interactions mediate the effects of organised curricula on children's skills development, such interactions are central to PD programmes aiming to improve child outcomes in a broader sense (Pianta, La Paro & Hamre, 2006). Hence, PD programmes of relevance for this review will include content where the aim is to:

- Improve professionals' ability to provide children with emotional support
- Increase professionals' awareness of the importance of meeting students with high expectations
- Create more positive teacher/child interactions at the individual level
- Use positive behaviour‐management strategies at the classroom level

PD contect that focuses on developing teachers' knowledge and understanding in more substantive fields such as language and literacy development, numeracy skills development, and so forth, are also relevant for this review.

Beyond the *who* and the *what*, it is relevant to ask *how*. CPD is delivered in many different ways. Buysse, Winton, and Rous (2009), Darling‐Hammond, Hyler, and Gardner (2017), Egert et al. (2018) and Pianta et al. (2006) have argued that effective programmes tend to focus on specific content, for instance a new curriculum or content based on a quality rating scale. This could take the form of example lesson plans, unit plans, sample student work, observations of peer teachers and video or written cases of teaching, thereby providing teachers with a clear vision of best or desired practices. Some highlight the benefits of collaboration with and feedback from fellow teachers since this mode of provision can facilitate reflection and help learning. Collborating with colleagues can moreover provide opportunities for changing teacher practices at the organisational level (Buysse & Hollingsworth, 2009; Darling‐Hammond et al., 2017).

CPD is provided by different kinds of “trainers” or educators and implemented in a variety of settings for differing lengths of time and differing intensity. According to Buysse and Hollingsworth (2009), Darling‐Hammond et al. (2017) and Pianta et al. (2006), programmes should be both intensive and not too short, in order to facilitate reflection, while at the same time retaining focus.

However, there may easily be a gap between the theoretically expected effectiveness of particular design features and practical reality. Kennedy (2016) characterised PD programmes in terms of their theories of action—defined in terms of the content teachers should learn—and how programmes facilitate teachers' *enactment* of the content. According to Kennedy's typology of enactment facilitation, PD programmes range from being highly prescriptive to simply providing a body of knowledge that teachers may choose to react to or not. Highly prescriptive programmes clearly limit teacher discretion and there may also be a tension between prescription and motivation. The effects of any PD programme will depend on teachers' motivation to learn and to change their practice, mandatory assignment of teachers to programmes may not have much effect on learning (Kennedy, 2016). Clearly, contextual aspects such as the workplace environment and organisational support may also moderate the effects of any type of PD (Egert et al., 2018; Kennedy, 2016). Individual teachers or schools forced into a PD programme may not provide enough personal engagement or organisational support, respectively, to change practices.

Hence, on top of the already‐complex task of teaching and caring for children and young people, having to undertake CPD may present professionals with an additional burden. The perception of a PD programme will depend entirely on who is to receive and deliver it; whether the content is relevant and useful; and whether the mode of delivery is suitable for the individual and organisational context. In the process of conducting this review, it has become clear that the variation in types of PD provided to professionals working with children and young people is indeed very large.

#### 3.1.1 Aim of this review

The review aimed to systematically search for, locate, quality appraise and synthesise all the available effectiveness studies which evaluated relevant interventions using rigorous designs. By “rigorous designs” we refer to those research designs that can establish a causal link between CPD interventions and outcomes for professionals themselves, children and young people. Therefore, we included: SR and meta‐analytic designs, “true” experiments (RCTs), quasiexperiments (with baseline equivalence as demonstrated by pretests in the outcomes of interest, but excluding studies using an instrumental variable approach, see Appendix A), including studies using regression discontinuity (RD) design.

We searched substantively for studies in the topic areas of education, social welfare and crime and justice. An initial scoping search on one database was undertaken, using the following search strategy:

TI (teacher OR social worker OR police OR psychologist) AND TI (professional development OR continuing professional development OR CPD OR in service training OR professional learning OR teacher learning OR training) AND AB (experiment* OR quasi experiment* OR QED OR control OR allocat* OR randomi#ed controlled trial OR RCT OR regression discontinuity OR RDD)

This scoping search produced 470 potentially relevant “hits”, which, after screening using preliminary inclusion criteria, indicated that a range of potentially relevant studies, mainly in the topic area of education, but also in other areas of social welfare and policing were available to be systematically assembled. We were also aware of a recently published meta‐analysis in the specific area of professional development in professionals working with children's early language and literacy development (Markussen‐Brown et al., 2017). This meta‐analysis formed part of the basis of our electronic and citation searching in the topic area of education. Note that our search covered the entire field of education and was not limited to studies on language and literacy development.

The review was completed using SR design and methods that are open to scrutiny (Torgerson, 2003), as this minimises bias and increases confidence in the results.

### 3.2 Description of the condition

Education and welfare professionals are employees working directly or indirectly with and for children and young people with the explicit purpose of enhancing their cognitive and noncognitive development. This includes, but is not limited to, education and welfare employees working towards these goals in settings such as nurseries, day care and other child care institutions, preschools, and schools at different levels. Education and welfare professionals can be either publicly or privately employed, they receive salary for their work, which may be full‐time or part‐time. Education^1^ and welfare professionals have completed ordinary (basic) training at a higher education institute relevant for their professional degree. This degree can be at varying International Standard Classification of Education (ISCED)‐levels (e.g., diploma, postgraduate certificate, B.A., M.Sc., Ph.D.). Education and welfare professionals are recipients of the PD activities and interventions that are being evaluated.

Examples of education and welfare professionals include teachers, teaching assistants (TAs), preschool teachers (pedagogues), care providers, social workers, paraprofessionals, psychologists, police officers, family support providers, disability specialists, inclusion specialists. The roles of education and welfare professionals include planning, developing, delivering and evaluating learning and development opportunities for children and young people.

### 3.3 The intervention

For the purpose of this review, we have adopted the following definitions, inspired by Buysse et al. (2010):

#### 3.3.1 Continuing professional development

- CPD encompasses facilitated learning opportunities for education and welfare professionals that have completed their ordinary (basic) training at an (higher) education institute relevant for their professional degree. This (previous) degree can be at varying ISCED‐levels (e.g., diploma, B.A., M.Sc., Ph.D.)
- CPD includes all types of facilitated learning opportunities. Some types of CPD will be shorter term, informal, situated in practice and will not lead to credits, diplomas or degrees. Other types of CPD will be longer term, involve formal coursework and take place at teachers' colleges or universities, and will lead to credits, diplomas or degrees
- The aim of CPD should be to enhance the professionals' knowledge and skills in ways that are relevant for application in practice, that is, to serve the ultimate beneficiaries of the intervention, that is, the children and young people with / for whom the education and welfare professionals work
- CPD can be delivered by public or private professional development and professional training entities

CPD can be delivered in many more or less formal ways, including coaching, mentoring, consultations and established communities or teams of practice. In such cases, the CPD must have explicitly formulated content and goals. Note that (informal) allocation of a mentor for the purpose of general collegial support is not included in this definition of CPD.

### 3.4 How the intervention might work

CPD enhances the professionals' knowledge and skills in ways that are relevant to better serve the ultimate beneficiaries of the intervention, that is, the children and young people with / for whom the education and welfare professionals work.

### 3.5 Why it is important to do the review

In order to inform education and welfare professions—policy makers and practitioners—about the nature and effectiveness of a diversity of approaches to CPD it is important to systematically search for, locate, quality appraise and synthesise all the available effectiveness studies.

#### 3.5.1 Literature contextualisation

Two previous “tertiary” reviewsI—or reviews of reviews—in the field of professional development of educators have been undertaken: Dunst et al. (2015)^2^ and Cordingley et al. (2015).

In their meta‐synthesis of 15 reviews, Dunst et al. (2015) looked at the features of PD (in terms of delivery, pedagogy, etc.) which were associated with positive teacher and student outcomes in the included SRs and concluded that a range of key PD characteristics led to positive outcomes. However, most of the reviews in this meta‐synthesis did not meet our criteria for inclusion on the basis of key items reported in the article. This was due to a variety of factors: a review not using SR or meta‐analytic design, or not focusing on PD as we defined it, for example focusing on induction for beginning teachers. Where a SR included in this meta‐synthesis was relevant to our review, this was subsequently citation searched for relevant empirical studies (Blank & de las Alas, 2009; Zaslow, Tout, Halle, Whittaker, & Lavelle, 2010).

In their “umbrella” review, Cordingley et al. (2015) included nine reviews from the international literature looking at effective professional development relating the findings from the reviews to standards of rigour. One review, not identified through the electronic searching, met our inclusion criteria (Timperley, Wilson, Barrar, & Fung, 2007) was judged to be consistently robust in all aspects of methodology and this was citation searched for our SR.

There are several SRs of professional development in the education area; not consistently robust in all aspects of methodology to be citation searched for our SR, as for example Darling‐Hammond et al. (2017) and Kennedy (2016). No meta‐analyses have been performed in any of these. The review by Darling‐Hammond et al. (2017) provides a narrative analysis of 35 studies, restricted to studies findings positive effects of professional development; and the review by Kennedy (2016) provides a visual analysis of the impact of programme (sorted according to two central aspects of theories of action) and study design. The review we have done differs in a substantial way from these existing reviews; we followed standard procedures for conducting SRs using meta‐analysis techniques. Meta‐analyses of the overall effects were conducted.

Four SRs with meta‐analyses were found in Basma and Savage (2018), Egert et al. (2018), Kraft et al. (2018) and Markussen‐Brown et al. (2017). They were all citation searched for relevant empirical studies.

The review by Basma and Savage (2018) included 17 studies of teacher professional development in elementary school that measured the impact on students' reading measures (excluding narrative and writing outcomes). Studies that were correlational or did not include a control group were excluded. The use of nonstandardised outcome measures was not an exclusion reason. The date of search is not reported but the latest included study is published in 2015. A large number (65 effect size are reported in Table 3) of literacy effect sizes were extracted from the studies, including effect sizes from multiple treaments and multiple measurement times in one study. However only one effect size from each study was used in the meta‐analysis. It is not reported how this one effect size per study was calculated or chosen from studies reporting multiple reading measures.

The review by Egert et al. (2018) performed searches up to 2011 and included 36 studies (reporting on 42 different treatments) of professional development programmes for early childhood teachers (preschool to kindergarten) on quality ratings of childcare (teacher outcomes) and child outcomes. Studies only providing self‐evaluation of quality ratings were excluded. Many of the included studies did not have control groups, that is, used a one group before‐after design. All types of quality ratings, measured by standardised instruments such as CLASS or ELLCO as well as nonstandardised instruments (the authors describe them as not internationally recognised instruments), were combined in one meta analysis and all child outcomes (academic as well as social behaviour, etc.) were combined in one meta analysis. A large number of effect sizes at posttest were extracted, 289 effect sizes on teacher outcomes and 68 effect sizes for child outcomes. One “aggregated” effect size of teacher outcomes respectively child outcomes from each study (treatment) was used in the two meta‐analyses, the procedure of aggregation was not reported.

Markussen‐Brown et al. (2017) conducted a SR and meta‐analysis in the specific area of professional development in professionals working with children's early language and literacy development. Participants had to be in‐service educators or home‐based child‐care providers working with 3–6‐year‐old children United States or Canada. Searches were conducted between October 11, 2013 and March 13, 2014. Twenty‐five studies (containing 33 trials altogether) were included. The included studies had to be published in peer‐reviewed journals making the results susceptible to publication bias. Markussen‐Brown et al. (2017) conducted meta‐analyses to evaluate the effects of language‐ and literacy‐focused PD on the teacher outcomes process quality, structural quality and educator knowledge as primary outcomes; self‐reported measures were excluded. Furthermore, three child outcomes were analysed: receptive vocabulary, phonological awareness and alphabet knowledge.

Kraft et al. (2018) undertook a SR in the topic area of education PD and focused narrowly on one specific PD intervention for teachers: “teacher coaching” performing searches up to 2017. Participants had to be in‐service teachers working with students in early childhood to 12th grade in United States or “other developed countries”. Although the scope of our review was broader in terms of including research into the effectiveness of *any* PD aimed at education and social welfare professionals, Kraft et al's four inclusion criteria overlapped with our inclusion criteria and we also included studies of causal designs that evaluated coaching interventions for teachers. Sixty studies were included in the Kraft et al. (2018) review and meta‐analyses were conducted to evaluate the effects of teacher coaching programmes on teacher instruction and student achievement. All available measures of teacher instruction (although it should be rated by an outside observer) was used in a single meta‐analysis; 186 effect estimates from 43 studies were extracted. Likewise, a large number of measures, 113 effect estimates from 31 studies, was used in the meta‐analysis of student achievement. Robust variance estimation methods were used to account for the nonindependence of multiple effect sizes from the studies.

Although the scope of our review was broader in terms of including research into the effectiveness of *any* PD aimed at education and social welfare professionals as well as crime and justice, the inclusion criterias in these four reviews overlap with our inclusion criterias. Besides being up to date, a major difference between these four SRs and our SR iwa that our inclusion criteria were more specific for outcomes, and we undertook a systematic and transparent risk of bias assessment before including any study in a meta‐analysis, excluding studies with too high risk of bias from the meta‐analysis.

## 4 OBJECTIVES

The research questions were:

- What are the effects of CPD approaches for education and welfare practitioners on: educational and social outcomes for children and young people; and on outcomes for practitioners
- What empirical evidence is there on the external validity of specific PD‐approaches across cultures, across professions/service‐deliverer types, across organisations, across service‐receiver types, and so forth.

## 5 METHODS

The design of the review is a full SR; the design and methods of the review were informed by the Campbell Collaboration policy briefs (Campbell Collaboration, 2018); “Systematic reviews: CRD's guidance for undertaking reviews in health care” (University of York, Centre for Reviews and Dissemination, 2009); the “Cochrane Collaboration Handbook” (Higgins & Green, 2011); the *Handbook of Research Synthesis* (Cooper & Hedges, 1994) and *Systematic Reviews* (Torgerson, 2003). The design and methods for each stage of the SR were outlined in a protocol which was developed before searching for potentially relevant studies began and which outlined a priori the inclusion and exclusion criteria. The protocol was published as a note at The Danish National Centre for Social Research (SFI)^3^ (Torgerson et al., 2017) following approval from Trygfonden (one of the main funders for the review).

The reporting of each stage of the SR process was guided by the PRISMA (Preferred Reporting Items for Systematic Reviews and Meta‐Analyses) statement (Moher, Liberati, Tetzlaff, & Altman, 2009) to ensure transparency.

### 5.1 Criteria for considering studies for this review

#### 5.1.1 Types of studies

Studies that can adequately address the primary research question (which is an effectiveness question) are high‐quality evaluations of CPD interventions to improve educational and social outcomes for children and young people and professional practice outcomes for practitioners using experimental designs: RCTs, quasirandomised trials and quasiexperiments. We only included study designs that employ a treatment‐control or a treatment‐comparison group design. A control group is defined as a nontreatment condition, while a comparison group receives an alternative treatment. Studies using single group prepost comparisons were not included; in order to establish causality (i.e., to be able to state that a specific professional development intervention *causes* an improvement in the outcomes stated above), study designs which can adequately control for all other known and unknown variables that could affect outcome are required (Cook, Campbell, & Boston, 1979; Shadish, Cook, Campbell, & Boston, 2002).

1. Randomised and quasi‐RCTs (allocated at either the individual level or cluster level, for example, class/school/social worker/geographical area, etc.).
2. Quasiexperimental studies (including RD design, but excluding studies using an instrumental variable approach—see Appendix A for our rationale for excluding studies of these designs). We also only included QED studies that demonstrated baseline equivalence in the main outcomes of interest. A further requirement was that these studies were able to identify an intervention effect. Studies where, for example, the treatment was given to teachers in one school only and the comparison group was teachers at another school (or more schools for that matter) could not separate the treatment effect from the school effect.

This review focuses on research evidence from academic journals and other published research from the last 21 years (as this provides the most up‐to‐date evidence for policy makers, practitioners and funders on effective practices, strategies and interventions). In order to limit the possibility of publication bias, research from difficult‐to‐locate “grey” literature was searched for and included. Our approach to the search for “grey” literature is described in a separate section below.

Studies in which at least one of the groups received a CPD intervention compared to either standard practice (“business‐as‐usual”) or an alternative CPD intervention were included.

**Table:** 

Included	Excluded
Date: 1997 to present	Date: pre‐1997
Publication status: published or unpublished but in the public domain	
Nature of research: empirical research or review of empirical research	Nature of research: nonempirical research or review of nonempirical research
Study design: RCT; quasiexperiment (with baseline equivalence), including RDD	Study design: study using IV approach; nonexperimental study designs (i.e., studies without a control or comparison group)
Topic: education, social welfare, crime and justice	Topic: not education, social welfare, crime and justice

#### 5.1.2 Types of participants

**Table:** 

Included	Excluded
Participants: welfare professional (preschool teacher, “pedagogue”, school teacher, social worker, psychologist, police officer^a^)	Participants: not welfare professionals (e.g., volunteers) or welfare professionals in a school‐based role that does not require a professional degree (e.g., TAs)
Participants: target group (children and young people between the ages of 0 and 18 years)	Participants: aged 19 years and over (adults)

^a^There are established graduate entry routes into the police in the UK context

#### 5.1.3 Types of interventions

**Table:** 

Included	Excluded
Intervention: intervention in CPD in the three topic areas (education, social welfare, crime and justice). CPD includes, but is not restricted to: focused supervision; feedback; team work or other kinds of training/PD approaches; literacy and language teaching skills, problem solving teaching skills, socioemotional development skills and other CPD content	Intervention: does not have a CPD component; initial training intervention/PD (e.g., initial teacher training)
Outcomes: primary: educational, social welfare and crime and justice outcomes for children and young people; secondary: any intermediate outcomes on children and young people such as at‐risk behaviours; family outcomes; any outcomes for practitioners that are focused on improving any aspect of professional practice	Outcomes not related to education, social welfare and crime and justice. Practitioner outcomes not focused on improving professional practice, for example, higher job satisfaction
Studies were only included if they included at least one valid and reliable outcome that had been standardised on a different population *[and was “objective”, that is, not “experimenter‐designed” and not self‐reported]	*[“Experimenter designed” outcomes]
	*[Self‐reported outcomes]

#### 5.1.4 Types of outcome measures

##### 5.1.4.1 Primary outcomes

Educational, social welfare and crime and justice outcomes for children and young people.

##### 5.1.4.2 Secondary outcomes

Any intermediate outcomes on children and young people such as at‐risk behaviours; family outcomes; any outcomes for practitioners that are focused on improving any aspect of professional practice.

Outcomes not related to education, social welfare and crime and justice were excluded. Practitioner outcomes not focused on improving professional practice, for example, higher job satisfaction were excluded.

*Experimenter designed outcome measures* that have been designed by the author(s) have typically been developed for the specific study and have not been validated or standardised with another sample. Experimenter developed measures have been shown to have much higher effect sizes in a very large sample of educational intervention studies (Cheung & Slavin, 2016). In some cases, the instruments have been pilot‐tested, but this is not adequate in terms of being able to have full confidence in the quality and validity of the outcome measure. In other cases, the authors have combined existing instruments with experimenter designed items and can thus be thought of as *experimenter adjusted outcome measures*. The use of *self‐reported outcome measures* is also quite widespread in many of the studies found in the early screening for this review—typically alongside other more objective and reliable outcome measures. The problem here is of course—by definition—risk of self‐reporting bias—typically in the direction of over‐estimating a possible effect of the intervention.

Studies were only included if they included at least one valid and reliable outcome that had been standardised on a different population and was “objective”, that is, not “experimenter‐designed” and not self‐reported. We excluded studies that relied exclusively on self‐reported outcome measures, which had not been based on validated assessment tools. Note that inclusion and exclusion criteria specifically relating to outcomes (experimenter designed and self‐reported) were added as a variation to the Protocol at the third stage of screening.

#### 5.1.5 Duration of follow‐up

All follow‐up durations reported in the primary studies were recorded.

All studies that could be used in the data synthesis reported outcomes in the short run only (with the exception of one study reporting one‐year follow‐up student outcomes); approximately by the end of the intervention.

#### 5.1.6 Types of settings

All types of settings were eligible.

### 5.2 Search methods for identification of studies

#### 5.2.1 Electronic searches

We conducted initial scoping searches in key databases (e.g., ERIC, PsycINFO, SocIndex, Web of Knowledge). We then developed search strategies in an iterative process and, once finalised, conducted all the systematic electronic searches in the following seven databases:

- ERIC (searched through EBSCO‐host)
- PsycINFO (searched through EBSCO‐host)
- SocIndex (searched through EBSCO‐host)
- Academic Search Premier (searched through EBSCO‐host)
- Teacher Reference Center (searched through EBSCO‐host)
- Web of Knowledge (Social Science Citation Index & Science Citation Index) (searched via Thomson Reuters)
- ASSIA (searched through ProQuest)

The results of all of the electronic searches were combined into a master database on a software database specifically designed for processing studies in a SR: EPPI Reviewer 4 (Thomas, Brunton, & Graziosi, 2010). The search strings for each database can be found in Appendix C1.

#### 5.2.2 Searching other resources

##### 5.2.2.1 Grey literature search strategy

In order to identify relevant grey literature for the review (reports, academic theses, working papers, etc.) different strategies were utilised. We searched specific targeted relevant online repositories such as the Danish and U.S. Clearinghouses for educational research (https://ies.ed.gov/ncee/wwc/WhoWeAre). Furthermore, we searched general research repositories (such as Social Care Online) and national research portals such as Forskningsdatabasen (Danish National Research Database), SwePub (Academic content from Swedish universities) and NORA (Norwegian Open Research Archive). Searches on Google Scholar for grey literature were also developed (see Appendix C1).

##### 5.2.2.2 Citation searching

Due to the time restraints of the review‐process, we prioritised citation‐tracking of the most relevant identified studies. We performed citation searching on SRs and meta‐analyses that were included after the second stage (full text) screening. In general, the citation‐tracking was retrospective that is, we searched the bibliography of the relevant studies. We made a judgement to prioritise exhaustive searching and therefore used systematic citation searching to supplement the primary strategy (namely systematic electronic searching).

### 5.3 Data collection and analysis

#### 5.3.1 Selection of studies

Once deduplicated, a random sample of studies was independently triple screened in EPPI at first stage (titles and abstracts only) by three reviewers using the inclusion/exclusion criteria (section “Criteria for considering studies for this review”) by way of quality assurance. The database was then split into equal thirds and each third was double screened by two reviewers. Any disagreements were resolved through discussion, with arbitration where necessary by a third reviewer. If necessary, a fourth reviewer was available to provide confirmation of inclusion/exclusion. Potentially relevant studies (i.e., studies remaining after title and abstract screening) were located and retrieved. Once retrieved all full papers were double screened at second stage, with arbitration (where necessary) as described above. All included studies were rescreened at third stage. This stage of screening was added as a variation to the protocol to exclude studies that only used experimenter designed or self‐reported outcomes, as these kinds of outcomes are susceptible to the introduction of bias.

None of the reviewers were blind to the authors, institutions, or the journals responsible for the publication of the articles.

#### 5.3.2 Data extraction and management

Two main topic areas emerged: language and literacy development; and social and emotional development with an additional one minor topic area also present.

Detailed data extraction of the studies included was undertaken, including information about participants, settings, intervention, control or comparison conditions, sample size, time period, outcomes and results. Data extraction, risk of bias assessment and extraction of numerical data for effect size calculation and pooling of effect sizes in the meta‐analyses were all undertaken by at least two reviewers working in pairs. Disagreements were resolved by discussion. Extracted data were stored electronically. Analysis was conducted in RevMan 5.

#### 5.3.3 Assessment of risk of bias in included studies

A modified version of the risk of bias model developed by Prof. Barnaby Reeves in association with the Cochrane Non‐Randomised Studies Method group (Reeves, Deeks, Higgins, & Wells, 2011) was used to assess the risk of bias in the studies included in the in‐depth review. This model, an extension of the Cochrane Collaboration's risk of bias tool, covers risk of bias both in RCTs and in nonrandomised studies that have a well‐defined control or comparison group.

The intention was that the modified version of this model addressed the following nine risk‐ of‐bias judgement items:

##### 5.3.3.1 Risk‐of‐bias judgement items

- Sequence generation (judged on a low/high risk/unclear scale)
- Allocation concealment (judged on a low/high risk/unclear scale)
- Confounders (judged on a 5‐point scale/unclear)
- Blinding (judged on a 5‐point scale/unclear)
- Incomplete outcome data (judged on a 5‐point scale/unclear)
- Selective outcome reporting (judged on a 5‐point scale/unclear)
- Other potential threats to validity (judged on a 5‐point scale/unclear)
- A priori protocol (judged on a yes/no/unclear scale)
- A priori analysis plan (judged on a yes/no/unclear scale)

On a 5‐point scale, 1 corresponds to low risk of bias and 5 to a high risk of bias. A score of 5 on any of the items assessed on the 5‐point scale translates to a risk of bias so high that the findings were not considered in the data synthesis because they are more likely to mislead than inform. Quality appraisal of the included studies preceded any declaration of results.

#### 5.3.4 Measures of treatment effect

For continuous outcomes, an effect size with 95% CIs was calculated. Hedges' *g* was used for estimating the SMD and we applied the small *N* correction. Hedges' (adjusted) *g* and its standard error are calculated as (Lipsey & Wilson, 2001, pp. 47–49):

$$g=\left(1-\frac{3}{4N-9}\right)\left(\frac{X_{1}-X_{2}}{s_{p}}\right),\;SE_{g}=\sqrt{\frac{N}{n_{1}n_{2}}+\frac{g^{2}}{2N}},$$

where $N=n_{1}+n_{2}$ is the total sample size, *X* denotes the (adjusted) mean of a group, and *s*_p_ is the pooled standard deviation defined as:

$$s_{p}=\sqrt{\frac{(n_{1}-1)s_{1}^{2}+(n_{2}-1)s_{2}^{2}}{\left(n_{1}-1)+(n_{2}-1\right)}},$$

here, $s_{1}$ and $s_{2}$ denotes the standard deviation of the two groups.

When data were not available we extracted the effect size from auxiliary statistics. By using standard techniques (Lipsey & Wilson, 2001) we were able to construct an effect size.

Software for storing data and statistical analyses were Excel, STATA and RevMan 5.0.

#### 5.3.5 Unit of analysis issues

To account for possible statistical dependencies, we examined a number of issues: whether individuals were randomised in groups (i.e., cluster randomised trials), whether individuals had undergone multiple interventions, whether there were multiple treatment groups, and whether several studies were based on the same data source.

##### 5.3.5.1 Cluster randomised trials

We checked for consistency in the unit of allocation and the unit of analysis, as statistical analysis errors can occur when they are different. Whilst ignoring clustering will not produce biased estimates of intervention effects it will bias the standard errors and make something appear statistically significant, when in truth the observed difference could be largely due to chance. In cases where study investigators had not applied appropriate analysis methods that control for clustering effects in analyses of student outcomes, we used intracluster correlations (ρ) values of 0.05, 0.1 and 0.22 (Donner, Piaggio, & Villar, 2001) and corrected the effect size and standard error.^4^ In cases where study investigators had not applied appropriate analysis methods that control for clustering effects in analyses of teacher/professional outcomes, we used intracluster correlations (ρ) values as reported in the included studies depending on the outcome measure. We report the corrected results and the noncorrected results. We used the following formulas (see Hedges, 2007, p. 349):

$$d=\left(\frac{MD}{SD}\right)\sqrt{1-\frac{2(n-1)\rho }{N-2}},$$

$$SE=\sqrt{\left(\frac{N^{T}+N^{C}}{N^{T}N^{C}}\right)(1+(n-1)\rho )+d^{2}\left(\frac{\left(N-2){\left(1-\rho \right)}^{2}+n(N-2n)\rho^{2}+2(N-2n)\rho (1-\rho \right)}{2(N-2)[(N-2)-2(n-1)\rho ]}\right)},$$

where n is cluster size and $N^{T},N^{C}$ are treatment and control group sample sizes and N is total sample size.

##### 5.3.5.2 Multiple interventions groups and multiple interventions per individuals

Several studies reported more than one effect estimate, separated by subgroups of participants, several student academic achievement outcomes or subscales of the outcome measure. When a study reported multiple intervention groups and one control group, we pooled groups if appropriate (if they included different individuals) and compared it to the control group. A synthetic (average) effect size was calculated and used in the analysis to avoid dependence problems. This method provides an unbiased estimate of the mean effect size parameter but overestimates the standard error. Random effects models applied when synthetic effect sizes are involved actually perform better in terms of standard errors than do fixed effects models (Hedges, 2007). However, tests of heterogeneity when synthetic effect sizes are included are rejected less often than nominal.

##### 5.3.5.3 Multiple interventions per individual

There were no studies with multiple interventions per individual used in the analysis.

##### 5.3.5.4 Multiple studies using the same sample of data

Four trials were reported in several studies. We reviewed all studies, but in the meta‐analysis we only included one estimate (per outcome) of the effect from each trial in order to avoid dependencies between the “observations” (i.e., the estimates of the effect) in the meta‐analysis. The choice of which estimates to include was based on our risk of bias assessment of the studies. We chose the estimate from the study that we judged to have the least risk of bias.

##### 5.3.5.5 Multiple time points

All studies reported results by the end of the intervention.

#### 5.3.6 Dealing with missing data

The reviewers assessed missing data rates in the included studies in accordance with the risk of bias tool used (see section Risk of bias assessment). We did not request information from the principal investigators if not enough information was provided to calculate an effect size and standard error.

#### 5.3.7 Assessment of heterogeneity

Heterogeneity among primary outcome studies was assessed with *χ* ^2^ (*Q*) test, and the *I* ^2^, and *τ* ^2^ statistics (Higgins, Thompson, Deeks, & Altman, 2003). Any interpretation of the *χ* ^2^ test was made cautiously on account of its low statistical power. Values of *τ* ^2^ and *I* ^2^ were also, interpreted with caution. The DerSimonian and Laird estimate of *τ* ^2^ is on average overestimated and when the number of studies is small the bias can be substantial (Borenstein, Hedges, Higgins, & Rothstein, 2010). The value of *I* ^2^ is sensitive to the precision of the primary studies effect sizes, in the sense that the more precisely the primary studies effect sizes are estimated the higher the values of *I* ^2^, all else equal (Rücker, Schwarzer, Carpenter, & Schumacher, 2008).

#### 5.3.8 Assessment of publication biases

We used funnel plots (where possible) for information about possible publication bias (Higgins & Green, 2008). Only analyses with at least five studies included were examined. Publication bias is difficult to assess because asymmetric funnel plots are not necessarily caused by publication bias (and publication bias does not necessarily cause asymmetry in a funnel plot).

#### 5.3.9 Data synthesis

As different computational methods may produce effect sizes that are not comparable, we were transparent about all methods used in the primary studies (research design and statistical analysis strategies) and used caution when synthesising effect sizes.^5^

The synthesis for the in‐depth review combined the results meta‐analytically (as it was deemed appropriate to use quantified outcomes synthesis), focusing on outcomes targeting specific groups of participants (professionals and students) within the topics of social and emotional development and language and literacy development respectively.

We carried out our meta‐analyses using the SMDs. Hedges' *g* was used for estimating the SMD and we applied the small *N* correction (Lipsey & Wilson, 2001, pp. 47–49). All analyses were inverse variance weighted using random effects statistical models that incorporate both the sampling variance and between study variance components into the study level weights. Random effects weighted mean effect sizes were calculated using 95% CIs. Analysis was conducted in RevMan 5 (Informatics, 2016) and results displayed graphically in forest plots.

Studies that were coded with a very high risk of bias (scored 5 on the risk of bias scale) were not included in the meta‐analysis.

#### 5.3.10 Sensitivity analysis

In cases where study investigators had not applied appropriate analysis methods that control for clustering effects a sensitivity analysis was undertaken adjusting for clustering.

Sensitivity analysis was further used to evaluate whether the pooled effect sizes were robust across study design and components of methodological quality. For methodological quality, we performed sensitivity analysis for the Blinding, Incomplete outcome data, Selective reporting, and Other bias items of the risk of bias checklists, respectively.

## 6 RESULTS

### 6.1 Description of studies

#### 6.1.1 Results of the search

##### 6.1.1.1 Systematic searches

The electronic searches were completed in seven databases; additionally, grey literature was searched for in seven different locations. All searching took place between April 2017 and December 2018. The searches identified a total of 6,163 records. After deduplication, 5,146 records remained for first stage screening.

##### 6.1.1.2 Citation searches

Upon completion of second stage of screening, eight SRs or meta‐analyses remained (Dunst et al., 2015; Gaudin & Chalies, 2015; Hwang, Bartlett, Greben, & Hand, 2017; Kelcey & Phelps, 2013; Lander, Eather, Morgan, Salmon, & Barnett, 2017; Markussen‐Brown et al., 2017; Snell, Dowsell Forston, Stanton‐Chapman, & Walker, 2013; Yoon, Duncan, Lee, Scarloss, & Shapley, 2007). Four reviews to citation search were also added from the EPPI publication page found at https://eppi.ioe.ac.uk/cms/Default.aspx?tabid=274 (Cordingley, Bell, Evans, & Firth, 2005; Cordingley, Bell, Isham, Evans, & Firth, 2007; Cordingley, Bell, Rundell, Evans, & Curtis, 2003; Cordingley, Bell, Thomason, & Firth, 2005). An expert in the field identified one SR (Basma & Savage, 2018) and one “tertiary” review (Cordingley et al., 2015) from which one met our inclusion criteria (Timperley et al. (2007) and was judged to be consistently robust in all aspects of methodology. The review authors in addition identified two SRs (Egert et al. 2018; Kraft et al. 2018).

The citation searches of the 16 records above, added 56 studies to the third stage screening.

##### 6.1.1.3 Screening at first, second and third stages

The figure in Appendix C3 shows the flow of records through the SR process using a PRISMA flow diagram (Moher et al. 2009). Intercoder agreement at first stage screening (title and abstract) was over 90% in all pairings of reviewers (range: 90–97%). A total of 48,480 records were excluded at first stage screening, leaving 298 records eligible for full text screening, two of which were not available. Thus 296 records were screened for inclusion at second stage (full text).

At second stage screening, full texts were assessed for inclusion based on the criteria set out in section “Criteria for considering studies for this review”. Inter‐rater reliability at this stage (include/exclude only) was lower than at first stage screening, but all disagreements were resolved by a third reviewer and all parties agreed before coding was finalised. In total, 173 records were excluded at second stage, two were unavailable, which left 104 empirical studies remaining. These were combined with 56 empirical studies from citation searching meaning that in total 160 records were taken forward to screening at third stage prior to data extraction which led to 112 additional studies being excluded (see Table 2 for reasons) and 51 studies (including three additional records identified (Table 1)). All 51 studies were coded as having an “education” focus.

**Table 1:** Included records (type and focus) after second and third stage screening (including citation searches the 15 SR/MA)

Stage of screening	Total number of studies	Topic	Record type
			Empirical
Studies remaining after second stage screening	104	Education	101
		Social welfare	3
		Crime and justice	–
Studies from citation searches (added before third stage screening^a^)	56	Education	56
Studies screened at third stage	160	Education	104
		Social welfare	3
Studies remaining after third stage screening	48	Education	48
		Social welfare	0
		Crime and justice	–
Studies added	3	Education	3
Total number of studies	51	Education	51

^a^ From the eight SR/MA/TR as above, plus four reviews from the EPPI website and the additional four systematic reviews identified by an expert and review authors (16 citation searched in total). Seventy‐five records were first and second stage screened manually, and the remaining 56 studies were screened at third stage.

**Table 2:** Reasons for exclusion at third stage

	Number of records excluded
*Reason for exclusion third stage screening*	
Lack of clarity in reporting results or results not reported (e.g., trial protocol)	10
Lack of clarity in describing control condition or control group absent	4
Intervention (does not fit stated definition of PD)	21
Lack of baseline equivalence	6
Exclude on topic (e.g., focusses entirely on health) or focus (e.g., teacher burnout, motivation…) as per protocol	6
Exclude on study design	14
*Exclude on outcome measures*	
Experimenter designed or adjusted outcome measures	28
Outcome measures not validated	4
Self‐report outcome measures only	6
Other reason for exclusion on outcome measure	13
Total	112

Abbreviation: PD, professional development.

The most striking result of the process of searching and screening to inclusion at third stage is that, of the 51 included empirical studies all were in the area of education. This was despite searching exhaustively to include any relevant studies in all three areas. It is possible that empirical studies have been undertaken to evaluate the effectiveness of CPD interventions in the areas of social welfare and crime and justice, but that they did not meet our strict inclusion criteria. So, for example they could have used a research design without an appropriate control or comparison group, or they could have used experimenter designed or nonvalidated outcome measures (both of which types of outcomes were excluded from our review).

The studies focused on PD in a total of three topic areas, although most were in two overarching topic areas: 12 in PD in social and emotional development interventions and 38 in PD in language and literacy development interventions One study looked at PD in another topic: stress reduction (see Table 3). In the social and emotional development area two trials were reported in two papers each, thus the number of trials was 10. In language and literacy, the number of trials was 33; two trials were reported in two papers each and one trial was reported in four papers.

**Table 3:** Topics of studies and trials included in the review

Topics	Number of studies	Number of trials
Social and emotional development	12	10
Language and literacy development	38	33
Stress reduction	1	1
Overall total	51	44

#### 6.1.2 Descriptive data extraction of included studies

##### 6.1.2.1 Social and emotional development

Common features of the *social and emotional development* PD focused on: developing teachers' language use, emotional support and positive behaviour‐management strategies in the classroom; strengthening teachers' interactions with the children; individualising responses to children and improving teacher/child interactions; improving classroom management skills and creating positive, supportive learning environments; and generally developing teachers' abilities to increase their expectations of children and young people (see Appendix D for more details). Five trials evaluated a “branded” intervention: Incredible Years Teacher Classroom Management Programme. Also evaluated were videotaping of classroom interactions and feedback and evidence‐based strategies to improve teacher expectations of students.

Table 4 present the study characteristics for the 10 trials in the social and emotional development topic area. Five of the 10 trials were undertaken in the United States; and one trial was undertaken in each of the following countries: Denmark, Ireland, the Netherlands, New Zealand and Portugal. The settings ranged from preschool (five trials), through kindergarten (one trial) and elementary secondary schools (four trials), with most in early childhood settings; participants were teaching professionals and children and young people in these settings. Although there was some individual variation in the delivery models of the professional development (specifically in relation to dosage and timing), the basic components were very similar across all 10 trials and included the following components: workshop‐based training with resources, personalised coaching/consultation using feedback on observations or videotapes of classroom practice, feedback and reflection. The length was typically one school year with a mean of 0.91 year. Control conditions were also very similar and comprised business as usual PD (half of the trials with wait list design). The table in Appendix D provides additional, detailed information.

**Table 4:** Study characteristics, social and emotional development

Characteristics		Number of studies
Country	USA	5
	Denmark	1
	Ireland	1
	The Netherlands	1
	New Zealand	1
	Portugal	1
Setting	Preschool	5
	Kindergarten	1
	Elementary	4
	Secondary	0
Components of intervention	Workshop‐based training with resources	7^a^
	Personalised coaching/consultation using feedback on observations or videotapes of classroom practice	7
	Feedback and reflection	2
Length	Mean years (*SD*)	0.91 (0.48)
	Range	3 months to 2 years
Control condition	Business as usual	5
	Wait list	5

^a^ Of these, four also involved coaching and one also involved other feedback.

##### 6.1.2.2 Language and literacy development

Common features of the *language and literacy* PD focused on: developing teachers' knowledge and understanding in the substantive fields of reading and writing development (in two cases explicitly using evidence from research). Specifically, PD aimed to develop teachers' instructional strategies, methods and techniques (in the substantive area); teachers' abilities to differentiate or individualise instruction; teachers' abilities to support children generally in their language and literacy development; teachers' confidence and their abilities to interact responsively with the children; and finally, to fill in the gaps in teachers' conceptual knowledge and understanding. eleven studies evaluated a number of “branded” interventions, for example: Project RIME; Learning Language and Loving It (two trials); LEEP the Literacy Environment Enrichment Program; Exceptional Coaching for Early Language and Literacy (ExCELL) (three trials); PAVEd for Success; Responsive Classroom (two trials) and Making the Most of Classroom Interactions and My Teaching Partner (four trials) (see Appendix D).

Table 5 present the study characteristics for the 33 trials included in the language and literacy development area. Twenty‐eight of the 33 trials were undertaken in the United States; and one trial was undertaken in each of the following countries: Australia, Chile and Germany; and two trials were undertaken in the UK. The settings ranged from preschool, through elementary school and one study was conducted in autism‐specific classes/units or schools. Most were in early childhood settings; participants were teaching professionals and children and young people in these settings with the exception of five trials who focused on Latino dual language learner children, children who were native English speakers, children attending autism‐specific classes and special education students with LD respectively. Although there was some individual variation in the delivery models of the professional development (specifically in relation to dosage and timing), the basic components were very similar across all 33 trials and included the following components: workshop‐based training with resources, personalised coaching/consultation using feedback on observations or videotapes of classroom practice, other feedback and reflection. The length was typically one school year with a mean of 1.26 year. Control conditions were also very similar and comprised business as usual PD (sometimes with wait list design) and some were characterised as PD without the same focus and content as the experimental PD. The table in Appendix D provides additional, detailed information.

**Table 5:** Study characteristics, language and literacy development

Characteristics		Number of studies
Country	USA	28
	Australia	1
	Chile	1
	Germany	1
	UK	2
Setting	Preschool	15
	Kindergarten	5^a^
	Elementary	11
	Secondary	3
	Other	1
Student eligibility criteria specified other than grades	Latino DLL	2
	Children attending autism‐specific classes/units or schools. Aged between 4 and 11 years	1
	Only children who were native English speakers according to parental report	1
	Special education students with LD	1
Components of intervention	Workshop‐based training with resources	30^b^
	Personalised coaching/consultation using feedback on observations or videotapes of classroom practice	26
	Feedback and reflection	6
Length	Mean years (*SD*)	1.26 (0.59)
	Range	7 weeks to 3 years
Control condition	Business as usual	25
	Wait list	3
	Some PD but not with the same focus and content as experimental	5

Abbreviation: DLL, dual language learner.

^a^ Of these, two also evaluated preschool and elementary respectively.

^b^ Of these, 24 also involved coaching and four also involved other feedback.

##### 6.1.2.3 Stress reduction

Also included in the review, is one study exploring stress reduction of teachers; and teaching quality. The topic was evaluated by only one RCT in the United States with elementary school teachers participating. The intervention included a one‐day workshop and weekly group practice and instruction lasting eight weeks.

### 6.2 Risk of bias in included studies

The ratings of each study in relation to the nine domains in the risk of bias tool as well as the descriptions used for the assessments are shown in Appendix E. The risk of bias judgements are based on prespecified questions and a 5‐point scale (except the items sequence generation and allocation concealment) with ratings of 1 = low risk and 5 = high risk. A score of 5 on any of the risk of bias items rated on a 5‐point scale corresponds to a risk of bias so high that the findings of the study should not be considered in the data synthesis. Further details on risk of bias are provided in the design and methods section.

#### 6.2.1 Social and emotional development

Ten RCTs were included, see Table 6. The trials were reported in 12 papers. The two studies Reinke et al. (2016, 2018) reporting on the same trial had almost identical content and the same applies to the two studies Murray, Rabiner, and Carrig (2014) and Murray, Rabiner, Kuhn, Pan, and Sabet (2018) reporting on the same trial. The summary risk of bias is therefore only shown for ten studies.

**Table 6:** Summary risk of bias score, social and emotional development

Risk of bias items	Judgement								Total number of studies
	High	Low	Unclear	1	2	3	4	5	
Sequence generation	0	4	6						10
Allocation concealment	0	4	6						10
Blinding^a^			0	0	1	5	4	0	10
Incomplete data^a^			1	3	5	1	0	0	10
Selective reporting^a^			0	3	2	5	0	0	10
Other bias^a^			1	0	6	0	3	0	10

^a^ The judgement is based on a 5‐point scale where 1 indicates low risk of bias and 5 indicates high risk of bias. Studies scoring 5 on any item of the risk of bias tool were not included in the data synthesis.

Overall, the included studies varied on risk of bias judgements and no single study could be characterised as a robust RCT with low risk of bias on all assessed risk of bias items, although one study had only minor problems.

Four studies reported the use of appropriate randomisation methods; the remaining studies did not report the method of randomisation. As is common in social intervention, it is generally impossible to blind participants or those delivering the interventions. Six studies clearly stated that outcome assessors were blinded to allocation status and one study further stated that the statistical analyses of data was conducted centrally. Overall attrition levels were not high, only one study had relatively high levels of attrition and one study did not report attrition levels.

Three studies were free of selective reporting bias. Three of the studies had serious problems of various kinds rated 4 on the “other risk of bias” item.

We could not locate a protocol or an a priori analysis plan for any of the studies. Confounding was not relevant since we did not find any nonrandomised studies on social and emotional development to include.

#### 6.2.2 Language and literacy development

Thirty RCTs were included, see Table 7. The trials were reported in 35 papers; Cabell et al. (2011) and Piasta et al. (2012) reported on the same RCT but reported different outcomes; Rimm‐Kaufman et al. (2014) and Ottmar et al. (2013) reported on the same RCT but different outcomes; Pianta et al. (2017), Sandilos et al. (2018), Hamre et al. (2012) and Ansari and Pianta (2018) reported on the same trial, two of these four studies reported the same student outcomes and the other two reported the same teacher outcomes, therefore, only two of these four studies are shown in the summary risk of bias. Three studies used a nonrandomised design and attempted to control for confounding factors using other statistical methods. Overall, the included studies varied on risk of bias judgements and no single study could be characterised as a robust RCT with low risk of bias on all assessed risk of bias items.

**Table 7:** Summary risk of bias score, language and literacy development

Risk of bias items	Judgement								Total number of studies
	High	Low	Unclear	1	2	3	4	5	
Sequence generation	9	5	22						36
Allocation concealment	9	5	22						36
Blinding^a^, ^b^					1	9	23		33
Incomplete data^a^, ^b^			6	2	11	8	3	3	33
Selective reporting^a^, ^b^				16	2	7	7	1	33
Other bias^a^			4	4	6	7	3	12	36
Confounding^a^, ^c^						1		2	3

^a^ The judgement is based on a 5‐point scale where 1 indicates low risk of bias and 5 indicates high risk of bias. Studies scoring 5 on any item of the risk of bias tool were not included in the data synthesis.

^b^ Not judged for the three studies where treatment effect could not be separated from school or center effect.

^c^ Not judged for the thirty three studies using a randomised design.

Five studies reported the use of appropriate randomisation methods; the remaining studies did not report the method of randomisation or did not randomise. Six randomised studies were rated high on sequence generation and allocation concealment, even though the sequence generation method was not reported. However, in three studies it was reported that only one centre or school was allocated to control. In neither of these studies was it possible to separate the intervention effect from the centre or school effect. In another randomised study it was reported that classrooms were randomised and teachers were assigned to the selected classrooms, determining if she or he was eligible for participation, and replacing any classes where the teacher was ineligible. This is not proper randomisation, as as there is nonrandom selection of teachers and classrooms into treatment after randomisation. In two randomised studies, schools were randomised after which teachers selected one of their reading groups to participate and in the other study schools allocated to treatment had complete autonomy over which teachers they chose for participation. This is not proper randomisation.

As is common in social intervention, it is generally impossible to blind participants or those delivering the interventions. Ten studies clearly stated that outcome assessors were blinded to allocation status. Overall attrition levels were high; only 13 studies had relatively low levels of attrition. Three studies scored 5 on the incomplete outcome data item (see Appendix E for details). It was not possible to judge the incomplete data item in six studies as they provided too little (if any) information. One study was rated 5 on the selective reporting item. In addition, seven studies had serious problems and were rated 4 on the selective reporting item. Twelve studies were rated 5 on the “other risk of bias” item (for details see Appendix E). In addition, three of the studies had serious problems of various kinds rated 4 on the “other risk of bias” item and four studies provided too little information to be judged on the “other risk of bias” item. Two of the three nonrandomised studies were rated 5 on the confounding item as they did not adequately control for confounding factors.

In total 16 studies were given a score of 5 on at least one of the risk of bias items, corresponding to a risk of bias so high that the findings should not be considered in the data synthesis.

We could not locate a protocol for any of the studies.

#### 6.2.3 Stress reduction

One RCT was included. 18 teachers were randomised, however, the method of randomisation was not reported. The study had serious problems of various kinds and was rated 5 on the “other risk of bias” item corresponding to a risk of bias so high that the findings should not be considered in the data synthesis (for details see Appendix E).

### 6.3 Synthesis of results

#### 6.3.1 Numerical data extraction social and emotional development

One study could not be included in the meta‐analysis as there was uncertainty on how the reported standard deviations were calculated. An e‐mail was sent to the first author to clarify the uncertainty concerning standard deviations February 13, 2019, however, we have not received a reply.

Table 8 present the numerical data extraction for the nine studies on social and emotional development that were included in the meta‐analysis.

**Table 8:** Numerical data for social and emotional development studies

Outcomes reported on	Students	6 studies
	Teachers	4 studies
Number of students^a^	Mean (*SD*)	914 (630)
	Range	99–1,685
Number of classes^b^	Mean (*SD*)	95 (61)
	Range	22–224
Number of schools/centres^c^	Mean (*SD*)	26 (18)
	Range	9–58
Time point	End of intervention	9

^a^ Not reported in three studies.

^b^ Not reported in two studies.

^c^ Not reported in four studies.

Six studies reported student outcomes using standardised measures of various kinds. Four studies reported on student academic outcomes using standardised measures. Further, student's socioemotional skills were measured by preschool teachers assessment of each child using the Strengths and Difficulties Questionnaire (SDQ; Goodman, 1997) in two studies and student social‐emotional and behavioural outcomes were measured in two studies using teacher ratings of emotion regulation, prosocial behaviour and inattention on the Revised Teacher Social Competence scale (R‐TSC). One study measured student's school readiness, social skills and problem behaviour using the Preschool and Kindergarten Behavior Scales‐2 (PKBS‐2); one study measured inattention using the Conners' DSM‐IV Inattention scale (Conners, 2001) and one study measured student's disruptive behaviour and concentration problems using the Teacher Observation of Classroom Adaptation‐Checklist (TOCA‐C). In all other studies, children's socioemotional outcomes were not assessed using standardised measures.

Four studies reported outcome measures of teachers; three studies reported various measures of the Classroom Assessment Scoring System (CLASS) and one study reported other measures of teacher outcomes (caregiving behaviour)

The sample sizes reported in the studies varied between 99 students to 1,685 students with an average of 914 students; 22 classes to 224 classes with an average of 95 and nine schools to 58 schools with an average of 26 schools. All studies reported outcomes by the end of the intervention. Further details of the numerical data extraction are shown in Appendix F.

#### 6.3.2 Numerical data extraction language and literacy development

Sixteen studies were given a score of 5 on at least one of the risk of bias items, corresponding to a risk of bias so high that the findings should not be considered in the data synthesis. In addition, two studies could not be included in the meta‐analysis as there was uncertainty on how the reported standard deviations were calculated. An e‐mail was sent to the authors to clarify the uncertainty concerning standard deviations December 6, 2018 and December 12, 2018 respectively, however, we have not received any replies. Finally, one study did not report results in a format that could be used in the meta‐analysis. Appendix F provides more details on the data extraction of these studies.

Table 9 present the numerical data extraction for the 17 studies on language and literacy development that were included in the meta‐analysis. All studies reported either student or teacher outcomes that enabled the calculation of a SMD and standard error approximately by the end of the intervention.

**Table 9:** Numerical data for language and literacy development studies

Outcomes reported on	Students	13
	Teachers	8
Number of students^a^	Mean (*SD*)	1,632 (1,333)
	Range	164–4,078
Number of classes^b^	Mean (*SD*)	113 (91)
	Range	24–324
Number of schools/centres^c^	Mean (*SD*)	58 (60)
	Range	4–224
Time point	End of intervention	17

^a^ Not reported in three studies.

^b^ Not reported in two studies.

^c^ Not reported in six studies.

Thirteen studies reported on various student academic outcomes using standardised measures. Eight studies reported on teacher outcomes; four using the ELLCO Toolkit, although one study used one of three subscales of the ELLCO only. Another three studies reported summary measures of the CLASS and one study reported other measures of teacher outcomes (mathematics teaching practices). Many of the studies in this topic area either did not assess professionals' outcomes at all or they did so using experimenter designed or nonstandardised outcomes.

The sample sizes reported in the studies varied between 164 students to 4,078 students with an average of 1,632 students; 24 classes to 324 classes with an average of 113 and four schools to 224 schools with an average of 58 schools. Further details of the numerical data extraction are shown in Appendix F.

#### 6.3.3 Meta‐analyses

All studies reported either student or teacher outcomes that enabled the calculation of a SMD and standard error approximately by the end of the intervention.

Due to the homogeneity of PD approaches in the two topic areas “social and emotional development” and “language and literacy development”, we used professional and student outcomes in the two topic areas as the basis of the meta‐analyses presented below.

We report the results of a series of meta‐analyses below, where individual studies with homogeneity of outcome are combined to obtain an “overall” effect size estimate of the interventions where possible. If outcomes are too different to combine in a meta‐analysis the study‐level effect sizes are shown. All outcomes are measured such that a positive effect size favours the treated.

##### 6.3.3.1 Social and emotional development: student outcomes

**Figure 1:**
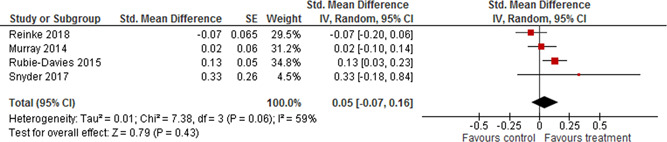
Student academic scores

The results of the four studies reporting results on student academic outcomes were combined in a meta‐analysis as displayed in Figure 1.

The meta‐analysis of the studies showed evidence of some statistical heterogeneity with an *I* ^2^ value of 59% and the estimated *τ* ^2^ is 0.01.^6^ All effect sizes except one favour the treated group, the weighted average is not statistically significant. The weighted average SMD is 0.05 (95% CI [−0.07, 0.16]). However, given there are relatively few studies and some heterogeneity between them, some caution is needed in making an assumption that there is no effect from PD on student academic outcomes.

The study by Reinke et al. (2018) reported an ICC, which we used to adjust their result for clustering. The remaining three studies did not adjust for clustering nor report an ICC.

A sensitivity analysis was undertaken adjusting for clustering using an ICC of 0.05, 0.1 and 0.22. The resulting forest plots (Figures G1–G3 in Appendix G) show that the result (as expected) does not change.

An insufficient number of studies reported on student academic outcomes to perform sensitivity analysis of methodological quality.

**Figure 2:**
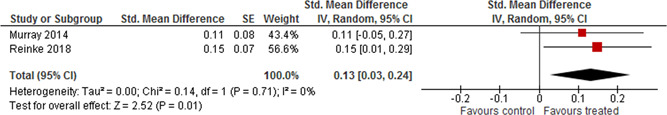
Student social competences

Two studies reported outcomes on student social competences using the Social competence (R‐TSC). The outcomes were combined in a meta‐analysis as displayed in Figure 2. The study by Reinke et al. (2018) reported an ICC, which we used to adjust the results in both studies for clustering. The meta‐analysis of the studies showed no evidence of statistical heterogeneity with an *I* ^2^ value of 0% and the estimated *τ* ^2^ is 0.00, which suggests that despite the studies having some differences in their pedagogical approaches and students, the underlying effect of the interventions is similar. All effect sizes favour the treated group. The weighted average SMD is 0.13 (95% CI [0.03, 0.24]). However, given there are very few studies, some caution is needed in making an assumption that there is a single true effect from PD on student social competences.

An insufficient number of studies reported on student social competences to perform sensitivity analysis of methodological quality.

**Figure 3:**
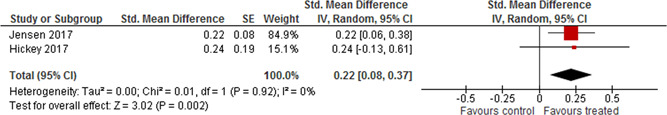
Student socioemotional skills

Two studies reported outcomes on student's socioemotional skills measured by preschool teacher's assessment of each child using the SDQ. The outcomes were combined in a meta‐analysis as displayed in Figure 3. The study by Jensen et al. (2017) took into account clustering and the study by Hickey et al. (2017) reported an ICC, which we used to adjust their results for clustering. The meta‐analysis of the studies showed no evidence of statistical heterogeneity with an *I* ^2^ value of 0% and the estimated *τ* ^2^ is 0.00, which suggests that despite the studies having some differences in their pedagogical approaches and students, the underlying effect of the interventions is similar. All effect sizes favour the treated group. The weighted average SMD is 0.22 (95% CI [0.08, 0.37]). However, given there are very few studies, some caution is needed in making an assumption that there is a single true effect from PD on student social competences.

An insufficient number of studies reported on student social‐emotional skills to perform sensitivity analysis of methodological quality.

**Figure 4:**
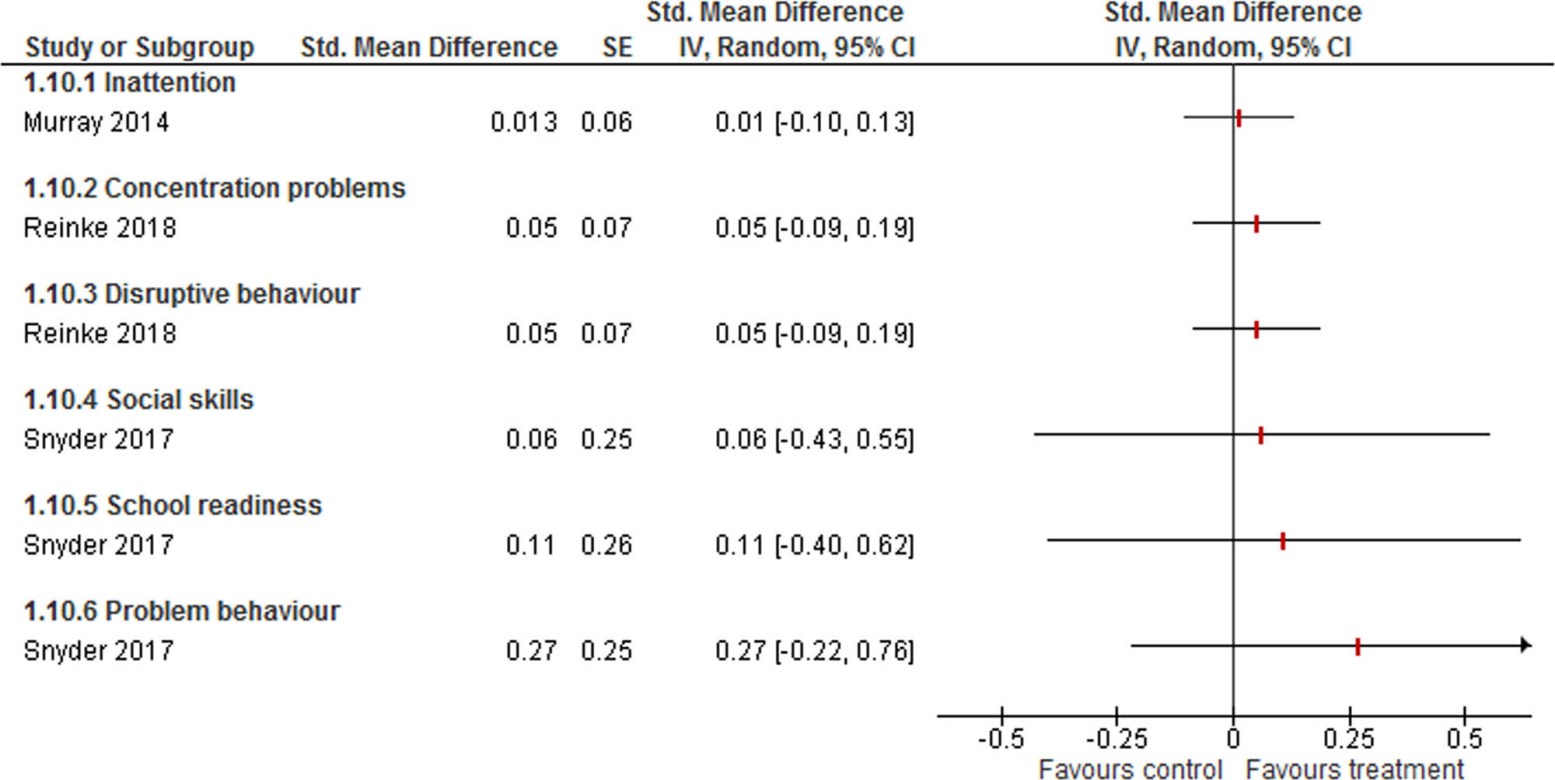
Other student outcomes

Three studies reported outcomes on various other student measures that were too different to be combined. The reported results from the three studies are displayed in Figure 4. All results indicated a positive effect with study‐level effect sizes varying between 0.01 and 0.27. None of the study‐level effect sizes were statistically significant.

##### 6.3.3.2 Social and emotional development: teacher outcomes

**Figure 5:**
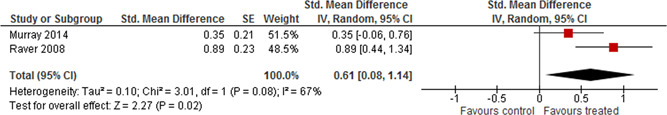
Positive climate

**Figure 6:**
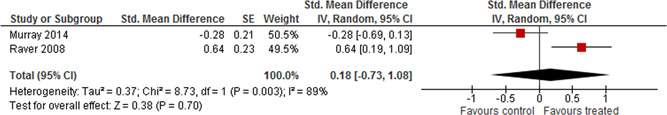
Negative climate

**Figure 7:**
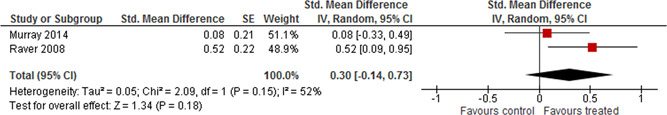
Behaviour management

Two studies reported on three subscales of CLASS (Positive climate, Negative climate and Behavioural management which we combined in a meta‐analysis as displayed in Figures 5, 6, 7. The analysis in the study by Raver et al. (2008) took into account clustering, and the randomisation of teachers were done within schools in the study by Murray et al. (2014); thus there was no need for cluster correction of teacher outcomes. The weighted average effects are all positive but only Positive climate is statistically significant; the weighted average of Negative climate and Behaviour management are statistically nonsignificant.

The weighted average SMD of Positive climate is 0.61 (95% CI [0.08, 1.14]), for Negative climate it is 0.18 (95% CI [−0.73, 1.08]) and for Behaviour management it is 0.30 (95% CI [−0.14, 0.73]). There is a high degree of heterogeneity between the studies in the analysis of Negative climate as indicated by the values of *I* ^2^ and *τ* ^2^, respectively^7^ and there is some degree of heterogeneity in the analyses of Positive climate and Behaviour management.

Given there are only two studies reporting these teacher outcomes, some caution is needed in making an assumption that there is (or is not) a single true effect from PD on any of these teacher outcomes.

**Figure 8:**
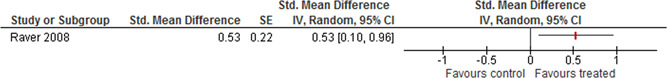
Teacher sensitivity

One study of the studies in addition reported on the subscale Teacher sensitivity, as displayed in Figure 8. The single study effect size is positive and statistically significant.

There were an insufficient number of studies to perform sensitivity analyses of methodological quality.

**Figure 9:**
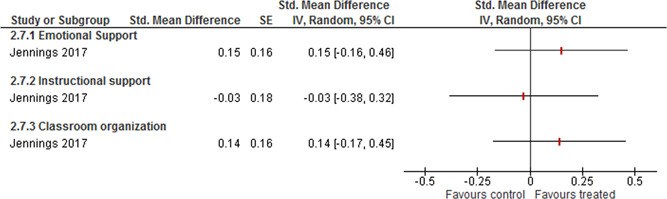
Summary CLASS

The study by Jennings et al. (2017) reports on three summary CLASS measures (Emotional support, Instructional support and Classroom organisation). In Figure 9 the individual study results are shown for the summary measures. Two of the measures are positive and one is negative and none of them are statistically significant.

**Figure 10:**
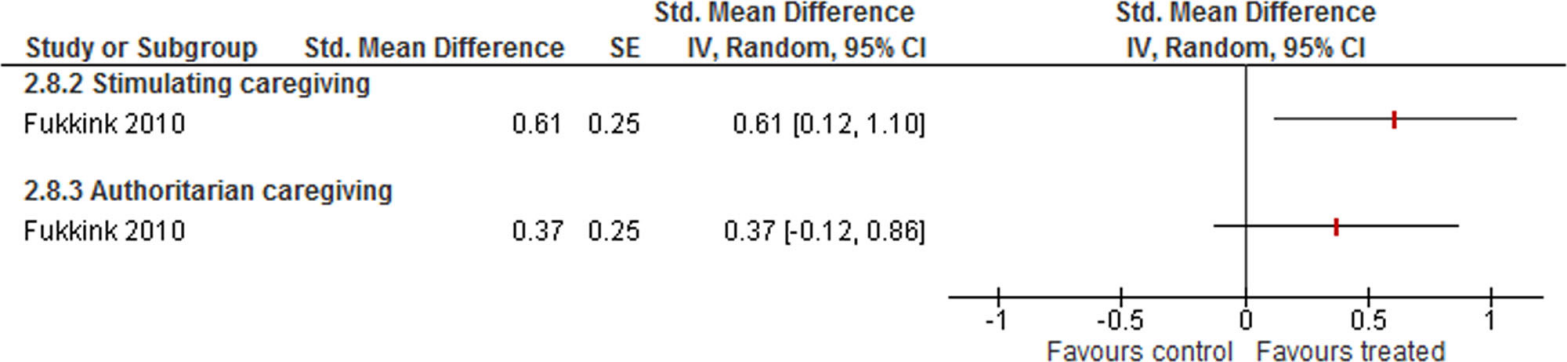
Other teacher outcomes

Fukkink and Tavecchio (2010) reported two measures from the Caregiver interaction scale (Arnett, 1989). The single‐study effect sizes are shown in Figure 10. Both results indicate a positive effect, although only one is statistically significant.

The results reported in Jennings et al. (2017) and in Fukkink and Tavecchio (2010) needed adjustment for clustering. We did not, however, perform any sensitivity analyses as the individual study results were not combined in a meta‐analysis.

##### 6.3.3.3 Language and literacy development: student outcomes

**Figure 11:**
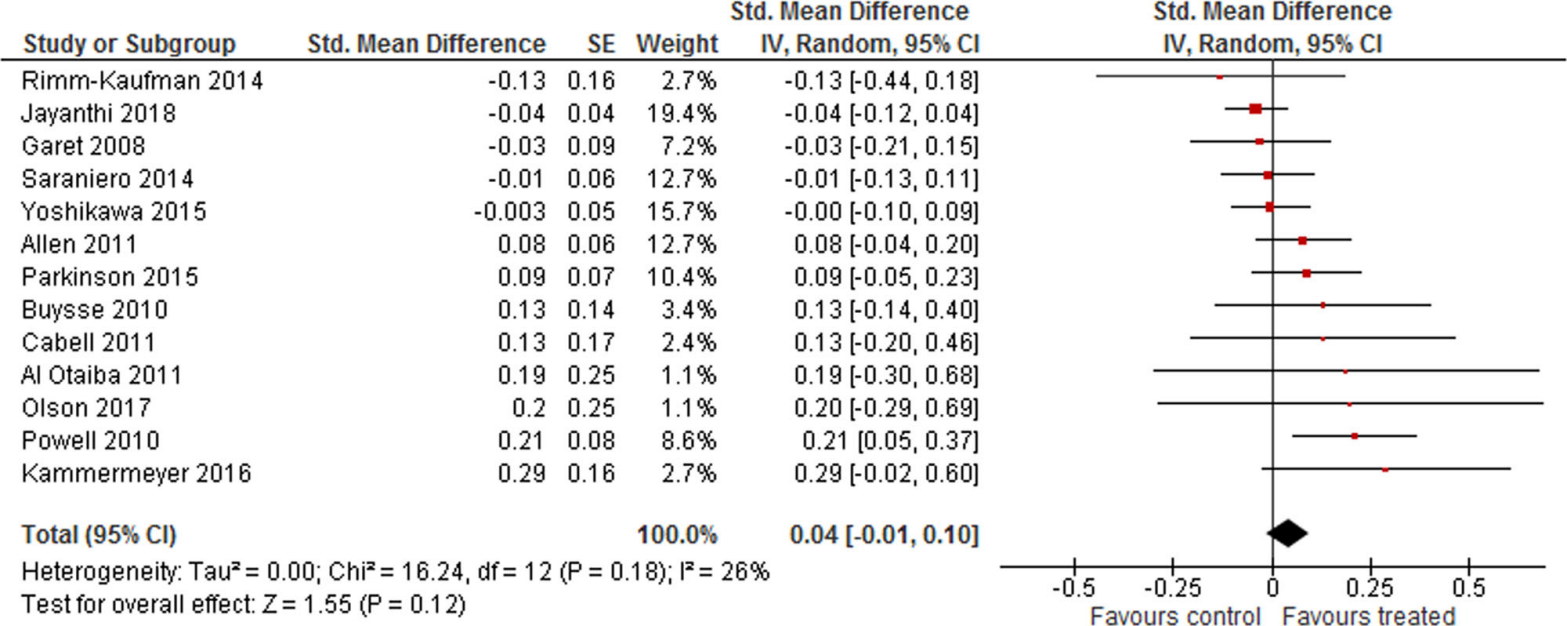
Student academic outcomes

The results of the 13 studies reporting results on student academic outcomes were combined in a meta‐analysis as displayed in Figure 11. The DerSimonian‐Laird estimate of *τ* ^2^ is 0.00 and *I* ^2^ is 26%. As *Q* = 16.24, *p* = .18, there is no evidence of heterogeneity.^8^

The weighted average effect size favours the treated group but is not statistically significant. The weighted average SMD is 0.04 (95% CI [−0.01, 0.10]).

A sensitivity analysis was undertaken adjusting for clustering using an ICC of 0.05, 0.1 and 0.22. Note that the studies by Al Otaiba et al. (2011), Cabell et al. (2011), Garet et al. (2008), Jayanthi et al. (2018), Olson et al. (2017), Parkinson et al. (2015) and Rimm‐Kaufman et al. (2014) either took into account clustering or reported an ICC which we used to correct for clustering; thus in the sensitivity analysis the results reported in these studies were not further adjusted for clustering. The resulting forest plots (Figures G4–G6 in Appendix G) show that (as expected) the overall result does not change. This suggests that, although the overall effect on student academic outcomes is positive, it is not statistically significant.

Sensitivity analyses were planned to evaluate whether the pooled effect sizes were robust across study design and components of methodological quality. All but one study included in the meta‐analysis were RCTs, we evaluated the impact of study design by removing that one study. For methodological quality, we carried out sensitivity analyses for the Blinding, Incomplete outcome data, Selective reporting and Other bias components of the risk of bias checklists, respectively. We examined the robustness of conclusions when we excluded studies with risk of bias scores of 4 and Unclear on Incomplete outcome data, Blinding, Selective reporting and Other bias. The results are provided in Table 10.

**Table 10:** Sensitivity analysis—results

	SMD [CI 95%] (number of studies)
All studies	0.04 [−0.01, 0.10] (13)
Characteristics of studies removed from the analysis:	SMD [CI 95%] with studies removed
Nonrandomised	0.04 [−0.01, 0.10] (12)
Incomplete outcome data score of 4 and unclear	0.05 [−0.02, 0.12] (11)
Blinding bias score of 4	0.13 [0.04, 0.22] (4)
Selective reporting score of 4	0.05 [−0.02, 0.12] (10)
Other bias score of 4 and unclear	0.01 [−0.04, 0.07] (9)

Abbreviations: CI, confidence interval; SMD, standardised mean difference.

There were no appreciable changes in the results due to exclusion of the nonrandomised study, studies with scores of 4 or Unclear on the incomplete outcome data, selective reporting and Other bias components of the risk of bias checklist. The result was somewhat sensitive due to the removal of studies with scores of 4 on the blinding component; the weighted average SMD became larger and statistically significant when studies with blinding scores of 4 where removed. Note, however, that only four studies contributed to the average.

##### 6.3.3.4 Language and literacy: teacher outcomes

**Figure 12:**
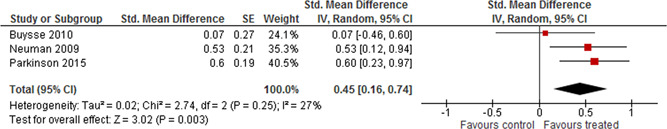
Total ELLCO score. ELLCO, Early Language and Literacy Classroom Observation

Three studies reported results on the total ELLCO score. These were combined in a meta‐analysis as displayed in Figure 12. The pooled effect size favours the treated group and is statistically significant. The weighted average SMD is 0.45 (95% CI [0.16, 0.74]). There is a small degree of heterogeneity between the studies as indicated by the values of *I* ^2^ and *τ* ^2^ (*I* ^2^ is 27% and *τ* ^2^ is 0.02). There were no appreciable changes in results when using profile likelihood and restricted maximum likelihood to estimate the between‐study variance.

Given there are only three studies reporting teacher outcomes measured by the full ELLCO and some heterogeneity is present we cannot conclude on the effect from PD on this teacher outcome.

The unit of randomisation was the same as the unit of analysis in Buysse et al. (2010) and Neuman and Cunningham (2009) and Parkinson et al. (2015) adjusted for clustering, so there was no need of cluster adjustment. There were an insufficient number of studies to perform sensitivity analyses of methodological quality.

**Figure 13:**
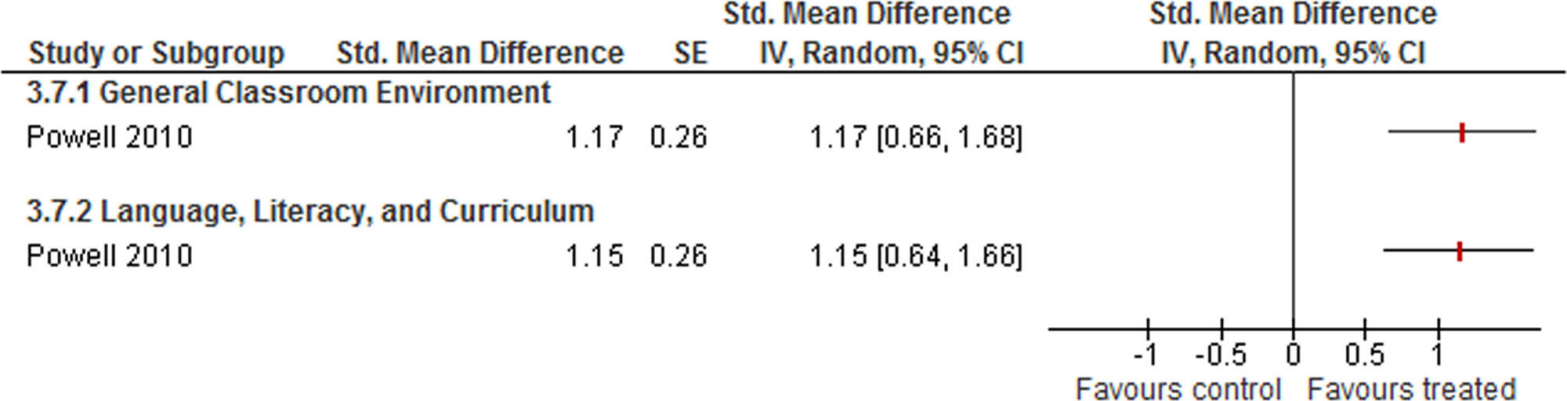
The General Classroom Environment and the Language, Literacy and Curriculum subscales of the ELLCO, teacher outcomes. ELLCO, Early Language and Literacy Classroom Observation

The single‐study effect sizes of the classroom observation subscales of ELLCO (reported in Powell et al., 2010) are shown in Figure 13. The effects are very large, positive and statistically significant.

We did not perform any sensitivity analyses as the individual study results were not combined in a meta‐analysis.

**Figure 14:**
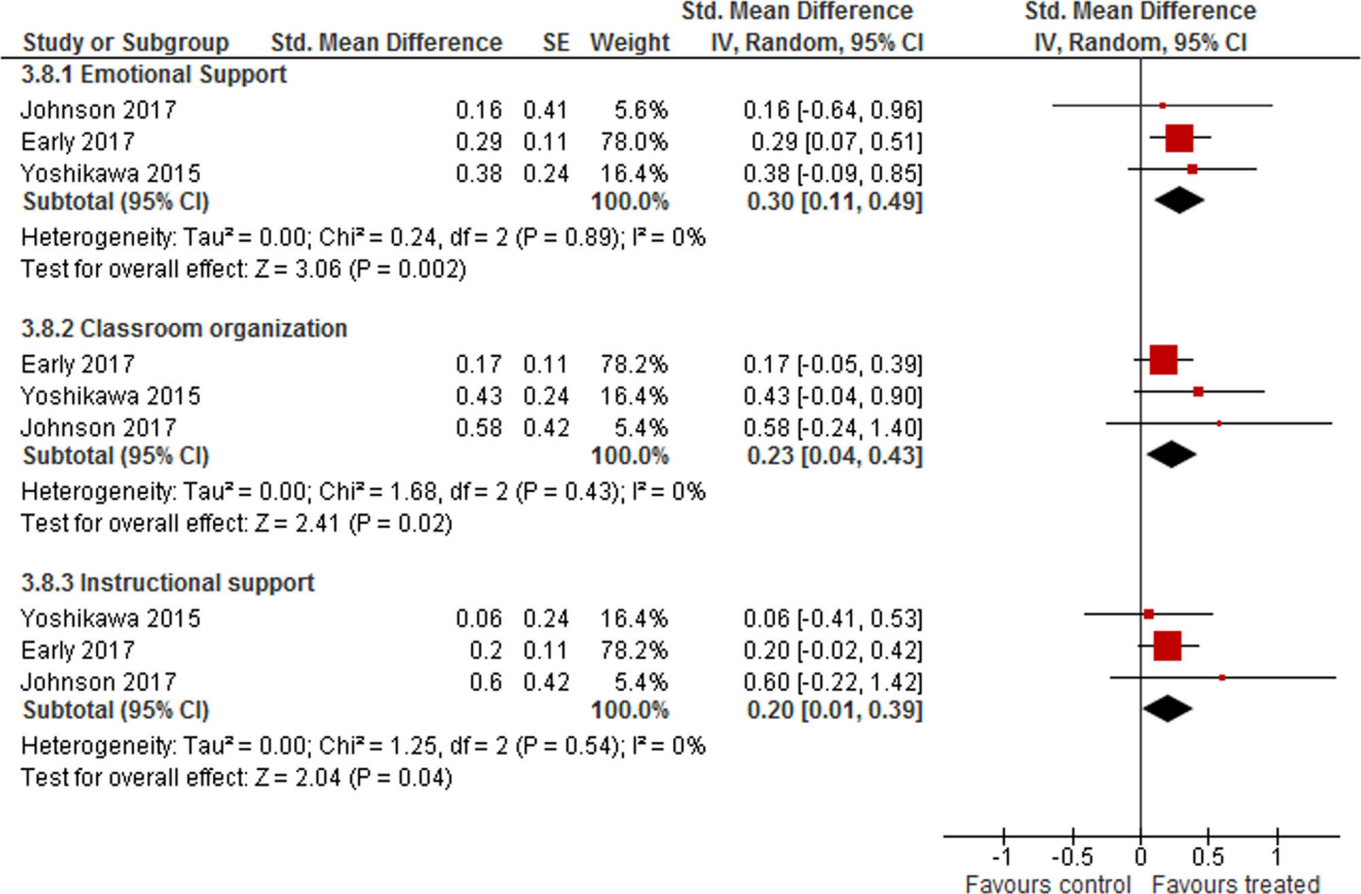
Summary CLASS

Three studies reported on three summary CLASS measures (Emotional support, Instructional support and Classroom organisation). They were combined in a meta‐analysis as displayed in Figure 14. The meta‐analysis of the studies showed no evidence of statistical heterogeneity with an *I* ^2^ value of 0% and the estimated *τ* ^2^ is 0.00. The weighted average effects are all positive and statistically significant. The weighted average SMD of Emotional support is 0.30 (95% CI [0.11, 0.49]); for Classroom organisation it is 0.23 (95% CI [0.04, 0.43]) and for Instructional support it is 0.20 (95% CI [0.01, 0.39]). However, given there are very few studies, some caution is needed about the conclusion of no significant heterogeneity of effects from PD on these CLASS summary outcomes.

A sensitivity analysis was undertaken adjusting for clustering using an ICC of 0.19 for Emotional support, 0.21 for Classroom organisation and 0.35 for Instructional support; the values reported in the study by Early et al. (2017). Note that the analysis in Early took into account clustering; thus in the sensitivity analysis the results reported in that study was not further adjusted for clustering. The resulting forest plot (Figure G7 in Appendix G) show that the overall results of Emotional support and Classroom instruction do not change; whereas the weighted average of Instructional support is still positive but loses statistical significance.

There were an insufficient number of studies to perform sensitivity analyses of methodological quality.

**Figure 15:**
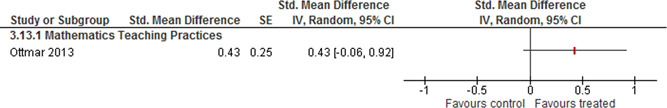
Other teacher outcomes

Finally, one study reported results on mathematic teaching practices as displayed in Figure 15. The effect is positive although not statistically significant.

##### 6.3.3.5 Publication Bias

We assessed the possibility of publication bias visually by examining funnel plots. Only the analysis of student academic achievement in the language and literacy development topic area was examined, as there were an insufficient number of studies in any other analysis. The funnel plot is displayed in Appendix G. There are too few studies to assess whether the funnel plot is symmetric. There is, however, no striking asymmetry visible in the funnel plot.

## 7 DISCUSSION

### 7.1 Summary of main results

A moderate body of experimental evidence exists in relation to the effect of PD in the topic area of education; similar evidence does not appear to exist in the topic areas of social welfare and crime and justice.

#### 7.1.1 Social and emotional development

Four studies could be combined in a meta‐analysis of student academic outcomes. There seems to only a very small and statistically nonsignificant effect on student academic outcomes. The effects were measured by SMDs. The weighted average effect was 0.05 (95% CI [−0.07, 0.16]) and not statistically significant. However, given the relatively few studies and that there is some heterogeneity between them, some caution is needed in making an assumption that there is no effect from PD on student academic outcomes.

Two studies reported outcomes on student social competences and another two studies reported outcomes on student's socioemotional skills. The meta‐analyses of these two outcomes showed no evidence of statistical heterogeneity. Both weighted average effect sizes favoured the treated group. The weighted average SMD of student social competences is 0.13 (95% CI [0.03, 0.24]) and 0.22 (95% CI [0.08, 0.37]) for student's socioemotional skills. However, given the very low number of studies, some caution is needed in assuming that there is a single true effect from PD on either student social competences or socioemotional skills.

Three studies reported outcomes on various other student measures that were too different to be combined.

Two studies could be combined in a meta‐analysis on three subscales of CLASS (Positive climate, Negative climate and Behavioural management). The weighted average effects are all positive but only Positive climate is statistically significant. The weighted average SMD of Positive climate is 0.61 (95% CI [0.08, 1.14]); for Negative climate it is 0.18 (95% CI [−0.73, 1.08]) and for Behaviour management it is 0.30 (95% CI [−0.14, 0.73]). Given there are only two studies reporting these teacher outcomes and there is some degree of heterogeneity in all analyses, some caution is needed in making an assumption that there is (or is not) a single true effect from PD on any of these teacher outcomes.

One of the studies in addition reported on the CLASS subscale Teacher sensitivity and one other study reported summary measures of CLASS.

In summary, at most the results from four individual studies could be combined in a single meta‐analysis. The results of the meta‐analyses should therefore be interpreted with great caution due to the very limited number of studies and selection of measures especially on teacher outcomes.

In short, therefore, the result of the analysis on social and emotional development is that there is currently insufficient evidence for conclusions to be drawn. The small number of available studies reporting similar outcomes precludes any conclusions concerning effectiveness or ineffectiveness of PD in the social and emotional development area.

#### 7.1.2 Language and literacy development

Thirteen studies could be combined in a meta‐analysis of student academic outcomes. There seems to be no effect on student academic outcomes. The weighted average effect was 0.04 (95% CI [−0.01, 0.10]).

Sensitivity analyses were undertaken adjusting for clustering and evaluating whether the pooled effect size was robust across study design and components of methodological quality. The result was somewhat sensitive due to the removal of studies with scores of 4 on the blinding component; the weighted average SMD became larger and statistically significant when studies with blinding scores of 4 where removed. Note, however, that only four studies contributed to the average. Otherwise, the overall result did not change. This suggests that, although the overall effect on student academic outcomes is positive, it is very small and not statistically significant.

There seem to be a positive effect on teacher outcomes measured by ELLCO, although only three studies reported the total ELLCO scores. The weighted average SMD was 0.45 (95% CI [0.16, 0.74]) and there was a small amount of heterogeneity between the studies. There was no need for cluster correction in any of the studies. One study further reported results from two ELLCO subscales and one study reported results on mathematics teaching practices.

There also seems to be a positive effect on teacher outcomes measured by the three summary CLASS measures (Emotional support, Instructional support and Classroom organisation), although only three studies reported these measures. The weighted average effects were all positive and statistically significant and there was no evidence of heterogeneity between the studies. The weighted average SMD of Emotional support was 0.30 (95% CI [0.11, 0.49]); for Classroom organisation it was 0.23 (95% CI [0.04, 0.43]) and for Instructional support it was 0.20 (95% CI [0.01, 0.39]). The weighted average of Instructional support lost statistical significance in the sensitivity analysis of cluster correction.

In short, the result of the analysis on language and literacy development is that there seems to be no effect on student academic outcomes.

Given there are only at most three studies reporting the same teacher outcome, measured either by the full ELLCO or summary CLASS measures respectively, we cannot conclude on the effect from language and literacy PD on any teacher outcomes.

### 7.2 Overall completeness and applicability of evidence

#### 7.2.1 Social and emotional development

A total of 10 trials reported in 12 papers analysed PD on social and emotional development.

The majority of studies did not report on student academic outcomes and in general the outcomes reported, student as well as teacher outcomes, were too different to be combined. If all the nine studies had provided an effect estimate of both students and teachers using common standardised measures, the number of useable studies in a single meta‐analysis would have been larger which again would have provided a more robust literature on which to base conclusions.

Five studies were undertaken in the United States, with only one study undertaken in each of the following countries: Denmark, Ireland, the Netherlands, New‐Zealand and Portugal. The study from Portugal could, however, not be used in the meta‐analysis as there was uncertainty on how the reported standard deviations were calculated.

The dominance of the United States as the main country in which PD interventions meeting our criteria have been evaluated using rigorous methods and within our specific parameters clearly limits the generalisability of the findings.

Moreover, the limited number of studies prevented an analysis of specific PD‐approaches across cultures, across professions/service‐deliverer types, across organisations, across service‐receiver types, and so forth.

All outcome measurements were performed relatively close to the end of the interventions. The longer‐term effects of PD‐approaches on social and emotional development were therefore not possible to analyse.

It was not possible to assess publication bias due to the limited number of studies.

#### 7.2.2 Language and literacy development

In this review in total 17 studies (evaluating 16 trials) were used in the meta‐analyses of language and literacy development. This number is very low compared to the larger number of studies (38 evaluating 33 trials) meeting the inclusion criteria. The reduction was caused mainly by the studies being rated to have too high risk of bias. In total 16 studies were judged to have a very high risk of bias (5 on the scale) and, in accordance with the protocol, we excluded these from the meta‐analysis on the basis that they would be more likely to mislead than inform. A further two studies could not be included in the meta‐analysis as there was uncertainty on how the reported standard deviations were calculated^9^ and one study did not report results in a format that could be used in the meta‐analysis.

If all studies had provided an effect estimate with lower risk of bias, the final list of useable studies in the meta‐analysis would have been larger which again would have provided a more robust literature on which to base conclusions.

Twenty‐eight of the 33 trials were undertaken in the United States; and one trial was undertaken in each of the following countries: Australia, Chile and Germany and two trials were undertaken in the UK. The 16 trials used in the meta‐analysis covered the United States, Chile and Germany. The geographical coverage thus became even narrower as the studies from Australia and the UK could not be used in the meta‐analysis. This is a clear limitation of the review.

Moreover, the limited number of studies that could be used in a single meta‐analysis prevented an analysis of specific PD‐approaches across cultures, across professions/service‐deliverer types, across organisations, across service‐receiver types, and so forth.

All outcome measurements were performed relatively close to the end of the interventions. The longer‐term effects of PD‐approaches on language and literacy development were, therefore, not possible to analyse.

We found no strong indication of publication bias.

### 7.3 Quality of the evidence

The majority of studies used randomised designs. Overall the risk of bias in the included studies of language and literacy development was high.

Among the 12 studies (10 trials) analysing PD on social and emotional development, none were judged to be at very high risk of bias.

Among the 38 studies (33 trials) analysing PD on language and literacy development, 16 studies were judged to be at very high risk of bias.

The risk of bias was examined using a tool for assessing risk of bias incorporating nonrandomised studies. We attempted to enhance the quality of the evidence in this review by excluding studies judged to be at very high risk of bias using this tool. We believe this process excluded those studies that are more likely to mislead than inform.

Furthermore, where possible, we performed a number of sensitivity analyses for each outcome to check whether the obtained results are robust across methodological quality and to correcting for cluster randomisation if needed.

One teacher outcome in the language and literacy area (the summary CLASS measure Instructional support) lost statistical significance when correcting for cluster randomisation. Taking clustering into account suggests that, we need to be somewhat cautious in attributing a treatment effect on this outcome as this could result from chance depending on the amount of clustering (i.e., the true size of ICC). Otherwise, none of the conclusions in neither the social and emotional development area nor the language and literacy area changed when correcting for clustering.

To check the robustness across study design and components of methodological quality, we removed the one nonrandomised study and studies with risk of bias score of 4 or Unclear on the Blinding, Incomplete outcome data, Selective reporting and Other bias components of the risk of bias checklists, respectively in the analysis of student academic outcomes in the language and literacy area. The student academic weighted average SMD became larger and statistically significant when studies with blinding scores of 4 (none were rated Unclear) where removed. Note, however, that only four studies contributed to the average. Otherwise, the overall conclusion did not change.

There were too few studies to perform study design and methodological sensitivity analyses for the remaining outcomes.

There was overall good consistency in the direction of effects on student outcomes and only some heterogeneity in one of the analyses (student academic scores in the social and emotional development area). The single study effects favoured the treated with only a few exceptions and all combined effects favoured the treated, although not all were statistically significant.

There was overall good consistency in the direction of effects on teacher outcomes and only some heterogeneity in one of the analyses (the CLASS subscales Positive climate, Negative climate and Behavioural management in the social and emotional development area). The single study effects favoured the treated with only a few exceptions and all combined effects favoured the treated, although a few were not were statistically significant.

### 7.4 Limitations and potential biases in the review process

We believe that there are no potential biases in the review process as screening at all stages was completed independently by two reviewers, and agreement to include or exclude was high; where there was disagreement, agreement was achieved through discussion. Referring back to Table 4 we can see that 112 studies were excluded at third stage (full text) screening by way of quality assurance—46% of the studies excluded at third stage were excluded for a reason pertaining to the outcome measures reported: all reviewers agreed on these exclusions. Data extraction for the 51 remaining studies that were included was independent. Agreement was very high; any differences were resolved by discussion and with occasional reference to a third reviewer.

Data extraction for the risk of bias assessment and extraction of numerical data were undertaken by reviewers working in pairs. Agreement was initially quite good, and full consensus was achieved through discussion.

We assessed the possibility of publication bias visually by examining funnel plots were possible. Only the analysis of student academic achievement in the language and literacy development topic area was examined. There was no striking asymmetry visible in the funnel plot.

For the remaining outcomes, we were unable to comment on the possibility of publication bias because there were insufficient studies included in the meta‐analysis for the construction of funnel plots. Thus, it may be possible there are some missing studies.

### 7.5 Agreements and disagreements with other studies or reviews

We identified two SRs in the area of professional development in professionals working with children and adolescent that compare to our SR (Kraft et al., 2018; Markussen‐Brown et al., 2017).

Markussen‐Brown et al. (2017) conducted a SR and meta‐analysis in the specific area of professional development in professionals working with children's early language and literacy development. Participants had to be in‐service educators or home‐based child‐care providers working with 3–6‐year‐old children United States or Canada. Twenty‐five studies (containing 33 trials altogether) were included; 13 of which were included in our review too. However, seven of these 13 studies were excluded from the meta‐analyses in our review due to too high risk of bias. Markussen‐Brown et al. (2017) conducted meta‐analyses to evaluate the effects of language‐ and literacy‐focused PD on the teacher outcomes process quality, structural quality and educator knowledge as primary outcomes. Furthermore, three child outcomes were analysed: receptive vocabulary, phonological awareness and alphabet knowledge.

The overall pooled SMD, using 30 effect estimates, for process quality was 0.59 (95% CI [0.41, 0.76]); for structural quality it was 1.07 (95% CI [0.69, 1.45]) using 16 effect estimates and finally for educator knowledge it was 0.12 (95% CI [−0.04, 0.30]) using 11 effect estimates.

Fewer studies provided results for children. An overall SMD of 0.21 (95% CI [−0.01, 0.43]) using five effect estimates was found for receptive vocabulary; for phonological awareness it was 0.30 (95% CI [0.13, 0.48]) using nine effect estimates and finally, a pooled SMD of 0.12 (95% CI [0.05, 0.19]) using 11 effect estimates was found for alphabet knowledge.

Concerning student academic outcomes we combined average effect estimates from 13 studies and found a pooled SMD of 0.04 (95% CI [−0.01, 0.10]) which is not comparable to any of the results reported in Markussen‐Brown et al. (2017) on student academic outcomes.

The results concerning teacher outcomes are not comparable to ours either. We only found at most three studies reporting similar teacher outcomes in the language and literacy area, precluding any conclusions concerning effectiveness or ineffectiveness of PD in this topic area. A likely explanation to this inconsistency is that Markussen‐Brown et al. (2017) did not exclude from their meta‐analysis studies with too high risk of bias (we excluded seven of the studies we have in common) and used all available measures, although not self‐reported measures. Furthermore, the included studies in the Markussen‐Brown et al. (2017) review had to be published in peer‐reviewed journals making the results susceptible to publication bias.

Kraft et al. (2018) conducted a SR and meta‐analysis in the specific area of teacher coaching programmes on classroom instruction and student achievement. Participants had to be in‐service teachers working with students in early childhood to 12th grade in United States or “other developed countries”. Sixty studies were included; 16 of which were included in our review too. However, five of these 16 studies were excluded from the meta‐analyses in our review due to too high risk of bias. Kraft et al. (2018) conducted meta‐analyses to evaluate the effects of teacher coaching programmes on teacher instruction and student achievement. Robust variance estimation methods were used to account for the nonindependence of multiple effect sizes from the studies.

The overall pooled SMD, using 186 effect estimates from 43 studies for teacher instruction was 0.49 (95% CI [0.38, 0.60]). Fewer studies provided results for children. An overall SMD of 0.18 (95% CI [0.11, 0.25]) using 113 effect estimates from 31 studies was found.

Concerning student academic outcomes we found a pooled SMD of 0.04 (95% CI [−0.01, 0.10]) in the language and literacy area and a pooled SMD of 0.05 (95% CI [−0.07, 0.16]) in the social and emotional development area, none of which are comparable to the result reported in Kraft et al. (2018) on student academic outcomes.

The results concerning their teacher outcome is not comparable to ours either. We only found at most three studies reporting similar teacher outcomes, precluding any conclusions concerning effectiveness or ineffectiveness of PD. A likely explanation to this inconsistency is that Kraft et al. (2018) did not exclude from their meta‐analyses studies with too high risk of bias (we excluded five of the studies we have in common) and used all available measures (although it should be rated by an outside observer) in the meta‐analysis.

## 8 AUTHOR'S CONCLUSION

### 8.1 Implications for practice

There is a political push to promote the use of evidence‐informed interventions, that is, ones that have been proven to be effective according to the highest possible levels of effectiveness research standards. This is true of interventions in the broader social sector as well as in the narrower sector of schooling and education. A moderate body of experimental evidence exists in relation to the effect of PD in the topic area of education; similar evidence does not appear to exist in the topic areas of social welfare and crime and justice.

The small number of available studies reporting similar teacher outcomes precludes any conclusions concerning effectiveness or ineffectiveness of PD on teachers. Professional development may be costly and the available evidence points to no effect of CPD in comparison to “business‐as‐usual” professional development on student academic outcomes; the weighted average effect is very small and not statistically significant. However, it should be noted that included studies measured outcomes directly after the end of the interventions. The longer‐term effects on teacher and student outcomes are therefore not known. Because teachers may become better at implementing new practices with repetition over time, as improved teacher practices affect new cohorts of children and students, the longer‐term effects could be different from the short‐term effects.

The vast majority of studies were undertaken in the United States. The dominance of the United States as the main country in which PD interventions meeting our criteria have been evaluated using rigorous methods and within our specific parameters clearly limits the generalisability of the findings. Research which demonstrates (some degree of) effectiveness in the setting where the intervention has been developed, tested and evaluated cannot necessarily be generalised to another context. According to Gardner et al. (2016) there is a growing literature on the topic of transferability of effective interventions from one cultural and structural context to another. Cultural norms, family and societal values, educational structures, and political priorities will all influence the acceptability and effectiveness of attempts to “re‐plant” specific interventions in a context other than the one in which they were originally “grown”. Such differences are important, when considering the relevance and potential for transferring interventions from one setting to another; yet it is also important to look for commonalities, which may indeed facilitate the process (Gardner et al., 2016).

An objective of the review was to examine and compare the effect sizes of specific PD‐approaches across cultures, across professions/service‐deliverer types, across organisations, across service‐receiver types, and so forth. The limited number of studies, however, prevented such an analysis.

### 8.2 Implication for research

The vast majority of studies were undertaken in the United States and none of the studies were considered to be of overall high quality in our risk of bias assessment. The process of excluding studies with too high risk of bias from the meta‐analysis applied in this review left us with only 17 of a total of 38 studies to synthesise in the language and literacy area.

This is a finding in its own right, entailing important information on the degree of confidence to place on the reported gains from PD in the language and literacy area.

Given the limited number of rigorous studies available at this time from countries other than the United States, it would be natural to consider conducting a large RCT (or a series of large RCTs) evaluating the effectiveness of a PD intervention in the topic area of social and emotional development or language/literacy development in countries outside of the United States. Specific attention would then have to be paid to stringency in terms of conducting a well‐designed RCT with low risk of bias as well as ensuring that the sample sizes are large enough to enable sufficient power. Moreover, consideration should be made to which types of outcomes are most relevant. Student outcomes should be the primary outcomes (e.g., academic achievement, socioemotional and behavioural outcomes). The reason for this is that the ultimate goal of any teacher PD ought to be to have a positive impact on students' well‐being and academic progress in school. Teacher outcomes would then be considered as secondary outcomes in the sense that they are important, but mainly as intermediate factors working toward the ultimate goal of improving student outcomes. In this way, such adapted trials in other countries than the United States would have the potential of making useful contributions to the PD effectiveness literature if due consideration is made to the strengths and weaknesses of the studies found in this review. The trial should be designed, conducted and reported according to methodological criteria for rigour in respect of internal and external validity in order to achieve robust results regarding both the short‐term and the longer‐term effects.

## CHANGES FROM THE PROTOCOL

Inclusion and exclusion criteria specifically relating to outcomes (experimenter designed and self‐reported) were added as a variation to the protocol at the third stage of screening. Studies were only included if they included at least one valid and reliable outcome that had been standardised on a different population and was “objective”, that is, not “experimenter‐designed” and not self‐reported.

*Experimenter designed outcome measures* that have been designed by the author(s) have typically been developed for the specific study and have not been validated or standardised with another sample. In some cases, the instruments have been pilot‐tested, but this is not adequate in terms of being able to have full confidence in the quality and validity of the outcome measure. In other cases, the authors have combined existing instruments with experimenter designed items and can thus be thought of as *experimenter adjusted outcome measures*. The use of *self‐reported outcome measures* is also quite widespread in many of the studies found in the early screening for this review—typically alongside other more objective and reliable outcome measures. The problem here is of course—by definition—risk of self‐reporting bias—typically in the direction of over‐estimating a possible effect of the intervention. We therefore excluded studies that relied exclusively on self‐reported outcome measures, which had not been based on validated assessment tools.

## METHODS NOT IMPLEMENTED

The limited number of studies prevented an analysis of specific PD‐approaches across cultures, across professions/service‐deliverer types, across organisations, across service receiver types, and so forth.

## ROLES AND RESPONSIBILITIES

Below is listed who is responsible for the following areas:

- Content: Carole Torgerson, Louise Gascoine, Chantal Nielsen, Jens Dietrichson
- Systematic review methods: Trine Filges, Carole Torgerson, Jens Dietrichson
- Statistical analysis: Trine Filges, Jens Dietrichson
- Information retrieval: Bjørn Viinholt

## SOURCES OF SUPPORT

SFI/VIVE Campbell, Durham University and Trygfonden

## DECLARATIONS OF INTEREST

The authors have no vested interest in the outcomes of this review, nor any incentive to represent findings in a biased manner.

## PLANS FOR UPDATING THE REVIEW

We plan to update the review with a frequency of 2 years if funding is available. Trine Filges will be responsible.

## Supporting information

Supporting information

## Footnotes

1 TAs are not included within this, although we acknowledge the relevance of work that they do in terms of welfare. In the UK context, the role of TA is not a degree level profession although it is likely that there are many TA's working who have a degree.

2 Dunst et al. (2015) was located and retrieved in our systematic searches.

3 Since January 2018: The Danish Centre for Social Science Research (VIVE).

4 The upper limit of the intracluster correlation (ρ) of 0.22 is based on the analysis in Stockford (2009).

5 Special caution was intended to be taken concerning studies using RD to estimate a local average treatment effect (LATE). These were to be included, but subject to a separate analysis depending on the comparability between the LATEs and the effects from other studies. We intended to check the sensitivity of our results to the inclusion of RD studies. In addition, we intended to discuss the limitation in generalisation of results obtained from these types of studies. However, no studies employing a RD were included in the review.

6 To check robustness we used another two methods of estimating between‐study variance (profile likelihood and restricted maximum likelihood) and there were no appreciable changes in results.

7 To check robustness we used another two methods of estimating between‐study variance (profile likelihood and restricted maximum likelihood) and there were no appreciable changes in results.

8 To check robustness we used another two methods of estimating between‐study variance (profile likelihood and restricted maximum likelihood) and there were no appreciable changes in results.

9 E‐mails were sent to the authors to clarify the uncertainty but we have received no answers.
