# Supplementary material for: Effectiveness of continuing professional development training of welfare professionals on outcomes for children and young people: A systematic review
Source: Campbell Syst Rev. 2019 Nov 7;15(4):e1060. doi: 10.1002/cl2.1060 (PMC8533679; doi:10.1002/cl2.1060)

# Appendices

## Appendix A: Justification of exclusion of studies using an instrumental variable (IV) approach

Studies using instrument variables (IV) for causal inference will not be included as the interpretation of IV estimates is challenging. IV only provides an estimate for a specific group namely, people whose behaviour change due to changes in the particular instrument used. It is not informative about effects on never-takers and always-takers because the instrument does not affect their treatment status. The estimated effect is thus applicable only to the subpopulation whose treatment status is affected by the instrument. As a consequence, the effects differ for different IVs and care has to be taken as to whether they provide useful information. The effect is interesting when the instrument it is based on is interesting in the sense that it corresponds to a policy instrument of interest. Further, if those that are affected by the instrument are not affected in the same way the IV estimate is an average of the impacts of changing treatment status in both directions, and cannot be interpreted as a treatment effect. To turn the IV estimate into a LATE requires a monotonicity assumption. The movements induced by the instrument go in one direction only, from no treatment to treatment. The IV estimate, interpreted as a LATE, is only applicable to the complier population, those that are affected by the instrument in the ‘right way’. It is not possible to characterise the complier population as an observation’s subpopulation cannot be determined and defiers do not exist by assumption.

In the binary-treatment–binary-instrument context, the IV estimate can, given monotonicity, be interpreted as a LATE; i.e. the average treatment effect for the subpopulation of compliers. If treatment or instruments are not binary, interpretation becomes more complicated. In the binary-treatment–multivalued-instrument (ordered to take values from 0 to *J*) context, the IV estimate, given monotonicity, is a weighted average of pairwise LATE parameters (comparing subgroup *j* with subgroup *j*−1). The IV estimate can thus be interpreted as the weighted average of average treatment effects in each of the *J* subgroups of compliers. In the multivalued-treatment (ordered to take values from 0 to *T*) – multivalued-instrument (ordered to take values from 0 to *J*) context, the IV estimate for *each pair of instrument values*, given monotonicity, is a weighted average of the effects from going from *t*-1 to *t* for persons induced by the change in the value of the instrument to move from any level below *t* to the level *t* or any level above. Persons can be counted multiple times in forming the weights.

*Bibliography*

Angrist, J.D., & Pischke, J.S. (2009*). Mostly Harmless Econometrics: An Empiricist’s Companion.* Princeton, NJ: Princeton University Press.

Heckman, J.J. & Urzúa, S. (2010). Comparing IV with structural models: What simple IV can and cannot identify. *Journal of Econometrics, 156*, 27-37.

Heckman, J.J., Urzúa, S. & Vytlacil, E. (2006). Understanding instrumental variables in models with essential heterogeneity. *The Review of Economics and Statistics, 88*(3), 389-432.

## Appendix B: Results of initial scoping search

Al Otaiba, S., Connor, C. M., Folsom, J. S., Greulich, L., Meadows, J., & Li, Z. (2011). Assessment Data-Informed Guidance to Individualize Kindergarten Reading Instruction: Findings from a Cluster-Randomized Control Field Trial. *Elementary School Journal, 111*(4), 535-560. doi: 10.1086/659031

Álvarez, Pedro, De La Fuente, Emilia I, Perales, F Javier, & García, Juan. (2002). Analysis of a quasi-experimental design based on environmental problem solving for the initial training of future teachers of environmental education. *The Journal of Environmental Education, 33*(2), 19-21.

Antoniou, P., & Kyriakides, L. (2011). The impact of a dynamic approach to professional development on teacher instruction and student learning: results from an experimental study. *School Effectiveness and School Improvement, 22*(3), 291-311. doi: 10.1080/09243453.2011.577078

Arbolino, L. A., & Learning, U. S. (2007). *In-service training on child abuse for classroom teachers: What is the effectiveness of mandated training? (67), ProQuest Information & Retrieved from http://search.ebscohost.com/login.aspx?direct=true&db=psyh&AN=-034&site=ehost-live Available from EBSCOhost psyh database*.

Aronin, S. A., & Learning, U. S. (2010). *Integrating universal design for learning through content video with preservice teachers*.

Barr, D. J., Boulay, B., Selman, R. L., McCormick, R., Lowenstein, E., Gamse, B., . . . Learning, U. S. (2013). *A randomized controlled trial of professional development for interdisciplinary civic education: Impacts on humanities teachers and their students* (Vol. 117).

Butler, Michelyn Cynthia. (2012). *Implementation of Evidence-Based Book-Reading Strategies by Head Start Teachers: Benefits of Professional Development and Effect on Children's Literacy Outcomes*: ERIC.

Campanaro, M. (2009). *The effects of a professional development training protocol on teacher implementation of comprehension strategy instruction*.

Davis, C. A., & Learning, U. S. (2005). *Effects of in-service training on teachers' knowledge and practices regarding identifying and making a focus of concern students exhibiting internalizing problems*.

Ellis, H. A. (2014). Effects of a Crisis Intervention Team (CIT) training program upon police officers before and after Crisis Intervention Team training. *Arch Psychiatr Nurs, 28*(1), 10-16. doi: 10.1016/j.apnu.2013.10.003

Ellis-Mills, P. E., & Learning, U. S. (1996). *Benefits of training sixth-grade Bahamian teachers to use conflict resolution techniques in the classroom*.

Filer, A. F., & Learning, U. S. (2016). *Investigating the use of traditional and online instruction for teachers of children with Autism Spectrum Disorder: A case for blending training models*.

Foltz, S. P. (2000). *The effect of police academy training on self-esteem and locus of control in law enforcement recruits*.

Frank, G. (2007). *The effect of elementary teachers' professional development activities intended to increase the number of migrant student nominations for gifted and talented programs*.

Lane, Cristianne, Prokop, Mary Jo Surges, Johnson, Evelyn, Podhajski, Blanche, & Nathan, Jane. (2013). Promoting early literacy through the professional development of preschool teachers. *Early Years, 34*(1), 67-80. doi: 10.1080/09575146.2013.827157

Powell, Ed. (2005). Conceptualising and facilitating active learning: teachers’ video‐stimulated reflective dialogues. *Reflective Practice, 6*(3), 407-418. doi: 10.1080/14623940500220202

Roeser, R. W., Schonert-Reichl, K. A., Jha, A., Cullen, M., Wallace, L., Wilensky, R., . . . Harrison, J. (2013). Mindfulness Training and Reductions in Teacher Stress and Burnout: Results From Two Randomized, Waitlist-Control Field Trials. *JOURNAL OF EDUCATIONAL PSYCHOLOGY, 105*(3), 787-804. doi: 10.1037/a0032093

Skoretz, Y. M., & Learning, U. S. (2011). *A study of the impact of a school-based, job-embedded professional development program on elementary and middle school teacher efficacy for technology integration*.

Sluijsmans, Dominique M. A., Brand-Gruwel, Saskia, & van Merriënboer, Jeroen J. G. (2002). Peer Assessment Training in Teacher Education: Effects on performance and perceptions. *Assessment & Evaluation in Higher Education, 27*(5), 443-454. doi: 10.1080/0260293022000009311

Tao, X., Chongde, L., & Jiliang, S. (1999). Effect of cognitive self-instruction training on the improvement of teachers' teaching-regulated ability. *Psychological Science China, 22*(1 SRC - GoogleScholar), 5-9.

Wasik, B. A., & Hindman, A. H. (2011). Improving Vocabulary and Pre-Literacy Skills of At-Risk Preschoolers Through Teacher Professional Development. *JOURNAL OF EDUCATIONAL PSYCHOLOGY, 103*(2), 455-469. doi: 10.1037/a0023067

## Appendix C: Final search strategies and tracking of records from each database searched

### C1: Final search strategies

#### Web of Science (Social Science Citation Index & Science Citation Index)

1997-2017. Search performed 1/5-2017.

| **Search** | **Terms** | **Results** |
| --- | --- | --- |
| S7 | #6 AND #5 AND #2 AND #1  *Indexes=SCI-EXPANDED, SSCI Timespan=1997-2017* | [544](http://apps.webofknowledge.com/summary.do?product=WOS&doc=1&qid=30&SID=P1Fe2Sy8CcLGZFmqoOM&search_mode=CombineSearches&update_back2search_link_param=yes) |
| S6 | #4 OR #3  *Indexes=SCI-EXPANDED, SSCI Timespan=1997-2017* | [5,490,876](http://apps.webofknowledge.com/summary.do?product=WOS&doc=1&qid=29&SID=P1Fe2Sy8CcLGZFmqoOM&search_mode=CombineSearches&update_back2search_link_param=yes) |
| S5 | TI=(teacher* OR pedagogue* OR school counsellor* OR social worker* OR police* OR psychologist* OR probation officer* OR family support OR support worker*)  *Indexes=SCI-EXPANDED, SSCI Timespan=1997-2017* | [46,106](http://apps.webofknowledge.com/summary.do?product=WOS&doc=1&qid=28&SID=P1Fe2Sy8CcLGZFmqoOM&search_mode=AdvancedSearch&update_back2search_link_param=yes) |
| S4 | TS=(profess* OR develop* OR learn* OR train* OR supervis* OR feedback OR team work OR education*)  *Indexes=SCI-EXPANDED, SSCI Timespan=1997-2017* | [5,490,876](http://apps.webofknowledge.com/summary.do?product=WOS&doc=1&qid=25&SID=P1Fe2Sy8CcLGZFmqoOM&search_mode=AdvancedSearch&update_back2search_link_param=yes) |
| S3 | TI=(profess* OR develop* OR learn* OR train* OR supervis* OR feedback OR team work OR education*)  *Indexes=SCI-EXPANDED, SSCI Timespan=1997-2017* | [1,055,729](http://apps.webofknowledge.com/summary.do?product=WOS&doc=1&qid=12&SID=P1Fe2Sy8CcLGZFmqoOM&search_mode=AdvancedSearch&update_back2search_link_param=yes) |
| S2 | TI=(random* control* trial* OR rct* OR trial* OR review* OR intervent* OR meta analys*)  *Indexes=SCI-EXPANDED, SSCI Timespan=1997-2017* | [694,515](http://apps.webofknowledge.com/summary.do?product=WOS&doc=1&qid=19&SID=P1Fe2Sy8CcLGZFmqoOM&search_mode=AdvancedSearch&update_back2search_link_param=yes) |
| S1 | TS=(child* OR student* OR adolescent* OR teen* OR preschool*)  *Indexes=SCI-EXPANDED, SSCI Timespan=1997-2017* | [1,419,905](http://apps.webofknowledge.com/summary.do?product=WOS&doc=1&qid=8&SID=P1Fe2Sy8CcLGZFmqoOM&search_mode=AdvancedSearch&update_back2search_link_param=yes) |

The search was updated 27/11/2018. 644 new records were identified.

#### EBSCO databases (ERIC, Academic Search Premier, PsycINFO, Teacher Reference Center, SocIndex)

1997-2017. Search performed 17/4-2017

| **Search** | **Terms** | **Results** |
| --- | --- | --- |
| S6 | S1 AND S2 AND S3 AND S4 Limiters - Published Date: 19970101-20171231 | 3,139 |
| S5 | S1 AND S2 AND S3 AND S4 | 4,626 |
| S4 | SU (child* OR student* OR adolescent* OR teen* OR preschool*) | 2,981,315 |
| S3 | SU ((random* control* trial* OR rct* OR trial* OR review* OR intervent*) OR TI (random* control* trial* OR rct* OR trial* OR review* OR intervent*)) | 2,395,696 |
| S2 | SU ((continu* OR proffes* OR in service OR teacher*) AND (professional OR development OR learning OR training OR supervis* OR feedback OR team work OR education*)) | 1,101,911 |
| S1 | SU ((teacher* OR classroom* OR para* OR auxiliary) AND (teacher* OR assist* OR support* OR aid*) OR pedagogue* OR school counsellor* OR social worker* OR police* OR psychologist* OR probation officer* OR family support OR support worker*)) | 1,249,212 |

The individual results (after duplication) from each EBSCO-database are listed below:

- *Academic Search Premier* – 528 records.
- *ERIC* –1385 records.
- *PsycINFO* – 791 records.
- *SocIndex* –215 records.

*Teacher Reference Center* - 156 records.Search was updated 27/11/2018 with following new records from each database:

- *Academic Search Premier* – 188 new records.
- *ERIC* – 573 new records.
- *PsycINFO* – 70 new records.
- *SocIndex* – 30 new records.
- *Teacher Reference Center* – 60 new records.

#### ASSIA

1997-2017

Searches performed 4/5 2017

| **Search** | **Terms** | **Results** |
| --- | --- | --- |
| S6 | 1 AND 2 AND 3 AND 4 AND pd (19970101-20171231) | 236 |
| S5 | 1 AND 2 AND 3 AND 4 | 243 |
| S4 | su(((teacher* OR classroom* OR para* OR auxiliary (teacher* OR assist* OR support* OR aid*)) OR pedagogue* OR school counsellor* OR social worker* OR police* OR psychologist* OR probation officer* OR family support OR support worker*)) | 36, 690 |
| S3 | su(((continu* OR proffes* OR in service OR teacher* (professional OR development OR learning OR training OR supervis* OR feedback OR team work OR education*)))) | 9,347 |
| S2 | su(random* control* trial* OR rct* OR trial* OR review* OR intervent*) OR ti(random* control* trial* OR rct* OR trial* OR review* OR intervent*) | 88,826 |
| S1 | su((child* OR student* OR adolescent* OR teen* OR preschool*)) | 198,896 |

Search was updated 02/12/2018 which resulted in 129 new records.

### C2: Grey Literature Search Strategies

#### **Forskningsdatabasen (Danish National Research Database)**

Searches performed 01/05 - 2017

professional develop training effect – 75 hits.

professional develop training random – 7 hits.

professional develop train control – 29 hits.

continued professional develop effect – 60 hits.

continued professional develop random – 9 hits.

continued professional develop control – 46 hits.

continued professional train effect – 25 hits.

continued professional train random – 3 hits.

continued professional train control – 8 hits.

"CPD" effect – 13 hits.

"CPD" trial – 2 hits.

*References imported into Mendeley-database: 177*

*References imported to EPPI-reviewer after duplication in Mendeley: 171*

Search was updated 27/11/2018 which resulted in 51 new records.

#### **SwePub (Academic content from Swedish universities)**

Searches performed 01/05/2017

tit:(profess* develop* train*) – 14 hits.

tit:(profess* develop* effect*) – 3 hits.

tit:(profess* develop* control*) – 1 hits.

tit:(continu* profess* develop*) – 42 hits.

tit:(continu* profess* random*) -1 hits.

tit:(continu* profess* control*) -1 hits.

tit:(continu* profess* train*) – 1 hits.

Tit: (“CPD”) – 10 hits.

*References imported into Mendeley-database: 65*

*References imported to EPPI-reviewer after duplication in Mendeley: 65*

Search was updated 27/11/2018 which resulted in 20 new records.

#### **NORA (Norwegian Open Research Archive)**

Searches performed 01/05 - 2017

"professional development" AND effect -12 hits.

professional development effect – 88 hits.

professional development trial - 10 hits.

"professional development" AND training – 11 hits.

"continued professional development" – 4 hits.

continued professional training - 14 hits.

*References imported into Mendeley-database: 4*

*References imported to EPPI-reviewer after duplication in Mendeley: 4*

Search was updated 27/11/2018 which resulted in 0 new records.

#### **Social Care Online**

1997- 2017. Searches performed 01/05/2017

TITLE: continued professional OR professional*

AND

TITLE: train* OR learn* OR develop*

AND

AB: random* control* trial* OR rct* OR trial* OR review* OR intervent* OR effect*

*References imported to EPPI-reviewer after duplication in Mendeley: 129*

Search was updated 27/11/2018 which resulted in 4 new records.

#### **Google Scholar**

1997- 2017. Searches performed 02/05 - 2017

alleititel: professional effect develop OR learning OR training – 182 hits.

alleititel: continued professional develop OR learning OR training – 23 hits.

alleititel: professional intervention develop OR learning OR training – 81 hits.

alleititel: social work effect develop OR learning OR training – 10 hits.

alleititel: police effect develop OR learning OR training – 31 hits.

alleititel: continued professional develop OR learning OR training OR effect – 24 hits.

alleititel: continued professional development teacher OR psychologist OR police OR social OR worker – 5 hits.

*References imported into EPPI (after externally screening in Mendeley): 34*

Search was updated 27/11/2018 which resulted in 66 new records.

#### **U.S Clearinghouse for educational research & Danish Clearinghouse for educational research**

Searched performed 3/5/2017

We manually checked relevant repositories. Identified studies where already found in the database-search, so none were imported into EPPI.

Search was updated 27/11/2018 which resulted in 0 new records.

### C3: Flow chart


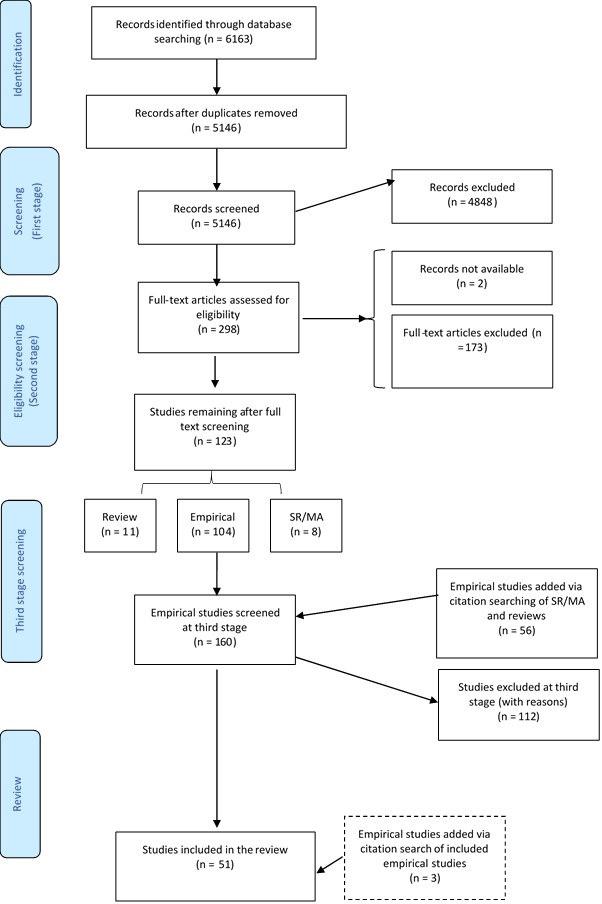


##

## Appendix D: Descriptive data extraction

### Social and emotional development

| Study |  | Fukkink & Tavecchio (2010) | Hickey et al., 2017 | Jennings et al., 2017 |
| --- | --- | --- | --- | --- |
| Participants | **Country** | Netherlands | Ireland | USA |
|  | **Professional** | Early childhood education and care teachers | 22 teachers | 224 teachers |
|  | **Other (e.g. students)** | Children in day care centres (but not focus of study) | 217 students |  |
| Eligibility | **Professional** | Not clear, but apparently teachers in 2 major day care providers SKON and KOREIN, p. 1654. | Teachers of either a Junior or Senior Infant class | "eligible teachers were identified that met the following criteria: taught in a classroom within the K–5 range of grade levels, taught general education (e.g., no art or physical education teachers), lead teacher in his or her classroom (e.g., no cotaught classrooms),2 taught the same students for the entirety of the school day, and had classrooms that were representative of the average classroom in this city (e.g., no single gender classrooms)." p. 1014 |
|  | **Other (e.g. students)** | Children in aforementioned day care centres | From the classes of the teachers who were in the study (approx. 12 children from each class). Excluded if consent not given. |  |
| Intervention | **Setting** | Day care centres | Primary schools | 36 schools - inner city public K–5 elementary schools in a high poverty region of New York City (the Bronx and Upper Manhattan). |
|  | **Dosage** | Typically four sessions of 10 min. videotaping, followed by session with discussion of video clips selected by trainer. | 1 day per month | 30 hours, 5 in person training days. In addition to in-person sessions, teachers were scheduled to receive a series of three one-on-one phone coaching calls |
|  | **Length** | Not clear, only mention of retention measurement 3 months after intervention. Mention of 3 measurements in all: pre, post, retention (only for intervention group). | 5 months | One school year |
|  | **Description** | "In the training, teachers were videotaped by their trainer for approximately 10 min while they are working with their groups. The trainer watched the video subsequently and selected a number of video fragments for review. In a next session, the trainer and the teacher subsequently engaged in a detailed discussion of these video clips. " (p. 1654) | IY TCM programme. Content of the intervention sessions, p. 178 "Teachers are encouraged to establish more positive relationships with pupils through the use of videotape modelling, role-plays and group discussions. They are also trained in specific skills, including developing clear classroom rules, using predictable schedules, giving praise and attention to pro-social behaviour, using encouragement and incentives, ignoring negative behaviour, using timeout and limit setting as non-aversive disciplining strategies, and facilitating child directed play and cooperative learning opportunities. The development of behaviour support plans for specific pupils is also supported during intervention delivery. During the month-long interval between each teacher training session, teachers received one telephone call so that they could discuss the implementation of the new classroom management strategies with one of the group facilitators. Teachers also kept a written diary and provided verbal feedback to (and received feedback from) the group and the facilitators on their progress at the next session. This provided teachers and facilitators with an opportunity to identify and address any difficulties they had when implementing the new strategies and behavioural support plans in their classrooms." | , p. 1014 "CARE is an innovative professional development program that introduces specific skills to help teachers manage stress and improve their teaching effectiveness. CARE combines emotion skills training with mindfulness-based stress reduction activities and provides teachers with opportunities to practice applying these skills in the classroom." |
| Control | **Description** | Control stated but no details, assume business as usual | Wait list control | Wait list control "118 teachers assigned to receive the CARE for Teachers program and 106 assigned to the wait-list control condition" (p.1015). |

| Study |  | Jensen et al. (2017) | Murray et al. (2014) | Raver et al. (2008) |
| --- | --- | --- | --- | --- |
| Participants | **Country** | Denmark | US | US |
|  | **Professional** | Trained pre-school staff (BA educated), also untrained preschool staff | Early elementary teachers of grades K-2 | Teachers and teachers’ aides in pre-school Head Start settings |
|  | **Other (e.g. students)** | Children in preschools | Early elementary students, grades K-2 | Children in the pre-school Head Start settings |
| Eligibility | **Professional** | Pre-school staff working in the recruited settings | Teachers working in selected schools. No additional details provided | Teachers and teachers’ aides working at eligible Head Start sites |
|  | **Other (e.g. students)** | 3-5-year-old children, focus on disadvantaged children | Children in selected schools. No additional details provided | Children in pre-school classes |
| Intervention | **Setting** | Pre-schools | Elementary schools in rural school districts selected due to below average school resources and difficulty recruiting and retaining highly qualified teachers. | Pre-schools |
|  | **Dosage** | 17 days training across the two years | 4-6 monthly full-day workshops (5 in the present study). Average number of workshop training hours across the 5 training days was 34. Teachers also had two brief consultation visits (avg. 44 min. total) | 5 training sessions each lasting 6 hours; coaching |
|  | **Length** | 2 years | 1 year | 1 school year (2 successive cohorts) |
|  | **Description** | VIDA intervention. Three key elements - knowledge (provided by training programme), reflection (about practices) and activities (via reflection leading to new activities). Intervention training focussed on evidence based - focus on improving children's skills by being 'responsive; to needs (and potential). Principals also offered 2-day course and workshop about facilitating organisational learning processes in the pre-schools. | IYT is a teacher-training program that is part of a comprehensive series of interventions including parent, child, and teacher training components that were designed to prevent and treat aggressive behaviour and conduct problems in young children aged 3-8 years. Its approach includes validated training methods such as video-modelling, behavioural rehearsal of key skills through numerous role-plays, classroom practice assignments, and teacher goal setting and self-monitoring. Workshops cover building positive relationships with students and parents, proactive classroom management strategies, effective use of incentives, “coaching” students’ social and emotional development, teaching calm-down and problem-solving and positive discipline techniques such as redirection, ignoring, and time out. Workshops are led by two trained co-leaders with approximately 12-15 teachers in each group | Treatment group teachers received: (1) training, (2) Mental Health Consultation (MHC) p. 7. A behaviourally- and evidence-based teacher-training package was selected and purchased, and a seasoned trainer with Licensed Clinical Social Worker (LCSW) qualifications delivered the 30 hours of teacher training over fall and winter, adapting the Incredible Years teacher-training module (Webster-Stratton, Reid, & Hammond, 2004). |
| Control | **Description** | Control condition not described, only "did not receive any training" (p. 26) | Business as usual, but not explicated. | p. 8 Business as usual plus teachers were given classroom support (teachers’ aides) to ensure similar ratios of staff to children across intervention and control conditions |

| Study |  | Reinke et al., 2016 | Rubie-Davies et al. (2015) | Seabra-Santos et al., 2018 |
| --- | --- | --- | --- | --- |
| Participants | **Country** | USA | New Zealand | Portugal |
|  | **Professional** | 105 teachers, recruited across 3 years (3 cohorts) | Elementary school teachers | 65 preschool teachers |
|  | **Other (e.g. students)** | 1817 kindergarten students | Elementary school students, grades 2-7 | 1030 preschool children |
| Eligibility | **Professional** | Kindergarten teachers | Teachers working in recruited schools | p. 3912 "Reasons for non-eligibility were having classes with fewer than 10 children (n = 9), participating in another study involving teacher training (n = 4), having had previous training in IY-TCM (n = 5), and working in a preschool with a number of classrooms that did not match any other of the contacted schools (n = 6)." |
|  | **Other (e.g. students)** | Kindergarten students | Students attending elementary schools at various socioeconomic levels in the city in which the study took place | Targeted disadvantaged children |
| Intervention | **Setting** | p. 1045: "nine urban schools in a school district in the Midwestern part of the USA. All the schools were implementing school-wide Positive Behavior Supports and Interventions (PBIS; Sugai et al. 2002), with high fidelity (scores above 90% as gathered and reported by independent state evaluators)." | 12 elementary schools from a suburban area of a city in Auckland, New Zealand | 52 public preschools |
|  | **Dosage** | 3 sets of 2 full day group trainings | 4 workshops: mid-March, end-March, mid-April, mid-May. After the workshops, researchers met with intervention teachers 3 further times to provide support and answer queries. | 36 hours in total - 6 monthly 6 hour workshops |
|  | **Length** | p. 1045 "October, December, and February. All trainings were cofacilitated by two doctoral-level IY TCM group leaders who were supervised by the program developer; one of these trainers also served as a coach." | 1 school year | 6 months (November - April) |
|  | **Description** | p. 1045 "IY TCM is a comprehensive curriculum for improving teacher classroom management skills. Specifically, teachers are trained to use proactive classroom management practices such as using behaviour-specific praise, building positive relationships with students, using pre-corrective statements, teaching classroom expectations, and using proximity to reduce disruptive behaviour. Much time during all training sessions is devoted to observing video vignettes of actual teacher interactions with students. The trainers prompt reflections with Socratic questioning about the videos (What is the student learning?, How would you respond in this situation?) and facilitate group discussion. These conversations spark role-plays to practice challenging interactions. In this way, teachers serve as models for others and/or get feedback about improving their skills." | Teacher expectation intervention. Through PD workshops, teachers were trained in the practices of teachers who have high expectations for all students. Three key areas: grouping and learning experiences, class climate, and goal setting. | IY-TCM, p. 3914 "Both between workshops and after the final workshop, there were four sessions of individual in-class support (Reinke et al. 2012). They consisted of co-leaders going to each class for one hour on average to assist teachers with the implementation of strategies learned in the workshops and to support them with problem-solving and goal-setting, or to make-up sessions for those who had missed a workshop. Between sessions 2–3 and 5–6, individualized coaching was done via email." |
| Control | **Description** | Wait list control (2 years after) | Regular professional development program of their school. They also attended 3 workshops related to this program (p. 78). Control group teachers were waitlisted for full participation in the following year. | Wait list control (the year after) |

| Study | Kolonne1 | Snyder et al., 2018 |
| --- | --- | --- |
| Participants | **Country** | USA |
|  | **Professional** | 36 preschool teachers |
|  | **Other (e.g. students)** | p. 217 ”Three target children were enrolled in 34 classrooms and two target children in two classrooms." |
| Eligibility | **Professional** | p. 216 "recruited from three school districts in three states (n = 13 from District 1 in State 1; n = 12 from District 2 in State 2; and n = 11 from District 3 in State 3)." |
|  | **Other (e.g. students)** | p. 217 "all target children had IEPs and were receiving preschool special education services under Section 619 of the Individuals With Disabilities Education Act." |
| Intervention | **Setting** | Preschools |
|  | **Dosage** | TfT workshops averaged 14.9 hr in length |
|  | **Length** | TFT on site coaching: 16 onsite workshops. TFT self-coaching: 16 email reminders, 16 weeks access to website. One school year |
|  | **Description** | Tools for Teachers (TfT) professional development. "teachers were assigned to one of three PD conditions at each site through a simple random assignment procedure: Tools for Teachers (TfT) workshops, accompanying implementation guides and materials, and onsite coaching; TfT workshops, accompanying implementation guides and materials, and self-coaching via a website; and BAU PD provided by the school district (see Figure 1)." p. 216 |
| Control | **Description** | Business as usual PD |

| Reinke 2018 | Murray et al., 2018 |
| --- | --- |
| Same trial and content as Reinke 2016 | Same trial and content as Murray 2014 |

### Language and literacy

| Study |  | Al Otaiba et al., 2011 | Allen et al. (2011) | Allen et al., 2015 |
| --- | --- | --- | --- | --- |
| Participants | **Country** | USA | US | USA |
|  | **Professional** | Teachers | Secondary school teachers | Middle or high school teachers |
|  | **Other (e.g. students)** | Kindergarten students | Secondary school students, aged 11-18 | Middle and high school students |
| Eligibility | **Professional** | Credentialed Kindergarten teachers from the schools | Teachers working in recruited schools and able to select a focal course for study (p. 2 in SOM, Supplementary Online Material) | Middle or high school teachers, agreeing to randomization, primary instructor for focal course, standardized exam to assess student learning |
|  | **Other (e.g. students)** | All students in the classrooms of the teachers | Students in selected courses, whose parents had provided written consent. Students also provided written consent | Middle and high school students who gave parental and own consent to participate |
| Intervention | **Setting** | A school district in a midsized city in northern Florida nominated 14 schools to be recruited; These schools served an economically and ethnically diverse range of students; six schools received Title I funding and four received Reading First funding. The percentage of students who were identified as limited English proficient ranged from less than 1 % to 4,5 %, which was not typical levels for the state. Kindergarten was provided for the full day across the 14 schools. | 12 secondary schools | Middle and high schools |
|  | **Dosage** | A summer day-long workshop on RTI and individualized instruction, and ongoing monthly coaching and biweekly classroom-based support | About twice a month, teachers sent in video recordings of class sessions. Trained teacher consultants reviewed these and followed up with 20-30 min. phone conferences. | 5 to 6 cycles of coaching |
|  | **Length** | A school year | 1 school year (13 months) | Two years |
|  | **Description** | A researcher-delivered summer daylong workshop on RTI and individualized instruction (ISI-K). The aim of the intervention is to develop teachers’ abilities to differentiate or individualize instruction - based on on-going assessments of students’ language and literacy skills - to ensure most students learn to read. The ISI-K intervention includes three components: A2i (Assessment to Instruction) software, ongoing teacher professional development, and in-class support. | The My Teaching Partner–Secondary program (MTP-S), a web-mediated approach, "targets the motivational and instructional qualities of teachers' on-going, daily interactions with students." p. 2 Workshop-based training, an annotated video library, and 1 year of personalized coaching followed by a booster workshop. | MTP - web mediated coaching intervention: the primary elements of the MTP-S intervention took place throughout the academic year across the two years of the intervention. Coaches and MTP-S teachers participated in a carefully elaborated and manualized set of ongoing coaching cycles that revolved around review of video recordings of a teacher’s classroom interactions, considered with reference to the CLASS-S dimensions (Pianta et al., 2008a). |
| Control | **Description** | A separate summer daylong workshop on RTI and individualized instruction, but with no ISI-K or A2i training. | Business as usual PD (SOM p. 2) Both I and C teachers participated in 3 hr workshop prior to beginning of school year and also prior allocation. | As usual PD |

| Study |  | Babinski et al., 2018 | Bos et al., 1999 | Brownell et al., 2017 |
| --- | --- | --- | --- | --- |
| Participants | **Country** | USA | USA | USA |
|  | **Professional** | 45 teachers from 12 elementary schools: 15 ESL teachers and 30 classroom teachers | Teachers | 42 teachers |
|  | **Other (e.g. students)** | Young Latino English learners (Elementary schools) | Kindergarten, 1st grade and 2nd grade students | 170 students over 2 rounds (beginning and end of the year) |
| Eligibility | **Professional** | ESL teachers (teaching KG, 1st or 2nd grade). | Not reported | Special education teachers in the 4 districts, 3rd, 4th and 5th grade |
|  | **Other (e.g. students)** | Latino EL's (Spanish as first language) | Not reported | p. 149 "Teachers in both conditions selected one of their reading groups to participate in the study. Groups included students with LD in the third, fourth, or fifth grade." |
| Intervention | **Setting** | Elementary school | Not reported, other than participating schools are located in Amphitheatre, Palominas and Tucson school districts in Arizona. | Schools located in four districts in three states participated in the study during the 2009–2010 school year. |
|  | **Dosage** | 30 minutes per week collaboration | Summer course, 2.5 weeks (3½-hour sessions) and in class support/collaboration (1-hour meetings) once a month during the school year. | A PD institute, six monthly cohort meetings one half-day session and five 90-min meetings and four individual meetings with a coach |
|  | **Length** | 1 school year | A school year | 1 school year |
|  | **Description** | Focus on Developing Collaboration and Consultation Skills. Summer institute - 5 days plus ongoing instructional support (both within school - ESL teacher and 2 classroom teachers that are school based, and with the research team every 6 weeks during the school year) | To support early elementary and special education teachers in integrating explicit instruction (into their curricula) for children at risk of reading and spelling failure. Content focus is on explicit techniques and instructional strategies (phonological awareness, word recognition, spelling skills, fluency). A variety of presentation styles and activities were used in the intervention. | p. 149 "The LLC was designed to deepen special education teachers’ knowledge of how to teach reading using evidence-based strategies, specifically strategies for teaching word study and fluency at the word and passage levels. Each feature of Desimone’s (2009) framework was addressed in the LLC innovation." The intervention included a PD institute (content focussed), active learning cohort meetings (six monthly) and individualised support (observed at 4 monthly cohort meetings). |
| Control | **Description** | Waitlist control | Business as usual | Business as usual |

| Study |  | Buysse et al., 2010 | Cabell et al., 2011 | Dickinson & Caswell, 2007 |
| --- | --- | --- | --- | --- |
| Participants | **Country** | USA | USA | USA |
|  | **Professional** | Teachers | Teachers in the preschool centres | Teachers from Head Start programs |
|  | **Other (e.g. students)** | Latino pre-kindergarten dual language learners (DLLs), four students from each classroom | Five to eight target children from each of 49 teachers’ classrooms | Not relevant |
| Eligibility | **Professional** | Inclusion criteria for teachers: a bachelor's degree or an associate's degree and working towards bachelor's. | Nothing reported | The teacher needed to have at least two college-level early childhood courses. In addition, the teacher needed to attend with his or her supervisor. |
|  | **Other (e.g. students)** | Latino DLLs enrolled in the teachers classes, who had gained parental permission to be included in the study | An age eligibility criterion of 3 years, 4 months, by October of the study year is mentioned | Nothing reported/The children enrolled met the income guidelines set by Head Start |
| Intervention | **Setting** | Participants were selected from the North Carolina More at Four Pre-Kindergarten Program (MAF), a state-funded pre-kindergarten program targeting at-risk four-year-olds (MAF encompasses various types of early childhood programs, such as: public-school based, Head start, Child care centres. Classrooms from all of these types were included in the study). | 38 centres in total: Twenty-seven centres were Head Start programs, and 11 centres were part of a state-funded prekindergarten program delivered in public elementary schools in one mid-Atlantic state. Centres were dispersed throughout the state (16 urban, 12 suburban and 10 rural). | Teachers recruited from Head Start programs across New England over a 2-year period (1998–1999 and 1999–2000) |
|  | **Dosage** | A three-day institute at the beginning of the school year conducted over a period of several weeks. Following the institutes, teachers were assigned to work with one of two bilingual Latina consultants (who were also responsible for facilitating the three-day institutes and community of practice meetings). Each consultant worked with a group of 6–8 teachers for eight weeks, visiting individual teachers once every other week and conducting community of practice meetings with her assigned group of teachers on alternate weeks. | Eight distinct sessions and coaching throughout the academic year. In August preceding the start of the school year, five of the sessions were presented to intervention teachers in a 3-day in-service workshop (i.e., 13 hr of professional development). In January, the remaining three sessions were provided during a 1-day in-service workshop (i.e., 4 hr of professional development). Note: training for the second cohort of teachers was slightly modified with regard to number of fall in-service days [i.e., 2 days], however, total training time of 17 hr were identical to that received by the first cohort. | LEEP was given as a 45-hour course for which participants received four credits from a university in their state. It was delivered in two 3-day intensive sessions (in late October or early November and late February) and support throughout the year |
|  | **Length** | A school year | A school year | The study examines the intervention over a 2 year period |
|  | **Description** | The Nuestros Niños program consisted of three professional development components: (1) professional development institutes to promote teachers’ acquisition of core content knowledge and skills, (2) individualized consultation sessions to support teachers in implementing new instructional strategies in the classroom, and (3) community of practice meetings to provide participating teachers with opportunities for feedback, reflection, and collaborative problem solving. The instructional practices were designed to complement the core curriculum and to provide monolingual English speaking teachers who use English as the primary language of instruction with specific accommodations for DLLs. | The professional development package for teachers in the intervention condition contained two components: (a) direct training designed to increase teachers’ conversational responsibility in the classroom and (b) access to a consultant who provided off-site coaching throughout the academic year. All teachers were requested to submit 12 videos throughout the school year. Research personnel viewed these and provided the teachers with written feedback on a regular basis. | Each session included lectures, videotapes of classroom activity and work samples that participants analysed, and opportunities for participants to break into smaller groups to discuss concepts and relate them to classroom practices. The sessions also provided time for the local teams to plan how they would implement recommended strategies upon their return to their sites. Teachers completed four performance-based assignments. These assignments were designed to help teachers integrate their new understanding of child development with information about effective teaching + improve their capacity to reflect on their practice. Supervisors were enlisted to help support teachers as they adopted new strategies, to provide on-site support for teachers’ academic work, and to help sustain changes after the end of LEEP. |
| Control | **Description** | Business as usual. | Teachers assigned to the control condition also attended a 3-day August in-service workshop and a 1-day January workshop. However, professional development topics did not include conversationally responsive strategies. Control teachers were also provided with and trained to use the video-recording equipment. An identical recording schedule was provided, and teachers submitted videos of specific classroom activities similar to those of the intervention teachers. Control teachers were also given access to consultants who provided either generic feedback regarding best practices in early childhood education or no feedback apart from an acknowledgement that the tape be received. When provided with feedback, teachers had the opportunity to contact their consultant via e-mail. | Business as usual (wait-list control) |
| Notes |  |  | Same trial as reported in Piasta et al., 2012 |  |

| Study |  | Early et al. (2017) | Gallagher et al., 2011 | Garet et al., 2008 |
| --- | --- | --- | --- | --- |
| Participants | **Country** | US | USA | USA |
|  | **Professional** | Pre-K teachers | Sixteen Mentor and 16 Protégé teachers in Head Start classrooms | Teachers |
|  | **Other (e.g. students)** | Preschool children, age 4 | Students from the classrooms | Second grade students |
| Eligibility | **Professional** | Preschool teachers in selected schools and classes, who were (i) NOT in their 1st year as a Georgia Pre-K teacher, (ii) would NOT be absent most of the year due to illness, pregnancy (p. 61) | Experienced teachers who maintained high-quality classroom learning environments. Recommendations of Mentor teachers based on observations from the previous year | None reported |
|  | **Other (e.g. students)** | Preschool children in the state of Georgia's Pre-K program | None reported | Eligibility of districts: Administered a standardized reading achievement test in the second grade. Were not already providing district wide professional development in reading instruction of the same type and level of intensity as that being provided by the Early Reading PD Interventions Study. Were using one of the two scientifically based reading series targeted by the study as the core second grade reading program, and had been using the program for at least one year prior to the study |
| Intervention | **Setting** | Universal Pre-K program, open to all 4 year olds, in variety of settings (private childcare, local schools, Head Start centres, etc.) | Head Start centres in a southern urban setting | Second grade classes in high-poverty schools from six urban school districts across four eastern and Midwestern states that serve substantial numbers of non-English language learner (ELL) students from low-income households. |
|  | **Dosage** | Each teacher participated for 1 academic year. MMCI: 10 two-and-a-half hour workshops delivered over five full day sessions, spread across five months (Oct. /Nov.-Feb./Mar.). One training day/mth. Between sessions, homework assignments. MTP: Sept.-Apr. Cycles of video, review, feedback takes 2 weeks. No pre-specified goal for number of sessions. Average was 7.57. | 50 hour Mentor Teacher seminar in a series of 2-days, sessions over approximately 4 months + Mentors receive ongoing support from Mentor Coordinator. Also, ongoing mentoring of Protégés by the Mentors. | Treatment A: eight institute and seminar days (48 hours of PD). Beginning in late summer and continued through early winter in the school year of the study. Treatment B: In addition to the institute and seminar days, on average, 60 hours of coaching during the school year (approximately 2 hours per teacher per week over a 30 week period) |
|  | **Length** | 3 years (2011-12, 2012-13, 0213-14). New cohorts of teachers selected each year | A school year | A school year |
|  | **Description** | Two intervention conditions: 1) Making the Most of Classroom Interactions (MMCI): face-to-face PD. where small groups of teachers meet for instruction and support. Focus is to learn to identify and analyse effective interactions; 2) My Teaching Partner (MTP): one-to-one remote coaching model, where teachers work with coach using cycles of videotaped observations of teaching, review, and feedback. Teachers receive specific feedback about emotional climate, organisational structure, instructional support based on videos through e.g. conference calls. | To provide ongoing support through mentoring and thus leading to higher quality teachers. The Individualized Learning Intervention (ILI) includes three components: a Mentor Teacher Seminar, Mentors work with their protégés, and support for Mentors via a Mentor Coordinator. In this intervention, Mentors (trained and experienced teachers) coached and advised Protégés (relatively less-experienced teachers). The implementation of the mentoring program was tracked and monitored on a continuous basis by the Mentor Coordinator. | Intervention based on a subset of *Language Essentials for Teachers of Reading and Spelling models*. Focus on topics relevant to second grade reading instruction, relying primarily on the module contents and accompanying trainer materials. Treatment B received on school-site coaching in addition |
| Control | **Description** | During first year of study, control group teachers had access to same online library of video clips demonstrating best practices in teacher-child interactions as MMCI and MTP teachers (no data about usage). In 2nd and 3rd years, control group teachers participated in same 15 h PD required of all Georgia's Pre-K teachers. | Business as usual. | Business as usual |

| Study |  | Gersten et al., 2010 | Hindman & Wasik, 2012 | Howlin et al., 2007 |
| --- | --- | --- | --- | --- |
| Participants | **Country** | USA | USA | UK |
|  | **Professional** | Teachers (1st Grade). 81 first grade teachers in 19 Reading First schools | Teachers | Teachers |
|  | **Other (e.g. students)** | 468 1st Grade Students | Preschool children | Children attending autism-specific classes/units or schools. |
| Eligibility | **Professional** | Working in schools that were involved in the Reading First program. | Teachers at the Head Start centres. | As recruitment of ‘PECS naıve’ classes was not feasible, the requirement for inclusion in the study was that teachers should not have previously received any direct, in-class training/consultancy from PECS consultants. Previous attendance at a PECS workshop was not, of itself, considered grounds for exclusion. |
|  | **Other (e.g. students)** | In classes of participating teachers (7 randomly selected from each class) | Children at the Head Start centres | For inclusion in the study each child was required to: • have a formal clinical diagnosis of autism and to meet criteria for autism or autism spectrum disorder on the Autism Diagnosis Observation Schedule – Generic Module 1 (ADOS-G: Lord et al., 2000); • have little or no functional language (i.e., not exceeding single words/word approximations); • have no evidence of sensory impairment; • be aged between 4 and 11 years; • not be using PECS beyond Phase 1 (i.e., able to exchange symbols only if prompted (Frost & Bondy, 2002)). Classroom eligibility: Each class was required to have a minimum of 3 children meeting the criteria |
| Intervention | **Setting** | 3 large urban school districts (1st grade classrooms) | Head Start centres in a major urban area in the Northeast, all located in high-poverty communities in the inner city. | Schools identified as providing specialist education for children with ASD in Greater London and South East England. |
|  | **Dosage** | 16 interactive sessions, 2 times per month, 75 minutes in duration. Teachers required attending a minimum of 14 sessions to continue in the study and receive compensation. | A 2-day Summer Institute each year and training by expert coaches over 2 years. PD content delivered through month-long cycles of workshops and weekly coaching (coaches spending 3 hours per week with each teacher) | A 2-day PECS workshop for teachers (and parents), which comprised 13 hours of training. Plus 6 half-day, school-based training sessions with expert consultants over 5 months |
|  | **Length** | 1 school year | Two years | Mean time is 17.9 months |
|  | **Description** | Teacher Study Group (TSG). TSG facilitators (5 in total). Summer institute in reading (not sure, if this is control condition too?). 16 interactive sessions (held on school site). Minimum of 14 sessions required for attendance. First 8 - vocab instruction, then 8 comprehension sessions. TSG sessions = 3 to 8 participants, small group meetings in an informal style, 4 step recursive process applied to each meeting (1 - debrief previous application of research; 2 - walk through research, 3 - walk through lesson, 4 - collaborative planning). "Each TSG participant received a copy of Bringing Words to Life: Robust Vocabulary Instruction (Beck et al., 2002), an instructional rubric for evaluating comprehension lessons, and a notebook with selected research-based applied readings in vocabulary and comprehension." (p.20). Designed to enhance teachers' implementation of their reading curriculum using research teaching comprehension and vocabulary. | Teachers were trained on five modules, including (a) oral language, (b) sound awareness, (c) alphabet knowledge, (d) emergent writing, and (e) book reading, a technique that can advance all of these other skill sets. The coach served as an expert who explained and demonstrated best practices and then supported teachers as they emulated these practices and integrated them into their classrooms. | Training in picture exchange communication workshop with expert consultants, feedback, and monitoring. Following each school-based training session class teachers were provided with written summaries, agreed action points and future goals. |
| Control | **Description** | Teaching as usual, including scheduled PD (district and school based). | Business as usual. Control teachers received 2-hour monthly workshops led by their education coordinator (addressing language, literacy, mathematics, science and social development). Control teachers could also request individualized guidance from their coordinator (via phone calls or classroom visits) | Business as usual |

| Study |  | Jayanthi et al., 2018 | Johnson et al., 2017 | Kammermeyer et al., 2016 |
| --- | --- | --- | --- | --- |
| Participants | **Country** | USA | USA | Germany |
|  | **Professional** | 182 first grade teachers. Two hundred and twenty-six teachers (TSG D 115, Control D 111) | Pre-school teachers in 4 schools randomly assigned to I or C (12 teachers in each condition) | Teachers |
|  | **Other (e.g. students)** | 1811 students |  | Kindergarten students |
| Eligibility | **Professional** | First grade teachers | Lead teachers | Nothing mentioned |
|  | **Other (e.g. students)** | Students in the classes of the included teachers, 10 students per teacher |  | The children were selected from kindergartens without any domain-specific promotions until now. |
| Intervention | **Setting** | 16 districts in four states (California, Ohio, Illinois, and Texas). Sixty-two schools were randomly assigned to TSG (n D 31) or control (n D 31) conditions. | Pre-schools in Head Start settings | The kindergartens were all located in Rhineland-Palatinate. |
|  | **Dosage** | p. 88 "10 interactive sessions held at the school site twice a month from October to March. Each session lasted approximately 75 minutes." | 2 1.5 hour training workshops followed by peer coaching and school meeting | A two-day professional training course for teachers in DAA and TRAIN treatments. |
|  | **Length** | 6 months (October - March) | 7 weeks, data collection after 2 months | One school year |
|  | **Description** | Teacher Study Group (TSG), interactive sessions. "The sessions’ scope and sequence were based on Learning How to Improve Vocabulary Instruction Through Teacher Study Groups (Dimino & Taylor, 2009)...Teachers met in groups of between two and seven (depending on school size). Meetings were relatively informal to allow for open discussion and collaboration among teachers. A five-phase recursive process was used during virtually all TSG sessions to provide a common format for the TSG sessions across facilitators and sites, while leaving room for flexibility to respond to issues or concerns specific to members of the group. The format for each session included the following components: (a) Debrief, (b) Discuss the Focus Research Concept, (c) Compare Research with Practice, (d) Plan Collaboratively, and (e) Assignment." (p. 88). | Colleague Observation And Coaching (COACH) program, a peer-coaching program designed to increase teachers’ effectiveness in enhancing classroom quality in a preschool Head Start setting. The COACH program consists of a training workshop on coaching skills and student-teacher interactions, six peer coaching sessions, and three centre meetings. The COACH program aimed to improve student–teacher interactions through peer observation, peer discussions, and reﬂection. | DAA: The so-called ‘Kindergarten of the Future in Bavaria’ Approach is not a programme with clear instructions. It is oriented towards the curricular dimension of ‘strong classification’ because the curriculum includes domain-specific academic subjects and can be classified as ‘weak framing’, which means that the child has more apparent control. The professional training was conducted by researchers who were involved in developing this approach. TRAIN: The so-called ‘Letter & Number World Approach’ is a training that can be classified as ‘strong classification’ and ‘strong framing’. It focuses on numbers and operations and is characterised by lessons in which teachers introduce one number or one letter after the other, respectively, based on detailed instructions. The professional training course was provided by the head of the ‘Institute for preschool education’. |
| Control | **Description** | p. 89: "Teachers in the control condition did not engage in the TSG or have access to the materials made available to teachers in the TSG condition during the course of the study. However, they did attend other PD activities related to literacy." | Teaching and professional development as usual, which consisted of regularly scheduled in-service programming provided to all schools; the treatment schools continued to receive these services as well | Business as usual |

| Study |  | Landry et al., 2009 | McCollum et al., 2013 | Murphy et al., 2017 |
| --- | --- | --- | --- | --- |
| Participants | **Country** | USA | USA | UK |
|  | **Professional** | Teachers | Teachers | Year 4 and 5 teachers |
|  | **Other (e.g. students)** | At-risk preschool children | Pre-kindergarten children | Year 4 and 5 students |
| Eligibility | **Professional** | Teachers were required to follow a published curriculum, but they were not required to follow any particular published curriculum | Certified teachers from state-funded prekindergarten classrooms located in public schools | "Schools that were selected into the treatment group were asked to select three teachers to be part of the trial. Schools had complete autonomy over which teachers they chose, on the condition that these teachers were to teach Years 4 and 5." p. 10 |
|  | **Other (e.g. students)** | Nothing mentioned | Nothing mentioned | In Year 4 and 5 of the participating schools |
| Intervention | **Setting** | This multisite study took place in Ohio, Maryland, Florida, and Texas. Participating schools primarily served children and families from low-SES backgrounds | Teachers were recruited from two early childhood programs in a medium-sized Midwestern community and one cooperative program that served the surrounding county. | Primary schools (with above average FSM) |
|  | **Dosage** | All teachers in the four professional development conditions attended 2-hr eCIRCLE classes twice per month for nearly the entire school year. Approximately half of the teachers also received 4 hr of in-classroom mentoring each month. | Prior to beginning the school year, teachers in the coaching group met on two consecutive days for orientation (10 hr total). Thereafter biweekly coaching over a full year (total of 15 visits per classroom). Three brief additional group meetings (1.5 hr each) were held during the year. | Full day introductory conference, followed by a further 2 full days and 2 half days |
|  | **Length** | One school year | One school year | 2 years |
|  | **Description** | Four treatment conditions: All 4 PD conditions included the same yearlong, facilitated online course that emphasized language and literacy instruction, practice of learned material in one’s classroom, and participation in online message boards with fellow teachers. Some teachers received both in-classroom mentoring and detailed, instructionally linked feedback concerning children’s progress in language and literacy. Some teachers received no mentoring but did receive the detailed, instructionally linked feedback concerning children’s progress. Some teachers received in-classroom mentoring but only limited feedback on children’s progress, which was not linked to curricular activities. Finally, some teachers received no mentoring and only limited feedback concerning children’s progress. | Help teachers use research-based instruction to teach emergent literacy skills to young children. Coaching visits: Each session included (a) a brief preobservation discussion to re-establish the focus and context of the observation and to review percentage data from the previous session, (b) teaching-observing, (c) meeting to view and discuss the data and compare it with previous observations and with the 80% criterion, and (d) looking forward to what the teacher might do differently on the next visit. Joint inspection and discussion of data obtained during the observation served as the primary strategy for coaching. | "Lesson Study is a process incorporating teacher observations and feedback. It is a professional development programme with a long history of use in Japan and is being increasingly used in the U.K. and worldwide. Teachers work in small groups to plan lessons that address a shared teaching and learning goal. For this intervention, these groups consisted of a learning tripod with three teachers from the same school, one of whom would be the selected on the basis of being the ‘expert teacher’." (p. 6) |
| Control | **Description** | Business as usual | Business as usual | Business as usual |

| Study |  | Neuman & Cunningham, 2009 | Olson et al., 2016 | Parkinson et al., 2015 |  |
| --- | --- | --- | --- | --- | --- |
| Participants | **Country** | USA | USA | USA |  |
|  | **Professional** | Teachers and providers in child care centres | 95 secondary school teachers were randomly assigned, separately for each grade within each school, to the Pathway or control condition. The study sample consisted of 49 Pathway teachers and 46 control teachers | Teachers at 78 schools |  |
|  | **Other (e.g. students)** | No child outcomes provided | Students randomly assigned to teachers' classrooms | K, first and second grade children |  |
| Eligibility | **Professional** | To be eligible for the project, practitioners needed to meet four criteria: (a) They needed to be open to taking a course at their local community college in pursuit of an associate’s degree in early childhood education; (b) they had to be employed at least 20 hours per week in a licensed child-care centre or home; (c) they had to care for children ages 3 to 5; and (d) they needed to have an agreement from their sponsoring organization (centre director or educational director) if they taught in a centre. Only one teacher per centre or family/group home provider was eligible to participate in the project | Secondary school teachers |  |  |
|  | **Other (e.g. students)** | Not relevant. | Secondary school students |  |  |
| Intervention | **Setting** | Child-care centres in high-priority urban areas, serving the very poorest children in Michigan’s poorest cities. Detroit and midsized cities in urban counties, including Flint, Grand Rapids, and Lansing, constituted priority urban centres targeted. | Secondary schools |  |  |
|  | **Dosage** | A 45-hour, three-credit course (3-hour classes over a 15 week period) and a yearlong coaching intervention (weekly sessions, 1-1½ hours in length, a total of 32 sessions) | 46 hr of training each school year (via six 6-hr released days interspersed throughout the school year and five 2-hr after-school sessions) focused on methods for helping Latinos and mainstreamed ELs to develop academic literacy | K and first-grade teachers received services beginning in Year 1. Teachers in these grades who remained in the CLI program for all three study years could receive up to 186 hours of CLI professional development and coaching. Second-grade teachers began receiving services in Year 2. Second-grade teachers who remained in the CLI program through Year 3 of the study received up to 147 hours of CLI professional development and coaching |  |
|  | **Length** | One school year | Two years | Three years |  |
|  | **Description** | The researchers developed a three-credit course in early language and literacy designed to provide students with content knowledge considered by experts to be essential for quality early language and literacy practice. Each class used a lecture format to present the week’s topic, followed by simulation and hands-on activities designed to link theory to practice. The courses were taught by experienced early childhood faculty. Coaching focused on helping participants apply research-based strategies to improve child outcomes in language and literacy. Coaching was performed on-site. The coaching sessions for the first 15 weeks were aligned with the professional development course. Coaches engaged teachers in reflection and goal setting; the coaches helped to identify desired outcomes and strategies to achieve these outcomes; collaboratively, they developed an action plan for the implementation of new practices the following week, which became the source of further reflection and action. | Pathway Project: a cognitive strategies approach to text-based analytical writing. 46-hr professional development program in which secondary teachers learn how to integrate cognitive strategy instruction into process writing to improve students’, specifically Latinos’ and mainstreamed ELs’, interpretive reading and text-based analytical writing by (a) using a cognitive strategies approach to reading and writing instruction, (b) instructing students to revise a pre-test on-demand writing assessment into multiple draft essays, and (c) receiving ongoing support from experienced Pathway Project teachers who serve as coaches to teachers. | CLI Children's Literacy Initiative Program: Key features: Providing teachers with literacy resources, including book collections to create a literacy-rich classroom environment; conducting professional development institutes and seminars to train teachers in strategies and techniques for literacy instruction, followed by classroom-embedded coaching to help teachers apply these strategies in the classroom; identifying one model classroom teacher per grade who receives intensive coaching and support and in whose classroom additional embedded coaching could be provided; educating school leaders on how to leverage CLI training to sustain high-quality literacy instruction in the school. |  |
| Control | **Description** | Business as usual | Teaching as usual using the district English language arts textbook and core novels for teaching. Both groups attended one full day of professional development led by district curriculum specialists on protocols for reviewing district benchmark assessments | Usual professional development |  |

| Study |  | Pianta et al., 2017 | Powell et al., 2010 | Rimm-Kaufmann et al., 2014 |  |
| --- | --- | --- | --- | --- | --- |
| Participants | **Country** | USA | USA | USA |  |
|  | **Professional** | Pre-K teachers | Teachers (in Head Start centres) | 3rd, 4th and 5th grade teachers |  |
|  | **Other (e.g. students)** | 4 year old children | Pre-kindergarten children | 2nd grade to 5th grade students |  |
| Eligibility | **Professional** | Lead teacher in pre-K classroom teaching in English with access to the internet | All lead teachers in each of the five Head Start programs were eligible for study participation |  |  |
|  | **Other (e.g. students)** | No IEP, spoke English or Spanish; 4 years old | All children who were 4 years of age by December 31 of the year in which their teacher participated in the study were eligible to participate in the study |  |  |
| Intervention | **Setting** | Head Start and other pre-K classrooms | 24 centres across five Head Start programs that collectively serve 11 counties in a Midwest state. One program served an urban area, two were located in small cities and two served rural counties. | Elementary schools |  |
|  | **Dosage** | One year or two years of intervention | A 2-day workshop (16 hr total) followed by expert coaching (biweekly, 7 session across 15 weeks) | 2 one-week long training sessions; 3 day training sessions and 3 consultations with administrators each year |  |
|  | **Length** | Two years: 14 weeks of effective teacher - pupil interactions intervention followed by MTP; follow-up year | One semester | Two years |  |
|  | **Description** | MTP: individualized web-mediated feedback to teachers based on analysis of videos of their own interactions with children. Cycles of coaching include video clips of teachers’ interaction and coaches’ feedback and questions, which are intended to build knowledge, observation skills, and the capacity to observe and analyse moment-to-moment behaviour with children | The goal of the one-semester PD intervention, entitled Classroom Links to Early Literacy, was to improve teachers’ use of evidence-based literacy instruction. The 2-day workshop provided an overview of the intervention content, with emphasis on demonstration and guided discussion of evidence-based practices. A stated goal of the workshop was to promote the development of supportive relationships between coach and teacher. Coaches were university employees. The coaching protocol followed an observe–assess–recommend sequence wherein the coach observed a specific instructional practice determined in advance with the teacher. Based on the observation, the coach provided two types of written feedback: statements about appropriately implemented aspects of the targeted practice and recommendations for improving the practice. | Responsive Classroom (RC) approach is a PD programme that focuses on a set of practical teaching strategies designed to support children's social, academic, and self-regulatory skills. The RC approach emphasizes how to teach rather than what to teach. Instead of establishing a set curriculum for teaching SEL skills the RC approach embeds modelling of prosocial behaviour, collaboration, and self-control into instructional practice |  |
| Control | **Description** | Usual PD | Participation in intervention in next semester. | Usual PD with waitlist |  |
|  |  | Same trial as Ansari & Pianta, 2018, Hamre et al., 2012 and Sandilos et al., 2018 |  | Same trial as Ottmar et al., 2013 |  |

| Study |  | Saraniero et al., 2014 | Scanlon et al., 2008 | Schwanenflugel et al., 2010 |
| --- | --- | --- | --- | --- |
| Participants | **Country** | USA | USA | USA |
|  | **Professional** | Teachers | Teachers | Teachers |
|  | **Other (e.g. students)** | Students, 3rd and 4th grade | Kindergarten students | Pre-kindergarten children |
| Eligibility | **Professional** | Third and fourth grade teachers, had to teach in a school where 35% of the students qualified for free or reduced lunch and where the school was located in one of ten participating districts | Nothing mentioned | Nothing mentioned |
|  | **Other (e.g. students)** | Students of the teachers | Nothing concerning students is mentioned. Schools were eligible to participate in the study if they 1) served a relatively high number of low-income students, 2) offered full day kindergarten; and 3) were within 50 miles of our research centre in Albany, New York. | Only children who were native English speakers according to parental report took part in this study. |
| Intervention | **Setting** | Ten school districts, ranging from rural communities to mid-sized cities. | Fifteen schools from ten districts in New York State (six urban and four rural) all serving a relatively high number of low income students | The teachers and students were drawn from 37 classrooms funded by the state universal (open to all 4 year olds regardless of family income) prekindergarten program located in rural areas in a southeastern state. School district policies required all teachers to meet state certification levels |
|  | **Dosage** | A weeklong summer institute, receiving approximately 30 hours of professional development and half of the intervention group also received on average 25 hours of instructional arts coaching during the school year. The half that did not receive coaching throughout the school year had to implement their arts integration lessons and learning independently. | A 3 day workshop | The experimental teachers received professional development through (a) a three-day institute held immediately prior to the beginning of school, (b) a two-hour on-site follow-up workshop conducted approximately two weeks after the start of the intervention, and (c) on-site, as classroom-based support throughout the intervention phase by preliteracy specialists who visited the classrooms every two to three weeks. |
|  | **Length** | One school year | Data collection after one school year | A 15-week intervention period; observations were completed within six weeks following professional development and again within six weeks of the end of the school year. |
|  | **Description** | Professional development in arts integration, specifically focusing on integrating theatre and visual arts into reading. Teachers received standard based instruction from instructors, who modelled lessons for 3rd and 4th grade arts and reading curricular. Half of the intervention group received coaching during the school year too. | All approaches utilized the Interactive Strategies Approach (ISA) and implemented a small group intervention. PD teachers participated in a 3-day workshop concerned with the Interactive Strategies Approach during the summer prior to teaching. They were provided with a handbook and access to the ISA PD website, which included additional teaching ideas. Focused on developing teachers' knowledge in order to enable them to more fully understand their students' needs. It also provided tools, in the form of techniques and activities, which teachers could select, as appropriate, to help their at risk students make the accelerated progress needed in order to meet grade level expectations. Major emphasis was placed on the need to include small group, differentiated instruction. | Comprehensive preliteracy program guidelines to support the development of foundational preliteracy skills in 4 year olds. Emphasis on theory and research related to the recommended classroom practices and provision of active learning experiences for developing classroom materials. |
| Control | **Description** | Business as usual | Small group instruction using the Interactive Strategy Approach | Business as usual |

| Study |  | Snow et al., 2014 | Wasik & Hindman, 2011 | Yoshikawa et al., 2015 |
| --- | --- | --- | --- | --- |
| Participants | **Country** | Australia | USA | Chile |
|  | **Professional** | Primary school teachers in disadvantaged settings and school principals | Teachers (in Head Start centres) | Teachers and teachers' aides at 64 pre-schools |
|  | **Other (e.g. students)** | n = 89, Grade 1 and Grade 2 students | Pre-kindergarten children | Pre-K and K children |
| Eligibility | **Professional** | Teachers in low SES primary schools | Nothing mentioned |  |
|  | **Other (e.g. students)** | Parental consent | Nothing mentioned |  |
| Intervention | **Setting** | Low SES Primary schools | Three Head Start centres, nothing else reported | Pre-K and K classrooms |
|  | **Dosage** | 6 days teacher and principal PD. Then continuing contact in school. | A summer literacy institute over 4 half-days and thereafter, coaches provided 9 months of 3- to 4-week training cycles. Each cycle began with coaches providing a 3-hr group training for the teachers | 6 month-long modules per year |
|  | **Length** | 18 months | One school year | Two years |
|  | **Description** | "The OLSEL intervention. Teachers and principals were exposed to a range of activities that can be incorporated into the early-years classroom, using Munro ’ s (2007, 2011) “ ICPALER ” — I deas –C onventions – P urposes – A bility to L earn – E xpression and Reception Framework." (p. 499). "The OLSEL PD focused on the link between early oral language competence and the emergence of literacy and academic success, and involved both teachers and school principals, in keeping with the notion that curriculum change needs organizational support as well as changed knowledge and skills (Deal & Petersen, 1990)." (p. 500). Leaders of OLSEL in each school enrolled in a relevant Masters module at Melbourne University. Four language domains targeted - phonemic and phonological awareness, vocab. knowledge, awareness and application of story grammar, and comprehension and use of longer more complex sentences. | The PD intervention provided teachers with conceptual knowledge and instructional strategies that support young children’s development of vocabulary, alphabet knowledge, and phonological sensitivity. In addition to training, the teachers received books of various genres and theme guide with daily scheduling suggestions. Training cycle consist of following elements: groups training, in-class modelling, in-class observation. | UBC: Un Buen Comienzo is a 2-year program that provides professional development to pre-kindergarten and kindergarten teachers in Chile, with the goal of enhancing children’s language, literacy, health, and socioemotional outcomes through workshops and in-classroom coaching. The UBC program consisted of 12 modules combining didactic (content-focused strategies) and coaching components, and provision of a library of roughly 100 books per classroom. |
| Control | **Description** | Usual teaching | Business as usual | Much reduced intervention: comparison condition: 10 books (rather than 100) were distributed per classroom, and one workshop on self-care and stress reduction was provided for teachers and aides |

| Ottmar et al., 2013 | Piasta et al., 2012 | Ansari and Pianta, 2018 | Sandilos et al., 2018 | Hamre et al., 2012 |
| --- | --- | --- | --- | --- |
| Same trial as Rimm-Kaufman et al., 2014. Ottmar et al., 2013 reports only teacher outcomes for one of the years whereas Rimm-Kaufman et al., 2014 reports student outcomes for all years. | Same trial as reported in Cabell et al., 2011. Cabell et al., 2011 reports on student outcomes and Piasta et al., 2012 reports on students (but transcribed from video only, another outcome than reported in Cabell et al., 2011) | Same trial and data as Pianta 2017 (student outcomes). Same trial as Sandilos et al., 2018 and Hamre et al., 2012 (teacher outcomes) | Same trial as Pianta et al., 2017 and Ansari & Pianta, 2018 (student outcomes) and same trial and data as Hamre et al., 2012 (teacher outcomes) | Same trial as Pianta et al., 2017 and Ansari & Pianta, 2018 (student outcomes) and same trial and data as Sandilos et al., 2018 (teacher outcomes) |

### Stress reduction

| Study |  | Flook et al. (2013) |
| --- | --- | --- |
| Participants | **Country** | US |
|  | **Professional** | Elementary school teachers |
|  | **Other (e.g. students)** | N/A - focus solely on professionals |
| Eligibility | **Professional** | Nothing reported |
|  | **Other (e.g. students)** | N/A - focus solely on professionals |
| Intervention | **Setting** | 18 public elementary school teachers |
|  | **Dosage** | The course lasted eight weeks, 2.5 hours/week, plus a day-long immersion (6 hours), totalling approximately 26 hours of group practice and instruction |
|  | **Length** | Eight weeks |
|  | **Description** | Mindfulness-Based Stress Reduction course. The course (MBSR) was offered during the academic year in Fall 2011, which allowed teachers to directly apply the skills they learned within the context of their classroom and actual teaching. Outside of class, teachers were encouraged to practice between 15-45 minutes per day for six days/week and were provided with guided recordings to support their practice. |
| Control | **Description** | Business as usual |

## Appendix E: Risk of Bias Assessment

The RoB tool is provided in the Methods section. In the 5 point scale, 1 corresponds to Low risk of bias and 5 corresponds to High risk of bias.

### Social and emotional development

| Study | Fukkink & Tavecchio, 2010 | Hickey et al. (2017) | Jennings et al. (2017) |
| --- | --- | --- | --- |
| Sequence generation (Judgement) | Unclear | Low | Low |
| Sequence generation (Description, quote from paper or describe key information) | Not described, other than centres were randomised and all classrooms in same centre assigned to same condition. | An independent statistician used a computer-generated random number sequence to randomly and blindly allocate teachers on a 1:1 basis to an intervention or waiting list control group. Randomisation was conducted within schools, two teachers from each school | Two cohorts (different years and schools) randomised within school (possibly, not within grade, there is a 10% difference in grade level.): "We utilized a block randomization method to randomize participants into groups of approximate equal sample size within schools. This was achieved by establishing a set block size for each school, and then generating all possible balanced combinations of assignments within the block using a computer generated random-number sequence, with a new random-number seed introduced for each iteration. Randomized blocks were then randomly chosen to determine participants’ assignment to groups" (p. 6). |
| Allocation concealment (Judgement) | Unclear | Low | Low |
| Allocation concealment (Description, quote from paper or describe key information) | Not mentioned |  |  |
| Blinding (Judgement) | 3 | 4 | 3 |
| Blinding (Description, quote from paper or describe key information) | “The rating (or scoring) of the video clips was carried out following a “blind procedure,” with assessors who had not been informed of the time of measurement (pre-test, post-test or retention measurement) or condition (VIG or control)” (p. 1656). Different assessors used to rate individual teachers. | Baseline assessments were completed before randomisation. Student outcomes are teacher assessed outcomes | Coders were blind to condition (p. 7) |
| Incomplete outcome data addressed (Judgement) | Unclear | 1 | 2 |
| Incomplete outcome data addressed (Description, quote from paper or describe key information) | Only explicit mention of attrition from post-test to retention measurement, i.e. For the intervention group (9 out of 52). Not clear whether this means that attrition from pre-to post-test is zero or not. No numbers provided in tables. They do not report how many centres are randomised | Student attrition: 5% treated and 7% control. No teacher attrition | Stated p. 5 that attrition was low at 6% (five from control, eight from intervention) at post-test (7% treated and 6% control). However, later (p. 9) it is stated that attrition was 7% (15 teachers) |
| Free of selective reporting (Judgement) | 3 | 1 | 1 |
| Free of selective reporting (Description, quote from paper or describe key information) | Not clear why control group was not measured 3 months after intervention stopped, only intervention group. |  |  |
| Free of other bias (Judgement) | 4 | 2 | 2 |
| Free of other bias (Description, quote from paper or describe key information) | P. 1653: The VIG method, which is widely used in the Netherlands, is an integral part of a masters course School-Video Interaction Guidance (S-VIG) in the Netherlands. Unclear if the intervention is VIG training or is it VIG in itself. Unclear if all involved teachers use VIG and some then get training. If the intervention is not training, it is possible that centres using VIG and centres not using VIG have been chosen (perhaps randomly)in which case it is not a RCT | Students selected for participation based on SDQ score (low medium and high scores) before randomisation. Teacher and students reasonably balanced on demographics (table 1). Students balanced on pre-test (SDQ) (table 2 and 4), some large imbalances on teacher behaviour | Teachers balanced on ethnicity and class size (table 1) but there is a 10%-point difference in grade level. Teacher gender, age, years teaching and education not reported by group, only total. Teacher reported proportion of students with an IEP or 504 plan, proportion of students ever suspended, and teacher report of students’ average learning support at home also shown (no imbalances). Pre-test shown in table 2, reasonably balanced (only CLASS outcomes considered). The study design (randomization within schools) increase the risk of spill over effects to the control group (and presumably downward bias). |
| A priori protocol (Judgement) | Unclear | Unclear | Unclear |
| A priori protocol (Description, quote from paper or describe key information) | Not mentioned. |  |  |
| A priori analysis plan (Judgement) | No | Unclear | Unclear |
| A priori analysis plan (Description, quote from paper or describe key information) | Not mentioned |  |  |
| Confounders | N/A | N/A | N/A |

| Study | Jensen et al., 2015 | Murray et al. (2018) | Murray et al., 2014 (2018) |
| --- | --- | --- | --- |
|  |  |  |  |
| Sequence generation (Judgement) | Unclear |  | Unclear |
| Sequence generation (Description, quote from paper or describe key information) | Unclear (p. 28), pre-schools are randomised (cluster randomisation) |  | Within school randomisation of grades (K-2).Method not described |
| Allocation concealment (Judgement) | Unclear |  | Unclear |
| Allocation concealment (Description, quote from paper or describe key information) | Not mentioned |  | Not mentioned |
| Blinding (Judgement) | 4 |  | 3 |
| Blinding (Description, quote from paper or describe key information) | No blinding of outcomes (teacher administered SDQ) |  | Trained research assistants blind to randomization status observed teachers |
| Incomplete outcome data addressed (Judgement) | 3 |  | 1 |
| Incomplete outcome data addressed (Description, quote from paper or describe key information) | p.30 "the overall attrition rate was 22%, but with an uneven distribution across the intervention and control groups with significantly different attrition rates of 16% and 28%, respectively." Appendix Table A1 (ref. p. 30) - attrition rates but only p values reported. Children with weaker outcomes more likely to attrit (p. 30). |  | Attrition of n = 2 teachers in intervention group and 0 in the control group (total 2%). Very low attrition |
| Free of selective reporting (Judgement) | 3 |  | 3 |
| Free of selective reporting (Description, quote from paper or describe key information) | Attrition, reasons stated (residential moves etc.) - more attrition in control group. Table A.1 in appendix - means not reported for attrition, only p values. Not ITT analysis. Only give standardised means in tables (no raw scores in Table 2). Sensitivity analysis. Teacher attrition not reported. |  | According to note at bottom of Table 1, there is also a TCI Harsh component. Reason for non-reporting is not provided. |
| Free of other bias (Judgement) | 4 |  | 4 |
| Free of other bias (Description, quote from paper or describe key information) | "Originally, the intervention was also implemented in a fourth municipality. However, the data on child outcomes for the control group in this municipality showed unexpected high SDQ-scores. This amounts to relatively few children in the final sample of children present for the full period, and thus could not be detected in the original balancing tests. The available data did not allow us to investigate the reasons behind this further and the current study therefore only includes data from three municipalities." (p. 28). Late entrants not in sample. Analytic sample consists of children present for the full period of two years. |  | Not reported if parental consent to use student data was obtained before or after randomisation. Treated/control distribution of grades not reported. Not reported if some teaches teach more than one grade. Worst case a teacher may teach a class in a treated grade and a control grade. |
| A priori protocol (Judgement) | Unclear |  | Unclear |
| A priori protocol (Description, quote from paper or describe key information) | Not mentioned |  | Not mentioned. |
| A priori analysis plan (Judgement) | Unclear |  | No |
| A priori analysis plan (Description, quote from paper or describe key information) | Not mentioned |  | Not mentioned |
| Confounders | N/A |  | N/A |
| Notes |  | Same trial and content as Murray 2014 |  |

| Study | Raver et al., 2009 | Reinke et al. (2018) | Reinke et al. 2016 (2018) |
| --- | --- | --- | --- |
| Sequence generation (Judgement) | Low |  | Unclear |
| Sequence generation (Description, quote from paper or describe key information) | Random numbers generator (p.7) to assign sites (n=18) stratified by cohort and in matched pairs to I and C |  | Classrooms randomised in three sequential, annual cohorts within school. Method not reported |
| Allocation concealment (Judgement) | Low |  | Unclear |
| Allocation concealment (Description, quote from paper or describe key information) | Matlab uniform random numbers generator. It is non-sequential and therefore concealed. |  |  |
| Blinding (Judgement) | 3 |  | 4 |
| Blinding (Description, quote from paper or describe key information) | A cadre of 12 trained observers (blind to intervention status of each site as well as to the approaches taken by training and MHCs) collected class-room level data.' (p.8). |  |  |
| Incomplete outcome data addressed (Judgement) | 2 |  | 2 |
| Incomplete outcome data addressed (Description, quote from paper or describe key information) | Attrition: teachers (4/87); children (88/543). 54 children entered late. Attrition analyses limited to children due to low number of teacher attritors: comparison of 455 children at baseline with 93 children who left (no sig. differences); comparison of 455 children at baseline with 59 who entered late (only difference children who entered late were younger). No sig. differences between exit and entry status and I and C on 8 demographic variables. More girls entered late. Page 10 |  | No teacher attrition. 7.4% attrition of treated students and 7.6% for control. The missing rates for the post-tests of eight outcome measures range from 6.4% to 7.3% in the overall sample. The maximum differential missing rates between the treatment and control groups are 2.70% for the pre-test and 0.70 for the post-test. |
| Free of selective reporting (Judgement) | 3 |  | 2 |
| Free of selective reporting (Description, quote from paper or describe key information) | Results for CLASS (4 subscales) reported in Tables 1, 2 and 3. No raw means for either pre or post intervention is shown, only covariate adjusted means and standard errors. |  | Post raw means not reported |
| Free of other bias (Judgement) | 2 |  | 2 |
| Free of other bias (Description, quote from paper or describe key information) | Evidence from descriptive statistics suggests substantial variability in classroom quality among sites. Page 11 Note that of the scores mentioned here only the ECERS-R is a baseline score, the rest are post intervention (mention that the CLASS positive climate is from March, which is post, does not explicitly mention month for the three remaining but they are probably also March scores). With all that variability, pre as well as post, it is even more worrying that they do not show raw means by group (only covariate adjusted means). The overall variability post intervention could be a result of the treatment (if the variation is mainly between groups). |  | Student demographics reasonably balanced except small imbalance on grade (table 1). Small imbalance on pre-tests (table 1) The teacher reported pre-tests are balanced (table 1) but small imbalance on the two standardised academic achievement tests. No teacher demographics reported by group |
| A priori protocol (Judgement) | Unclear |  | Unclear |
| A priori protocol (Description, quote from paper or describe key information) | Not mentioned |  |  |
| A priori analysis plan (Judgement) | Unclear |  | Unclear |
| A priori analysis plan (Description, quote from paper or describe key information) | Not mentioned |  |  |
| Confounders | N/A | N/A | N/A |
| Notes |  | Same trial and same content (except no descriptive table with pre-test) as Reinke, 2016 |  |

| Study | Rubie-Davies et al. (2015) | Seabra-Santos et al. (2018) | Snyder et al. (2018) |
| --- | --- | --- | --- |
| Sequence generation (Judgement) | Low | Unclear | Unclear |
| Sequence generation (Description, quote from paper or describe key information) | P. 76: a research assistant not involved in the study was blindfolded and drew names from a container for each school and the assignment was recorded by a further research assistant. When there were an uneven number of teachers participating in a school, more teachers were randomly assigned to the intervention group than to the control group. | Randomized matched pairs of classrooms (based on economic need and classroom size) although teachers located in the same preschool or school cluster were randomized as blocks. Method not reported | Teachers within 3 districts randomised, method not reported other than it was 'simple' (p. 216). Eligible students randomly selected before randomisation of teachers (three students per teacher) |
| Allocation concealment (Judgement) | Low | Unclear | Unclear |
| Allocation concealment (Description, quote from paper or describe key information) | Non-sequential and therefore low risk of bias |  |  |
| Blinding (Judgement) | 2 | 4 | 3 |
| Blinding (Description, quote from paper or describe key information) | Teachers administered tests, albeit according to a protocol. Researchers entered paper-and -pencil responses online. Positive aspect: Statistical analyses of data was conducted centrally. | "Psychologists who were unaware of the classroom allocation to the experimental/ control condition conducted all the evaluation process" (p. 3914). Not sure, what is meant by this as teachers completed the behaviour rating scales used. | All dependent measures data were collected by project personnel who were naïve to teachers’ and children’s experimental conditions |
| Incomplete outcome data addressed (Judgement) | 2 | 1 | 2 |
| Incomplete outcome data addressed (Description, quote from paper or describe key information) | Attrition: 7% left the study during the year due to personal reasons, promotion, and retirement. | Three teachers and 61 children were lost at post intervention assessment. Treated: 2 teachers (6%) and 38 students (7%); control 1 teacher (3%) and 23 students (5%) | One teacher withdrew (3%) and student attrition was 8% (including the three target children in the teacher’s classroom who withdrew). For the remaining students there is almost no missing data except the Test of Early Reading Ability, only reported for 78 of the 97 students (missing rate 20%) |
| Free of selective reporting (Judgement) | 1 | Unclear | 2 |
| Free of selective reporting (Description, quote from paper or describe key information) |  | Unclear what is reported in table 2. The authors write on p. 3913: "When compared to the national standards for the PKBS-2 (Major 2011), these children had low average scores on the Social Skills scale (M= 79.69, SD = 17.92), corresponding to a percentile of 32 (the descriptive statistics for the national sample are M= 83.84, SD = 13.33). As for the Problem Behavior scale (M= 36.25, SD = 27.37), the average score was close to the national mean (M= 36.78, SD = 24.34), corresponding to a percentile of 52." but the estimated marginal means (probably with the between classroom SD 'portioned out', this is how the authors' term it, usually between classroom SD is added to get the total SD) are 77.49 (31.06), 87.82 (31.30), 81.49 (30.26), 86.99 (30.45) and 38.87 (73.25), 31.65 (73.62), 36.92 (71.21), 35.56 (71.44). Lack most of the model results | Effect sizes, Glass’s Δ and cluster corrected p-values only reported for outcomes that are significant and/or 'noteworthy (i.e. > 0.2 in absolute terms) |
| Free of other bias (Judgement) | 2 | Unclear | 2 |
| Free of other bias (Description, quote from paper or describe key information) | First author created the tests using the e-asTTIe tool. It was then reviewed by deputy principals of involved schools. | Small imbalance on student Entitled to free lunch (table 1). Teachers reasonably balanced (table 1). Student pre-test reasonably balanced although unclear if it is raw pre-test scores reported in the table (table 2). Social skills difference at pre-test is around 0.3 SD. It is unclear how the differences between treatment and control group can be significant at so low levels, all effects are < 0.2 standard deviations, and they do not have that much statistical power. | Teacher, classroom, and pre-test (the three CLASS summary measures) reasonably balanced (table 1 and text p. 217). Some imbalances on student characteristic (table 2). Some (0.22-0.3 SD) imbalance on 3 of 4 student pre-tests (table 3). Pre-tests seem to be conducted after randomization (p. 216). |
| A priori protocol (Judgement) | Unclear | Unclear | Unclear |
| A priori protocol (Description, quote from paper or describe key information) | Not mentioned |  |  |
| A priori analysis plan (Judgement) | Unclear | Unclear | Unclear |
| A priori analysis plan (Description, quote from paper or describe key information) | Not mentioned |  |  |
| Confounders | N/A | N/A | N/A |

### Language and literacy

| Study | Al Otaiba et al., 2011 | Allen et al. (2015) | Allen et al., 2011 |
| --- | --- | --- | --- |
| Sequence generation (Judgement) | Unclear | Unclear | Unclear |
| Sequence generation (Description) | Schools matched in pairs on proportion of students who received free or reduced-price lunch, Title I and Reading First participation and the schools’ reading grades (proportion of students passing the Florida high-stakes reading test at third grade) and one in each pair randomly assigned to intervention/wait list. Method not reported | Teachers were stratified within school, within grade level (high school vs. middle school) and within course content area (language arts/social studies/history vs. math/science) then assigned randomly to the MTP-S coaching condition or to a control group. Method not reported | Not described. |
| Allocation concealment (Judgement) | Unclear | Unclear | Unclear |
| Allocation concealment (Description) |  |  | Allocation concealment is not clearly described. (p. 2-3 in SOM) |
| Blinding (Judgement) | 4 | 4 | 3 |
| Blinding (Description) | No information concerning blinding is provided | Nothing reported | Not entirely clear, but since the testing system used is part of an official state testing system, it seems as though it would be free from potential teacher bias. |
| Incomplete outcome data addressed (Judgement) | 2 | Unclear | 3 |
| Incomplete outcome data addressed (Description) | Apparently no teacher attrition. Of those students with parent consent 8% left during the year, not reported by group other than attrition was evenly distributed across conditions | 97 teachers were selected to participate in the study. Of these, 86 completed both years of the intervention, attrition not reported by group (p. 8). Nothing concerning student attrition or missing data is reported, only that sixty-four percent of invited students agreed to participate (p. 5) | Attrition: According to the text, 78 teachers participated in the intervention year, i.e. were randomised. In table S1, total number is 76. In post-intervention year ( study is designed to measure effects one year after intervention has been completed and with new/different students), 61 teachers had both fully participated in evaluation and had end of year achievement tests for students. (SOM p. 8) I.e. attrition is 15 teachers out of 76. Reasons for attrition at this stage incl. teaching a course with no end of year test, residential moves, declining further participation due to other tasks. Authors claim no selective attrition at this stage, see SOM p. 8 |
| Free of selective reporting (Judgement) | 4 | 2 | 3 |
| Free of selective reporting (Description) | Teacher characteristic not shown by group. Results of the Picture Vocabulary (PV) subtest of the WJ-III not shown. Post means only shown for the two DIBELS tests even if students are tested within the first 20–30 days of school on these tests (p. 545) | Post means and SD not reported, only adjusted post means and SD from pre-test which is not obtained from the same course material (although comparable). Even though teachers and students are reasonably, balanced (table 1) the effect sizes reported without adjustment and with adjustment are quite different (0.31 versus 0.48). This is a large difference for a well-balanced randomised sample. | Intention-to-treat analysis mentioned at top of p. 10 in SOM, but results not reported. |
| Free of other bias (Judgement) | 4 | 5 | 1 |
| Free of other bias (Description) | Teacher characteristic not shown by group, only overall and further reported, "A chi-square analysis revealed no significant difference across conditions" (p. 541). Students reasonably balanced on demographic except on Special education classification (speech/language). Student pre-test (fall test) shown in table 4 but only 3 of 6 possible (one test not shown as post either). Some imbalance on one of these three pre-tests shown. Unclear if teachers participating are recruited after randomisation of schools and if all teachers (only kindergarten teachers are relevant) participate. Seems as parent consent was sought after randomisation (p. 541) and not reported how many declined, only total number of consents (in total 605 students and in total 44 teachers, giving an average of 14 students per class)) | Teacher consent obtained before randomisation but they select their “focal class” that they anticipate to be their most academically challenging class after randomisation (p. 6). Student and parent consent obtained after randomisation. Teachers and students reasonably balanced (table 1), however the baseline assessments (pre-test) were not identical to outcome assessments; i.e., they were on the course material from the prior year and 'from the most comparable course in the same subject area they took in the prior year' (p.7). It is a two-year intervention and it is not entirely clear when students used for analysis are recruited but probably at the start of year two. 'This evaluation was based upon teachers’ focal courses in the second year of the intervention.' (p. 5) and on p. 12: '.......the extension of the program across two years (as compared to a one-year, one-class intervention previously), with teachers being coached for different classes of students in the first and second years'. Students analysed in the second year may have been taught by a treated teacher in the first intervention year (and pre-tests are thus one year post-tests for some students) and teachers probably select the focal class analysed after one year of intervention. Cannot identify effect of PD on students as they only report second year outcomes and students in second year treatment (control) is an unknown mix of students who may have been treated for 1 and 2 years (unknown mix of students who may have been treated for 0 and 1 year) |  |
| A priori protocol (Judgement) | Unclear | Unclear | Unclear |
| A priori protocol (Description) | Not mentioned |  | Not mentioned. |
| A priori analysis plan (Judgement) | Unclear | Unclear | Yes |
| A priori analysis plan (Description | Not mentioned |  | Authors expected changes to accumulate over the course of the year during which teachers were exposed to the intervention and so they focused their evaluation on whether changes in student achievement would be observed in the 2nd year of the study, with a new class of students and no further coaching of the teacher. Two-year evaluation period. |
| Confounding (Judgement) | Not relevant | Not relevant | N/A |
| Notes |  | Cannot identify effect of PD on students as they only report second year outcomes and students in second year treatment (control) is an unknown mix of students who may have been treated for 1 and 2 years (unknown mix of students who may have been treated for 0 and 1 year) |  |

| Study | Ansari et al. (2018) | Babinski et al. (2018) | Bos et al., 1999 |
| --- | --- | --- | --- |
| Sequence generation (Judgement) |  | Unclear | High |
| Sequence generation (Description) |  | Matched schools on the percentage of students eligible for free or reduced-price lunch and then randomly assigned each school within a pair to either the intervention group or a waitlist/control condition. Method not reported. |  |
| Allocation concealment (Judgement) |  | Unclear | High |
| Allocation concealment (Description) |  |  |  |
| Blinding (Judgement) |  | 4 | 4 |
| Blinding (Description) |  | Nothing reported | No information concerning blinding is provided |
| Incomplete outcome data addressed (Judgement) |  | 2 | Unclear |
| Incomplete outcome data addressed (Description) |  | Forty-five teachers from 12 elementary schools within three school districts participated. One teacher left the study (not reported if the students of this teacher is included in the analysis, they state they perform ITT analysis but with no further information provided). Initially 72 students in intervention classrooms, and 46 in control classrooms. Total student attrition and missing data rate 11% evenly distributed between treated and control | Nothing is reported except number of students differs by test. No explanation of why the control group differs so much between tests. |
| Free of selective reporting (Judgement) |  | 3 | 1 |
| Free of selective reporting (Description) |  | Raw post means (and SD) not reported, only adjusted means (adjusted for student gender, age, and start-of-year overall Spanish proficiency as well as pre-intervention English proficiency on the seven subtests). They may have pre-test for all seven subscales on the WMLS-R but only report the pre-intervention Broad English score on the WMLS (thus the pre SD for each sub test is not reported). An effect size (Hedges g) is reported based on the adjusted means. Unclear if adjusting for 'pre-intervention English proficiency on the seven subtests' means adjusting for the overall Broad English score or adjusting for each of the seven pre-subtests. |  |
| Free of other bias (Judgement) |  | 3 | 1 |
| Free of other bias (Description) |  | Eligible students are Latino ELs who qualified for ESL services and spoke Spanish as their first language. Of the 154 students who met the eligibility criteria, there was imbalance on parental consent obtained; it was obtained for 72 (82% of eligible students) in intervention classrooms, and only 46 (67% of eligible students) in the control classrooms. Not reported if parent consent were sought before or after randomisation. Large imbalance on Total years of teaching for ESL teachers (appr. 1 SD) and some imbalance for classroom teachers (appr. 0.5 SD); otherwise teacher demographics reasonably balanced (table 2). Some student gender imbalance (20 percent points); otherwise student demographics and pre-test (note, pre-test not the same as post-test: The English WMLS-R has two forms; Form A was administered at the beginning of the school year and Form B at the end of the school year. The Spanish WMLS was only administered at the beginning of the year) reasonably balanced (table 3). |  |
| A priori protocol (Judgement) |  | Unclear | Unclear |
| A priori protocol (Description) |  |  | Not mentioned |
| A priori analysis plan (Judgement) |  | Unclear | Unclear |
| A priori analysis plan (Description |  |  | Not mentioned |
| Confounding (Judgement) |  | Not relevant | 5 |
| Confounding (Description) |  |  | The treatment schools are selected from the larger set of schools because student outcomes were collected there. However, it is unclear why student outcomes were collected in these schools, and by whom, so the motivation does not rule out selection. The student pre-tests are taken after the teachers get the intervention, so not pre-treatment tests. Number of classes (teachers) per school by grade not reported. There are only 19 intervention students in Kindergarten; they may well be from only one school in which case the school (and teacher) effect cannot be separated from the intervention effect. 1st grade students are largely unbalanced on one of two pre-tests, nothing is controlled for. 2nd grade students are reasonably balanced on all 3 pre-tests, nothing is controlled for. Results reported for 30 intervention students so probably two classes (teachers) involved but they may be from the same school in which case the school effect cannot be separated from the intervention effect |
| Method for identifying relevant confounders described by researchers. |  |  | None |
| Relevant confounders described |  |  | Teacher special education, gender, ethnicity, education, experience and preparation information provided (p. 230) although not by grade. Student numbers by grade provided in table 3 and pre and post means provided divided by grade. Results by grade should be used, as there is a large student imbalance on grade (and thereby for teachers). Three pre-tests reported for Kindergarten, one is unbalanced and one is reported for appr. half the control group. Two pre-tests reported for 1st grade students, one is largely unbalanced. Three pre-tests reported for 2nd grade students, all reasonably balanced. Large imbalances on the teacher measures reported in Table 1 (preparation for teaching reading), p. 230. It is not clear, however, that these measures are pre-treatment measures but perhaps most likely given the context. |
| Method used for controlling for confounding (At design state) |  |  | 11 teachers from two intervention schools and 17 teachers from two control schools with control schools chosen to be similar regarding curriculum standards and practices, student socioeconomic status and ethnicity (p. 230) |
| Method used for controlling for confounding (At analysis stage) |  |  | Nothing except grade-divided results provided. Seem to be using a repeated measure ANOVA for some analyses, so in those analyses pre-test scores ought to be controlled for. |
| Notes | Use same data as Pianta et al 2017 (scores 5) | Awaiting answer from authors, else cannot be used in meta-analysis |  |

| Study | Brownell et al. (2017) | Buysse et al., 2010 | Cabell et al., 2011 |
| --- | --- | --- | --- |
| Sequence generation (Judgement) | High | Unclear | Unclear |
| Sequence generation (Description) | Block randomise at school level (schools were ranked according to the percentage of students receiving FRL and assigned to one of two conditions, method not reported) but stated that "teachers in both conditions selected one of their reading groups to participate in the study" (p. 149) and it is not reported that the selection was done before randomisation; probably done after randomisation. | Classrooms randomly assigned. If two from same Pre-Kindergarten programme both were assigned to the same condition. Method not reported | Stratified by region, 38 preschool centres were randomly assigned to conditions. Method not reported |
| Allocation concealment (Judgement) | High | Unclear | Unclear |
| Allocation concealment (Description) |  |  |  |
| Blinding (Judgement) | 4 | 3 | 4 |
| Blinding (Description) | No indication that any participant is blind to treatment status. | Data collectors who were blind to the study conditions (p. 199) | No information concerning blinding is provided |
| Incomplete outcome data addressed (Judgement) | Unclear | 2 | 3 |
| Incomplete outcome data addressed (Description) | Nothing reported | No classroom attrition. 26/29 intervention/control classrooms and report 4 students from each are selected for analysis, they analyse 92/101 intervention/control students, thus attrition (or missing data, not reported why they do not analyse 4 from each classroom) rate 12%/13% | Overall teacher attrition rate 6% (3 teachers) not reported by group. Student attrition rate 6%. Missing data rate vary by measure, total attrition and missing data rate between 23% and 31% for intervention and 25% to 28% for control |
| Free of selective reporting (Judgement) | 1 | 1 | 1 |
| Free of selective reporting (Description) |  |  |  |
| Free of other bias (Judgement) | 5 | 1 | 3 |
| Free of other bias (Description) | No demographics shown or reported by condition, only stated that teachers were similar in terms of gender, race, degrees, certification areas, and experience and students had similar demographic characteristics. Pre-test means and SD of the standardised teacher test (several non-standardised teacher tests also reported) reported on p. 156, very large difference (0.73 SD). Some student pre-test imbalances on 4 of five pre-tests (0.17-0.24 SD) all in favour of treated (table 1). Teachers choose which students that participate, probably after randomisation. | Parent consent and random selection of eligible students (4 Latino DLLs from each classroom) done before randomisation of classrooms. Student and teacher characteristic shown in table 1, reasonably balanced. Classroom pre values of practices shown in table 2 and student pre-tests shown in table 4, all reasonably balanced | Two sequential cohorts involving a total of 49 preschool teachers were analysed, numbers or results by year not reported. Teacher characteristic not reported by group, only that There were no statistically significant differences with regard to teachers’ race/ethnicity, level of education or years of experience (p. 318). Piasta et al., 2012 report teacher characteristic by group. Teachers reasonably balanced on gender, ethnicity, education and experience (table 1 and 2 in Piasta et al., 2012) Five to eight target children from each of 49 teachers’ classrooms were randomly selected from those for whom consent was received and who met an age eligibility criterion. Not reported if parent consent and selection of children was done before or after randomisation. Student demographic in table 1, age not reported by group, otherwise no large imbalances. Pre-tests (7) reported in table 3, no large imbalances. Note that (according to Piasta et al., 2012) teachers assigned to the comparison condition received an equivalent amount of PD, albeit on topics unrelated to conversational responsiveness (the focus of the treatment PD). There is a risk that the comparison condition may have affected the child outcome measures |
| A priori protocol (Judgement) | Unclear | Unclear | Unclear |
| A priori protocol (Description) |  | Not mentioned | Not mentioned |
| A priori analysis plan (Judgement) | Unclear | Unclear | Unclear |
| A priori analysis plan (Description |  | Not mentioned | Not mentioned |
| Confounding (Judgement) | Not relevant | Not relevant | Not relevant |
| Notes |  |  | Same trial as in Piasta et al., 2012. Cabell et al., 2011 reports on student outcomes and Piasta et al., 2012 reports on students (but transcribed from video only, another outcome than reported in Cabell et al., 2011) |

| Study | Dickinson & Caswell, 2007 | Early et al., 2017 | Gallagher et al., 2011 |
| --- | --- | --- | --- |
| Sequence generation (Judgement) | High | High | Unclear |
| Sequence generation (Description) |  | Method not described. P. 61 (2 and 3 year assignment) Classes, rather than teachers, were selected for participation because often teachers were not assigned to classrooms until very close to the start of the academic year and occasionally teachers were not assigned until after the school year had begun. Thus, the final step in the random selection and assignment process involved learning which teacher was assigned to the selected classroom, determining if she or he was eligible for participation, and replacing any classes where the teacher was ineligible. This is not proper randomisation, randomisation occurs too soon. Not sure it could/should be classified as a RCT for these two years (and as they do not show results separate for the first year this concerns the whole trial) | Head Start program teacher (mentor) and protégé pairs randomly assigned, method not reported |
| Allocation concealment (Judgement) | High | High | Unclear |
| Allocation concealment (Description) |  |  |  |
| Blinding (Judgement) | 3 | 2 | 4 |
| Blinding (Description) | Data collection was conducted by research assistants who were not connected to the delivery of the intervention and were blind to condition (p. 248) | Independent data collectors conducted CLASS observations at start and end of school year. Different data collectors for fall and spring obs. P. 62-63. Data collectors were unaware of project design, blind to teacher's PD condition. | No information concerning blinding is provided and in addition some of the student outcomes are teacher rated |
| Incomplete outcome data addressed (Judgement) | 3 | 2 | 5 |
| Incomplete outcome data addressed (Description) | Missing data 11% for three outcomes and 4% for one outcome, not reported by group and not reported why data is missing | 27 teachers, or 5.3% of the original sample, left the study between the pre- and post-test. Of these, 8 had been assigned to MMCI (4.4% of the original MMCI sample); 8 to MTP (5.0% of the original MTP sample), and 11 to control (6.4% of the original control sample). Thus, the differential attrition rate was 2.0% (6.4 minus 4.4). p. 61 Low attrition. Reasons for attrition are stated at p. 61 (most stopped teaching in Georgia’s Pre-K during the year) and further it is stated that no differences were found in any of the three CLASS domains on pre-test scores, results not shown though. | Students from 8 treatment and 8 control classrooms participate and no overall number of students is reported. The 13 FACES outcomes (collected by outside assessors) are only collected for 2 treatment and 2 control classrooms (state that they are randomly selected). Actual number of students is 24-30 for treated and 14-18 for control (table 5). It is not reported how many students were in the classes selected for data collection. State on p. 65: "In addition, the researchers attempted to obtain teacher assessments for all children in all of the treatment and control Protégé classrooms". For the PLBS outcome (4 items and a total score) the number of students rated vary between 122 and 124 for treated and between 86 and 89 for control (table 6). For the LELA outcome (6 items and a totals core) the number of students rated vary between 91 and 125 for treated and between 56 and 61 for control (table 7). It is not stated how children within classroom are selected. |
| Free of selective reporting (Judgement) | 4 | 3 | 1 |
| Free of selective reporting (Description) | Two subscales from the Assessment Profile (Abbott-Shim & Sibley, 1998), Learning Environment and Interacting, were used but only Learning Environment is shown (authors state that there was too little variation in the Interacting subscale so it was dropped from analysis but not even means from this subscale is shown). | No descriptive of attritors and results on differences in pre-test scores not shown (only mentioned there were no differences). A sensitivity check in which the main impact analyses were repeated including only the teachers in the second and third year of the project. Not all results reported and results separated by each year should have been reported. |  |
| Free of other bias (Judgement) | 3 | 2 | 5 |
| Free of other bias (Description) | Three ELLCO components are used as outcome measures and LEEP is the intervention investigated. On p. 248, it is stated: "The ELLCO was developed by the same team of researchers that created LEEP, but it was a separate endeavour. ELLCO reflected the same theoretical understanding of literacy that was communicated to teachers taking LEEP, but it was not specifically tailored to assess the effects of LEEP on classroom practices." | Teacher demographics and pre-test reasonably balanced (table 1 and 2). P. 64: In the first year of the study, teachers in the control group (n = 51) had access to the same online library of video clips demonstrating best practices in various aspects of teacher-child interactions as the MMCI and MTP teachers. No data are available regarding how much those teachers accessed the library, but anecdotal evidence suggested that it was used very little. In the second and third years, teachers in the control group (n = 109) participated in the same 15 h of professional development required of all Georgia’s Pre-K teachers. Topics varied, but included behaviour management, child assessment, outdoor learning, and others. Teachers in the control group did not receive direct training related to the CLASS, although some of the professional development opportunities may have been aligned with CLASS concepts. | Mentor and protégé age, education and years of experience (total and Head start programme) shown in tables 1 and 2. Some imbalance on all variables. Teacher beliefs, efficacy and policy and program management variables also shown and they are reasonably balanced. However, many imbalances are very large in Table 1 and 2, p. 62 and 63. Some approach or is over a standard deviation (e.g., "Appropriate beliefs" in Table 1 and "Years of education" in Table 2). Several outcomes are reported by the teachers themselves. No student demographic characteristic reported. Some child outcomes are only measured in 4 randomly selected classrooms (2 treatment and 2 controls), the imbalances on the pre-tests shown in Table 5 (p. 68) are mostly small, but three are substantial (two around 0.3 and one almost 0.7). All are small in Table 6 (p. 69), and most are small in Table 7 (p. 7), although the total scores difference is over 0.3 SD. Differences are both in favour of the control and treatment group. Nothing is controlled for in the analysis. |
| A priori protocol (Judgement) | Unclear | Unclear | Unclear |
| A priori protocol (Description) | Not mentioned | Not mentioned. | Not mentioned |
| A priori analysis plan (Judgement) | Unclear | Unclear | Unclear |
| A priori analysis plan (Description | Not mentioned | Not mentioned. | Not mentioned |
| Confounding (Judgement) | 5 | Not relevant | Not relevant |
| Confounding (Description) | Teacher age and gender not considered some imbalance on teacher education and two of four pre values of outcome measures. Teacher ethnicity, experience, education and relevant pre value controlled for. No student characteristic considered. Unclear if interaction terms are included in the analyses |  |  |
| Method for identifying relevant confounders described by researchers. | None |  |  |
| Relevant confounders described | Teacher ethnicity, experience and education considered (text p. 247), some imbalance on education. Gender and age not considered. Pre values of outcome measures reported in table 1 p. 251. Some imbalance on two of four summary measures. No student characteristic considered. |  |  |
| Method used for controlling for confounding (At design state) | Participants are teachers recruited from Head Start programs over a 2-year period. Treated were those recommended by program directors to attend the LEEP course (the intervention). Control teachers In the first year, were identified by program directors as being equivalent to treated teachers in terms of prior experience and overall classroom instructional skills. Still quite dramatic selection, especially in the first year. Second year is more unclear, why are some teachers not granted admission? Do not understand the author claim: "The use of a wait list serves to diminish selection bias because it can be assumed that comparison teachers are similar to treatment teachers on unobserved variables such as motivation and interest" (p. 247). Unclear why the waitlist would achieve this? |  |  |
| Method used for controlling for confounding (At analysis stage) | Regression (the authors term call Hierarchical regression, but no levels are involved so it is ordinary linear regression). Unclear if interaction term are included, the authors state that they "tested for interactions between significant predictors and background variables in all models" (p. 250) and an interaction term between intervention and ethnicity is shown in one of the results tables (not in the other two) but no coefficient is shown (table 2). They use only the pre-test corresponding to the post-test as control in each regression. Unclear why not all pre-tests are used. |  |  |
| Notes | Author has developed the intervention, p. 246. |  |  |

| Study | Garet et al., 2008 | Gersten et al. (2010) | Hamre et al. (2012) |
| --- | --- | --- | --- |
| Sequence generation (Judgement) | Unclear | Unclear |  |
| Sequence generation (Description) | Within each district, schools were randomly assigned in equal numbers to treatment A, treatment B, or the control group. In five districts, schools were grouped into blocks of schools with similar characteristics and randomised within blocks (p. 10). Method not reported | Schools within districts (3) and years (2) were randomly assigned to condition (p. 15) but method not reported. In two districts, schools were matched before randomisation (probably in pairs but not reported). In one district 'API (Annual Performance Index) scores, ethnic composition (percentage Hispanic), and achievement scores' were used and in another district 'free/reduced lunch status and reading proficiency on the 3rd grade state-wide assessment test' were used. Students were randomly selected from each class. |  |
| Allocation concealment (Judgement) | Unclear | Unclear |  |
| Allocation concealment (Description) |  |  |  |
| Blinding (Judgement) | 4 | 4 |  |
| Blinding (Description) | No information concerning blinding is provided and in addition some of the student outcomes are teacher rated | Classroom observers (for teacher measures) were blind to teacher assignment to treatment condition. Not reported if student test administers or assessors were blind to condition. |  |
| Incomplete outcome data addressed (Judgement) | 3 | 2 |  |
| Incomplete outcome data addressed (Description) | Six districts, 90 schools, and 270 second grade teachers participated in the study during the year that the PD interventions were implemented. By the end of intervention 270 teachers participated and 4.4% of these were 'early entrants’ replacing teachers who had left during the year (6.5% treatment A, 4.6% treatment B and 2.2% for control). During the follow-up year (which included only data collection), the number of teachers participating was 250 in the fall and 254 in the spring and overall retention rates (from beginning of experiment) was 71.6% and 67.3% (p. 12-13). 17 percent of students who were enrolled by the end of treatment had not been enrolled from the beginning of the school year and 27% had left the schools. The corresponding information for the follow-up year is unavailable because one district did not provide follow-up year student attendance data. (p. 13). Overall outcome data is available for 91 and 87 percent of the eligible second grade students attending the study schools by the end of intervention and in the follow up year (p. 16). Student outcome data not available for one control school (p. 50) | 10 treatment schools and 9 control schools. 40 treatment teachers (1 teacher, 3%, dropped out), 44 control teachers (2 teachers, 5%, dropped out). 273 treatment students (56 students, 21%, dropped out), 302 control students (51 students, 17% dropped out), primarily due to family relocation. |  |
| Free of selective reporting (Judgement) | 1 | 3 |  |
| Free of selective reporting (Description) | Separate impact estimates for each study district were averaged to an overall weighted impact estimate by using the number of schools in each district’s sample as weights. Effect estimates without covariates shown in Table L-3. Effect estimates by district shown in Figure L-7 | Pre-test means and standard deviations not reported on student level, only on school level. Post means and standard deviations not reported, only results from multilevel models with measures standardized to have a mean of zero and standard deviation of one. Unclear how they standardize their outcome variables (what standard deviation they use).Report only effects at the school level. |  |
| Free of other bias (Judgement) | 2 | 3 |  |
| Free of other bias (Description) | Individual-level pre-test scores in reading were not available for the second grade students in the study. Second grade student test score data for the two years before intervention (2004 and 2005) were aggregated to the school level and used as covariates. In addition, student-level demographic information on gender, age, race/ethnicity, and a separate poverty measure for each district were included as covariates (p. 25). Tables 2-7 and 2-8 show school and teacher characteristics at the time of random assignment. Reasonably balanced except student ethnicity | Two trials: In Year 1 (2004-2005) the study was conducted in a school district in California. In year 2 (2005-2006) the study was replicated in school districts in Pennsylvania and Virginia; additional schools in the California site participated. No data shown separately for the two trials. Some imbalance on teacher education, otherwise teacher demographics reasonably balanced (table 1). Student gender and language minority reasonably balanced (table 2). Pre-test shown at school level, uncertain if only the analytic sample of students are included or all students. Four pre-tests reasonably balanced, some (0.25-0.35 SD) imbalance on two pre-tests, table 3. Pre-test and post-test measures are not the same and estimate effects using only school means of one of the pre-tests as covariate (in level two, the school) |  |
| A priori protocol (Judgement) | Unclear | Unclear |  |
| A priori protocol (Description) | Not mentioned |  |  |
| A priori analysis plan (Judgement) | Unclear | Unclear |  |
| A priori analysis plan (Description | Not mentioned |  |  |
| Confounding (Judgement) | Not relevant | Not relevant |  |
| Notes |  | Analysis on school level, no data on individual level | Uses same data as Pianta 2017 but only phase 1 data and unlike the other two, they report teacher (CLASS) outcomes. Scores 5 (assessment the same as Sandilos) |

| Study | Hindman & Wasik, 2012 | Howlin et al., 2007 | Jayanthi et al. (2018) |
| --- | --- | --- | --- |
| Sequence generation (Judgement) | High | Low | Unclear |
| Sequence generation (Description) | Head Start (HS) centres were randomly assigned to conditions, however seven centres volunteered to participate but only one centre was randomly assigned to the control condition and two were randomly selected to participate in the intervention (p. 5) | Classes were stratified according to size and in each stratum, randomly allocated to one of the three conditions using an online randomisation programme (http://www.random.org) | In 16 districts in four states, 62 schools were randomised within districts, method not reported. Randomly select 85.5% of teachers for participation and 10 students were randomly selected from each class |
| Allocation concealment (Judgement) | High | Low | Unclear |
| Allocation concealment (Description) |  | Non-sequential and therefore low risk of bias |  |
| Blinding (Judgement) | Not relevant | 4 | 4 |
| Blinding (Description) |  | Outcome assessors not blind to condition (p. 476) |  |
| Incomplete outcome data addressed (Judgement) | Not relevant | 2 | 2 |
| Incomplete outcome data addressed (Description) |  | Attrition: 1 class (of 6 that is 17%) in Treatment 1 and none in treatment 2 (delayed treatment) and control. Some (low) attrition at student level, but ITT analytic strategy used | School attrition rate 1.6% (one school of 62) and teacher attrition rate 4.7% (9 of 191), differential attrition D 0.7%. Student attrition rate 7.2%, differential attrition of 2.0%. |
| Free of selective reporting (Judgement) | Not relevant | 4 | 3 |
| Free of selective reporting (Description) |  | Raw means not reported, only the final OR (raw means are recoded into ordinal categories (0; 1 to 20; >20) and ordinal regression model used) | No raw means and standard deviations for the post-test is reported, only results from multilevel models with measures standardized to have a mean of zero and standard deviation of one. Unclear how they standardize their outcome variables (what standard deviation they use).Report only effects at the school level. |
| Free of other bias (Judgement) | 5 | 4 | 2 |
| Free of other bias (Description) | One Head Start centre is control and two centres are treated. Cannot separate centre from treatment effect | Students mean age shown in table 1, some imbalance. Baseline measures of symptom severity (ADOS-G) and non-verbal developmental quotient (NVDQ) are imbalanced but no difference in ADOS total score (text p. 477). Otherwise no demographic or pre-tests considered. | Small school imbalances (table 2). Teachers reasonably balanced (text p. 4 and 5). Small gender, ethnicity and Limited English Proficiency imbalances on students, but pre-tests reasonably balanced (table 3) |
| A priori protocol (Judgement) | Not relevant | Yes | Unclear |
| A priori protocol (Description) |  | The original trial protocol was approved by the Wandsworth Local Research Ethics Committee (Ref. IAS/der/02.42.6). |  |
| A priori analysis plan (Judgement) | Not relevant | Unclear | Unclear |
| A priori analysis plan (Description |  |  |  |
| Confounding (Judgement) | Not relevant | Not relevant | Not relevant |

| Study | Johnson et al. (2017) | Kammermeyer et al., 2016 | Landry et al., 2009 |
| --- | --- | --- | --- |
| Sequence generation (Judgement) | Low | Unclear | Unclear |
| Sequence generation (Description) | Four schools were randomly assigned to treatment and control conditions via a random number generator | Classes randomly assigned to one of 3 conditions. Method not reported | Across 4 states, 158 schools (262 classrooms) were randomly assigned to one of 5 conditions. One state participated in another year than the others. Method of randomisation not reported |
| Allocation concealment (Judgement) | Low | Unclear | Unclear |
| Allocation concealment (Description) |  |  |  |
| Blinding (Judgement) | 3 | 4 | 4 |
| Blinding (Description) | Trained research assistants who were blind to condition collected baseline observations of classrooms, not explicitly mentioned that they were blind to condition when collecting post observations but most likely they were blinded | No information concerning blinding is provided | No information concerning blinding is provided. For teacher outcomes, observers are said to be unaware of treatment status (p. 453). It is unclear whether this is the case also for child outcomes. |
| Incomplete outcome data addressed (Judgement) | 1 | 3 | Unclear |
| Incomplete outcome data addressed (Description) | There were no missing data or attrition | Attrition rates (students): Treatment 1: 13.0%, Treatment 2: 6.1%, control: 23.9% (table 1) | Total 1,786 students and 262 classrooms. Not reported by group. Only reported that number of students with both pre and post-tests varied between 1,607 and 1,678. |
| Free of selective reporting (Judgement) | 2 | 1 | 5 |
| Free of selective reporting (Description) | Teacher demographics not reported by group |  | Means and SD not reported, means (with what is probably CIs) only shown in unclear figure where values cannot be read and only for two outcomes. Otherwise, only a variety of F-values (conditions, site interaction, and pre-test interaction) and sum of two by two treatment groups vs control t-values but only significant values are reported. |
| Free of other bias (Judgement) | 3 | 2 | Unclear |
| Free of other bias (Description) | It is reported that there were no significant differences on any teacher or classroom demographic variables between the two conditions but data not shown. Pre-test shown in table 1, imbalances on all three summary measures ranging from 0.12 to 0.51 SD. Baseline measures likely collected after randomization (otherwise collectors would always be blind to condition, so shouldn't be mentioned p. 463). | Student characteristic and teacher gender shown in table 1. Reasonably balanced except between treatment 2 (DAA) and control on student gender and language. Four pre-tests shown, small imbalance on one (both treatments). | Up to eight children with parent consent were randomly selected from each classroom to participate. Not reported if parent consent was sought before or after randomisation. No information by group provided, not even number of classes or students by group. Number of teachers could perhaps be estimated from the degrees of freedom in the respective t-tests. However, unclear if they are from the ANCOVAs. Seems not as some have 69 degrees of freedom, which is the largest number of teachers included in any analysis according to p. 454. This is also weird, because should always be less than 69 dgf then. The numbers could similarly be checked for students. However, this does not reveal the number of sites. The ANCOVAs are not reported in any table. |
| A priori protocol (Judgement) | Unclear | Unclear | Unclear |
| A priori protocol (Description) |  | Not mentioned | Not mentioned |
| A priori analysis plan (Judgement) | Unclear | Unclear | Unclear |
| A priori analysis plan (Description |  | Not mentioned | Not clearly mentioned. They mention that "a priori contrasts had been specified" (p. 454), but provide no supporting evidence or a pre-registered analysis plan. Unclear also whether the model used for testing was pre-specified. |
| Confounding (Judgement) | Not relevant | Not relevant | Not relevant |
| Notes |  | There is a third treatment but kindergartens not randomised and they are in several different states (not Rhineland-Palatinate) whereas the randomised kindergartens are all located in Rhineland-Palatinate (and further required to be at a distance of less than 350 km to the University of Landau). Cannot separate state effect from treatment effect |  |

| Study | McCollum et al., 2013 | Murphy et al. (2017) | Neuman & Cunningham, 2009 |
| --- | --- | --- | --- |
| Sequence generation (Judgement) | Unclear | High | Low |
| Sequence generation (Description) | Teachers in 13 classrooms, representing three different state-funded prekindergarten programs within one county, were randomly assigned within programs. Method not reported | Schools were stratified by Local Authority and were pairwise randomly assigned (although a bit differently for round 1 and 2 of the randomisation, see p. 13-14). Randomization was done in office by a computer setting different seeds and each school was then given a random number from a uniform distribution. Schools that were selected into the treatment group were asked to select three teachers to be part of the trial. Schools had complete autonomy over which teachers they chose, on the condition that two of the selected teachers were to teach Years 4 and 5. The second round of randomisation was done because 16 of the schools initially randomised to treatment declined participation after randomisation | Providers from 304 centres (one from each, 168 centres, 136 homes) were stratified by centre- and home-based setting and randomly assigned (using a table of random numbers) to one of three conditions |
| Allocation concealment (Judgement) | Unclear | High | Low |
| Allocation concealment (Description) |  |  | Non-sequential and therefore low risk of bias |
| Blinding (Judgement) | 4 | 3 | 4 |
| Blinding (Description) | No information concerning blinding is provided | The study relies entirely on linked-in administrative data that is available for all students in state education in England | No information concerning blinding is provided |
| Incomplete outcome data addressed (Judgement) | 3 | 4 | 2 |
| Incomplete outcome data addressed (Description) | Attrition teachers: Treated 0% and control 17% | Initially 83 schools allocated to treatment, 16 decline participation after randomisation (20%), a further 6 randomised to treatment, 5 drop out first year (7%) and 4 drop out second year (5%). No control schools drop out. Only report demographics and raw means for the ITT sample (all 89 'treated' schools and all of their 4 and 5 year students regardless of whether their teacher was treated). The highest missing student data rate is less than 5 per thousand: 13 treatment and 0 control pupils in the first cohort had no measure, and 1 treatment and 5 control pupils in the second cohort had no measure. Scoring concerns the ATE (LATE), the ITT scores 2 | Attrition teachers (providers): Treatment 1: 7%, treatment 2: 0%, control: 5% |
| Free of selective reporting (Judgement) | 1 | 3 | 1 |
| Free of selective reporting (Description) |  | Perform an ITT and an ATE (the authors term it an ATE, actually it is a LATE as they perform an IV analysis using randomisation as instrument) analysis but number of students used for analysis only reported for the ITT analysis. |  |
| Free of other bias (Judgement) | 5 | 5 | 3 |
| Free of other bias (Description) | Teachers in 13 classrooms, representing three different state-funded prekindergarten programs. One program had three teachers in the intervention group and four in the control group, the second had two teachers in each group, and the third had one teacher in each group of which the control teacher later dropped out of the study. Teachers reasonably balanced on education, experience and gender (text p. 30). Ethnicity is unclear, as it is not reported by group. Student characteristic not reported by group, only significance test values and only age, ethnicity and Individualized Education Programs is reported. Pre values of teacher outcomes reported in table 3, large imbalances (SMD between 0.55 and 0.67) on all and all favouring treated. There are also large pre-test imbalances on 2 out of 3 measures in Table 2 (p. 33), which documents percentage of skills used in early literacy teaching. They favour the treatment group. Furthermore, the relative increase in these skills that were targeted by the intervention is larger for Cluster A in the control group. The nonparametric tests they use in the analysis does not control for any pre-treatment differences. | Schools and students reasonably balanced (table 4.1) but note this is the ITT sample which may be very different from the ATE (authors term it ATE, actually it is a LATE using randomisation as instrument) sample (both concerning schools and concerning students). No teacher demographics reported. There is evidence that some control schools implemented similar approaches to Lesson Study, such as teacher observation. Not all participating students' teachers are treated (in the ITT sample) but the number is not reported, it is stated that student participants were 'pupils who were in Years 4 and 5 in the first year of the trial’. Three teachers per school were treated and: Schools were free to choose which teachers would be involved in the intervention, with the restriction that two should be teaching year groups 4 and 5. (p. 7). P. 14: 'Because we could not identify students to teachers, the ideal was to have only one class per cohort. However, in reality many schools had two classes per year group.' (see also p. 17). For 69 schools they do however have a list of which students were actually treated (note in some schools it may actually be 0% of a given cohort) but the number is not reported. Perform an ITT and ATE (LATE) analysis. The scoring of 5 concerns the ATE (LATE) only, the ITT scores 4 | The control-group participants were more educated, had taken more education courses, and were slightly older whereas ethnicity, gender, and years of experience were similar across groups. (Table 1). Pre values for overall score in table 5 p. 555, no large imbalance (subscales are shown in tables 3 and 4) |
| A priori protocol (Judgement) | Unclear | Yes | Unclear |
| A priori protocol (Description) | Not mentioned | This trial was registered with AEA RCT Registry and assigned registry number AEARCTR-0001779. | Not mentioned |
| A priori analysis plan (Judgement) | Unclear | Yes | Unclear |
| A priori analysis plan (Description | Not mentioned | No analysis plan in the AEARCTR-0001779, but it is stated that both the primary analysis and the protocol analysis was undertaken (with an explanation of what they are) | Not mentioned |
| Confounding (Judgement) | Not relevant | Not relevant | Not relevant |
| Notes |  | Note different scoring on two items for the ITT and the ATE (LATE) |  |

| Study | Olson et al. (2017) Year one | Olson et al. (2017). Year two | Ottmar et al., 2013 |
| --- | --- | --- | --- |
| Sequence generation (Judgement) | High | High | Low |
| Sequence generation (Description) | Teachers (from 16 schools) were randomly assigned separately for each grade within each school and one of each teacher’s classes was randomly selected to participate (however later it is stated that; *the class selected was the one that had the greatest percentage of ELs, and in which the students had the English language proficiency necessary to write in English and thereby profit from the enhanced training received by the teachers who participated in the program', so probably not random). Methods of random assignment not reported |  | Schools are matched and randomised. Method reported in Rimm-Kaufman, 2014 (using the random number function in Excel) |
| Allocation concealment (Judgement) | High | High | Low |
| Allocation concealment (Description) |  |  |  |
| Blinding (Judgement) | 3 | 3 | 3 |
| Blinding (Description) | Teachers within the same schools are assigned to treatment and control after consent, so not blind to treatment status. Parents and students might have been. Test is a statewide exam, so tester is blind to treatment status. | Teachers within the same schools are assigned to treatment and control after consent, so not blind to treatment status. Parents and students might have been. Test is a statewide exam, so tester is blind to treatment status. | Videos of observations were coded blindly (sent away to coders who were blind to assignment) p. 442 |
| Incomplete outcome data addressed (Judgement) | 2 | 4 | 2 |
| Incomplete outcome data addressed (Description) | "There was teacher attrition between the years (Pathway teachers declined from 49 to 41 and controls from 46 to 40), and this altered the demographics of their students (where the Pathway students were receiving the program for the first time and the controls had never experienced the program)." (p. 5-6). The attrition in Year 2 yields several imbalances on student characteristics. There is some incomplete outcome data also in Year 1 (some concerns the writing test, which we should not use); they use only the CAHSEE, which is taken by 10th graders. Not all take this test either, but comparing shares/numbers in table 1 & 2 with table 6 & 7 attrition seem small (8% treated and 12% control year one; 7% control and 8% treated year two). Year 1 = 2 and Year 2 = 4. | "There was teacher attrition between the years (Pathway teachers declined from 49 to 41 and controls from 46 to 40), and this altered the demographics of their students (where the Pathway students were receiving the program for the first time and the controls had never experienced the program)." (p. 5-6). The attrition in Year 2 yields several imbalances on student characteristics. There is some incomplete outcome data also in Year 1 (some concerns the writing test, which we should not use); they use only the CAHSEE, which is taken by 10th graders. Not all take this test either, but comparing shares/numbers in table 1 & 2 with table 6 & 7 attrition seem small (8% treated and 12% control year one; 7% control and 8% treated year two). Year 1 = 2 and Year 2 = 4. | Not attrition per se - 100 teacher approached and 94 consented to participate. Attrition is present at follow up - Table 2 p. 447 (results) - teachers n = 88 (94 teachers in baseline sample) - reasons not given for this attrition. Stated p. 441 that an additional six teachers were excluded because they taught mathematics in a foreign language. |
| Free of selective reporting (Judgement) | 1 | 1 | 3 |
| Free of selective reporting (Description) |  |  | Descriptive statistics not shown other than overall means (treated and control pooled) and correlated with treatment status. Reports only results from the first of three years (it was a three-year intervention) with no explanation. |
| Free of other bias (Judgement) | 2 | 4 | 1 |
| Free of other bias (Description) | Sample is well balanced on two teacher characteristics (p. 5), and on overall 1st year student characteristics, including pre-tests (p. 6). Second year is less well balanced overall due to attrition. Stated on p. 12 concerning second year of intervention: 'To be clear, Pathway students in this sample are in their first year experiencing the program, but Pathway teachers are in their second year with the program. Control students and teachers have never experienced the program.' Thus all teachers (at least treated teachers) have classes with completely different students than year one and control teachers do not have year one treated students in their classes. This implies that the allocation of students to teachers cannot have been completely random in year two. Only the outcome California High School Exit Exam (CAHSEE) is relevant for this review and only reported for 10th grade. Teacher characteristics not reported by group other than p-values (no significant differences) and not separately for 10th grade teachers, which are the only relevant teachers for this review. Overall students reasonably balanced (year 1, table 1) and small imbalances calculated from table 7, 10th grade students, small overall imbalances in year two (table 2) and some imbalances calculated from table 8, 10th grade students. | Sample is well balanced on two teacher characteristics (p. 5), and on overall 1st year student characteristics, including pre-tests (p. 6). Second year is less well balanced overall due to attrition. Stated on p. 12 concerning second year of intervention: 'To be clear, Pathway students in this sample are in their first year experiencing the program, but Pathway teachers are in their second year with the program. Control students and teachers have never experienced the program.' Thus all teachers (at least treated teachers) have classes with completely different students than year one and control teachers do not have year one treated students in their classes. This implies that the allocation of students to teachers cannot have been completely random in year two. Only the outcome California High School Exit Exam (CAHSEE) is relevant for this review and only reported for 10th grade. Teacher characteristics not reported by group other than p-values (no significant differences) and not separately for 10th grade teachers, which are the only relevant teachers for this review. Overall students reasonably balanced (year 1, table 1) and small imbalances calculated from table 7, 10th grade students, small overall imbalances in year two (table 2) and some imbalances calculated from table 8, 10th grade students. |  |
| A priori protocol (Judgement) | Unclear | Unclear | Unclear |
| A priori protocol (Description) |  |  | Not mentioned |
| A priori analysis plan (Judgement) | Unclear | Unclear | Unclear |
| A priori analysis plan (Description |  |  | Not mentioned |
| Confounding (Judgement) | 3 | 4 | Not relevant |
| Confounding (Description) | It is stated but not shown (by group means) that sample is well balanced on teacher characteristics (only years of teaching and education mentioned) in year one (p. 5), and on 1st year student characteristics, including pre-tests (both the AWA which is a measure developed for the Pathway Project and the CST for English Language Arts (CST-ELA) which is probably a standardized test) (table 1). Second year students are less well-balanced (table 2), the author’s state it is due to differential teacher attrition across schools with different demographics (p. 6). Note, the CST for English Language Arts (CST-ELA) is not reported for year two students. It is not reported if this teacher attrition also implied imbalance on teacher characteristics in year two. | It is stated but not shown (by group means) that sample is well balanced on teacher characteristics (only years of teaching and education mentioned) in year one (p. 5), and on 1st year student characteristics, including pre-tests (both the AWA which is a measure developed for the Pathway Project and the CST for English Language Arts (CST-ELA) which is probably a standardized test) (table 1). Second year students are less well-balanced (table 2), the author’s state it is due to differential teacher attrition across schools with different demographics (p. 6). Note, the CST for English Language Arts (CST-ELA) is not reported for year two students. It is not reported if this teacher attrition also implied imbalance on teacher characteristics in year two. |  |
| Method for identifying relevant confounders described by researchers. | No | No |  |
| Relevant confounders described | Yes, student gender, race, grade, language proficiency status, and free/reduced lunch eligibility and for year one only the CST for English Language Arts (CST-ELA) test. California elected not to administer the California Standards Test (CST) in 2014 but summer 2013 is the time of pre-test for year two. Pre-test is not controlled for. | Yes, student gender, race, grade, language proficiency status, and free/reduced lunch eligibility and for year one only the CST for English Language Arts (CST-ELA) test. California elected not to administer the California Standards Test (CST) in 2014 but summer 2013 is the time of pre-test for year two. Pre-test is not controlled for. |  |
| Method used for controlling for confounding (At design state) | Randomize teachers to treatment, and then non-randomly pick a class from each teacher. See Other bias for imbalances. | Randomize teachers to treatment, and then non-randomly pick a class from each teacher. See Other bias for imbalances. |  |
| Method used for controlling for confounding (At analysis stage) | For the CAHSEE (which is the test we can use) they run logistic regressions with a treatment indicator plus controls as independent variables. Note that there does not seem to be a pre-test variable included (although language proficiency is included) when the CAHSEE is used as outcome variable. Specification of outcome variable is a bit unclear, not sure if it contrasts those who passed and failed (which seems most likely given how it is presented in tables), or those who passed with those who failed and those who didn't take the exam yet. | For the CAHSEE (which is the test we can use) they run logistic regressions with a treatment indicator plus controls as independent variables. Note that there does not seem to be a pre-test variable included (although language proficiency is included) when the CAHSEE is used as outcome variable. Specification of outcome variable is a bit unclear, not sure if it contrasts those who passed and failed (which seems most likely given how it is presented in tables), or those who passed with those who failed and those who didn't take the exam yet. |  |
| Notes | Year 1 | Year 2 | Same trial as Rimm-Kaufman 2014, although reports only results (on teacher outcomes) from the first of three years (it was a three year intervention) whereas Rimm-Kaufman et al., 2014 reports student outcomes for all years |

| Study | Parkinson et al. (2015) | Pianta et al. (2017) | Piasta et al., 2012 |
| --- | --- | --- | --- |
| Sequence generation (Judgement) | Unclear | Unclear | Unclear |
| Sequence generation (Description) | Random assignment of schools within four districts. In three of the districts, schools were grouped into two or three blocks of schools with similar characteristics and randomly assigned within blocks. Method not reported | Teachers were assigned to treatment(s) randomly within site, method not reported. Students within classes randomly selected but not followed strictly and some selection may have occurred and some (selected students) are excluded from analysis (see Other bias) | 49 teachers randomly assigned, blocked by centre, method not reported |
| Allocation concealment (Judgement) | Unclear | Unclear | Unclear |
| Allocation concealment (Description) | Random assignment (of schools) was completed in the fall of 2010, one year before CLI implementation began in the study schools. |  |  |
| Blinding (Judgement) | 4 | 3 | 4 |
| Blinding (Description) | Nothing reported | Data collectors were blind to the treatment assignment | No information concerning blinding is provided. |
| Incomplete outcome data addressed (Judgement) | Unclear | 5 | Unclear |
| Incomplete outcome data addressed (Description) | Four schools (two in each condition) withdraw, thus school attrition rate is 5%. All teachers and students (in the relevant grades) who were present at the schools at the assessment times were included, thus no teacher and student attrition by construction (analyses include all students). There is missing data however: "the study team assessed more than 80 percent of eligible children each year." (p. 12). Regarding teacher outcomes: "The team randomly selected one CLI teacher and one control teacher from each kindergarten and first-grade classroom to be observed in spring 2013 (end of year 2), for a total of 150 teachers. Of those, 130 teachers (including 65 in the CLI program group and 65 in the control group) were observed and included in the impact analysis." (p. 12). No information on the total number of kindergarten and first grade teachers and no explanation as to why not all 150 teachers were included in the analysis | Three phases; Of the 427 teachers who started the course phase (phase 1), 70 (16%) dropped prior to the coaching phase (phase 2) for a variety of reasons, including moving, receiving a teaching assignment in a different grade, and so on (p. 959). At the time of randomization into the coaching phase (phase 2), 357 teachers met the conditions of having completed the course phase, agreeing to re-randomisation, and continuation into the coaching phase; 332 were randomized into the coaching treatment or the coaching phase control group in the subsequent academic year; no explanation of why not all were randomised. Prior to phase 2 73 new teachers were recruited but only 69 of these were randomised; no explanation of why not all were randomised. Of the 401 teachers participating at the beginning of the coaching phase, 76 (19%) were excluded because of lack of child outcome data. Further, the authors state: 'It is apparent from these comparisons that the classrooms for which evaluation results could not be included in analyses reflected risk factors that would have made lower scores on readiness assessments more likely.' (p. 960). Of the 401 teachers randomized in the coaching phase, 222 were available for the follow-up data collection. Teacher attrition was in large part a function of moving, being assigned to another grade, and leaving the profession of teaching (p.960). Attrition rate for end of phase 2 is 42% for those in treatment in phase 1 and 35% for those in control in phase 1 (for the new recruited sample it was overall 9%). **Overall attrition rate for phase 3 is 55%, too high risk of bias to be used in analysis.** By construction no missing student data. **Teacher and classroom outcome (only assessed in phase 2) missing data is 26% for phase 2 treated and 50% for phase 2 control; too high risk of bias to be used in analysis.** | Nothing reported |
| Free of selective reporting (Judgement) | 4 | Not relevant | 4 |
| Free of selective reporting (Description) | Not reported how many teachers (or classrooms) participated, neither in total nor by condition. Raw pre or post-test results not reported, only limited results (overall effect size and p-value) from a two-level hierarchical model with schools as second level and including students baseline achievement and percent ELLs in the school estimated within each of the four districts and weighting each districts estimate in proportion to the number of control schools in the study sample. Further: "Randomization block fixed effects were also included in the model. In cases with missing covariate measures, the missing data were replaced with district means, and a dichotomous variable indicating the missing status of a given covariate for each observation was added to the impact analysis model." (footnote 11). Regarding teachers, overall results (ES and p-value) from: "The Impact model included randomization blocks as fixed effects and school-average percentage of students identified as ELL. Impact estimates were calculated for each district within a single model, and the overall difference between the CLI and control group was computed as a precision-weighted average across the four districts." (footnote 7) | For the post intervention follow-up year assessments, the procedure was the same except children were excluded from eligibility if they had been enrolled in that teacher’s classroom in the prior-year coaching phase (but what about treated last year children now in a control teachers class?? Cannot identify effect for children) | Students were assessed on direct measures in the fall and spring of the academic year to study project impacts, these measures (Core Language Composite of the Clinical Evaluation of Language Fundamentals Preschool-2 (CELF-P:2; Wiig, Secord, & Semel, 2004) and the Peabody Picture Vocabulary Test-III (PPVT III; Dunn & Dunn, 1997)) are not reported here but in Cabell et al., 2011. Only student measure reported are linguistic productivity and complexity during small-group interactions with their teachers measures transcribed from videos submitted by teachers. Not all teachers submit videos and the number of students assessed via these videos is not reported, only the total number of videos (20 from treated and 17 from control). |
| Free of other bias (Judgement) | Unclear | 5 | 5 |
| Free of other bias (Description) | No demographics or pre-test shown, only mentioned that schools were "found to be equivalent to one another on baseline reading achievement and on all but one demographic characteristic." (p. 8). Random assignment was completed one year before the intervention was implemented, implying that teachers as well as students have plenty of time to select or deselect the treatment schools. | Although teachers are randomly assigned it is unclear if the students used for analysis are completely randomly selected. According to Ansari and Pianta 2018, they are selected after randomisation of teachers. It is stated that four eligible students with parent consent from each class was randomly selected; however: 'During the fall assessment, if a selected child was absent that day, the next eligible child was assessed. For the spring assessment, if a selected child had left the classroom (e.g., moved away), he or she was replaced with the next eligible child if possible. Whenever a selected child refused the assessment, the data collector first attempted later in the day, and if the child continued to refuse the data collector returned on a subsequent day.' (p. 961). Thus, pre and post-test students may not be the same and how the 'next eligible child' is chosen is not explained. Further it is not explained what happened if the child also refused assessment 'later in the day'. Child demographics not shown by group, teacher demographic (by group) only shown for the analysis sample and stated in the text that at no time were there any differences. Child pre-tests shown in table 3 but not all pre-test children are identical to post-test children (the average number of children per class with demographics reported in table 2 is 4,3 for the phase 2 year). The present study used only data from the English-language assessments and therefore excluded the children for whom English-language assessments were inappropriate, even though assessments in Spanish were given to these children (numbers excluded not reported but 13% in total did not have English as home language, table 2) | Two sequential cohorts involving a total of 49 preschool teachers were analysed, numbers or results by year not reported. Data were collected for a random sample of students (n = 330) selected from each teacher’s classroom; approximately 40% of students (average class size is 17, only reported for year 2). Teachers reasonably balanced on gender, ethnicity, education and experience (table 1 and 2). No student characteristic or pre-test shown or mentioned by condition. How many students (and from how many teachers) used for the only outcome measures reported is not reported. Note that teachers assigned to the comparison condition received an equivalent amount of PD, albeit on topics unrelated to conversational responsiveness (the focus of the treatment PD). There is a clear risk that the comparison condition affected the child outcome measures, both behaviour management and storybook selection may have influenced the "linguistic productivity and complexity" measured by the video-based outcome measures. In addition, teacher outcomes could have been affected to different degrees. For example, self-efficacy seems to involve behaviour management questions. Some very large imbalances at week 2 for the child outcomes in Table 6, p. 396. Teachers film themselves and can choose which videos that are submitted to the study (p. 398). |
| A priori protocol (Judgement) | Unclear | Unclear | Unclear |
| A priori protocol (Description) |  |  | Not mentioned |
| A priori analysis plan (Judgement) | Unclear | Unclear | Unclear |
| A priori analysis plan (Description |  |  | Not mentioned |
| Confounding (Judgement) | Not relevant | Not relevant | Not relevant |
| Notes | Have contacted authors for clarifying high p-values |  | Same trial as reported in Cabell et al., 2011. Cabell et al., 2011 reports on student outcomes and Piasta et al., 2012 reports on students (but transcribed from video only, another outcome than reported in Cabell et al., 2011) |

| Study | Powell et al., 2010 | Rimm-Kaufman et al. (2014) | Sandilos et al. (2018) |
| --- | --- | --- | --- |
| Sequence generation (Judgement) | Unclear | Low | Unclear |
| Sequence generation (Description) | Teachers, stratified by geographical area, were randomised. Method not reported | 24 schools were assigned randomly to intervention or waitlist control condition using the random number function in Excel. | Teachers were assigned to treatment(s) randomly within site, method not reported. |
| Allocation concealment (Judgement) | Unclear | Low | Unclear |
| Allocation concealment (Description) |  | Non-sequential and therefore low risk of bias |  |
| Blinding (Judgement) | 4 | 3 | 4 |
| Blinding (Description) | Classroom observations and child assessments were conducted by members of the research team. Not reported that they were blinded | Assessors probably blinded 'A state data team summed the number of correct items, converted the value to a scaled score (ranging from 0 to 600), and transmitted data to the district. The research team garnered scores from the district.' (p. 582) | No information |
| Incomplete outcome data addressed (Judgement) | 2 | 1 | 5 |
| Incomplete outcome data addressed (Description) | Attrition rate teachers/students: 0%/3% for control and 2%/10% for treated (fall) | No attrition by construction: 'All second graders attending the 24 study schools in spring 2008 were enrolled in the study. Students entering third grade in 2008–2009, fourth grade in 2009–2010, and fifth grade in 2010–2011 at the 24 schools were included as participants. Two rationales guided student participant decisions: (1) Randomization occurred at the school level, and thus, our model included typical patterns of school transience (including exit and entrance) to improve ecological validity' (p. 576). Student attrition and entrance by condition reported in Figure 2. | 427 teachers randomised within 10 sites. By the end of intervention 239 had complete data (44% attrition/missing data rate), not reported by condition. P. 283: "The most common reason for missing data was that teachers dropped out of the study due to competing time commitments. In addition, several teachers were missing CLASS observational data because they took the course in the summer and they were not teaching at that time". Thus missing data must be imbalanced as only treated teachers can have missing data due to taking the course in the summer. Information from Hamre et al 2012: a number of teachers who signed up for the study never participated, numbers not reported (not even total number). Attrition and missing data rates by condition not reported in either Sandilos et al. 2018 or Hamre et al., 2012. According to Pianta et al 2017, the attrition rate is 22%, unevenly distributed with 13% for control and 31% for treated (figure 1). The remaining 22% with missing data may be as unevenly distributed |
| Free of selective reporting (Judgement) | 1 | 1 | Not relevant |
| Free of selective reporting (Description) |  |  |  |
| Free of other bias (Judgement) | 4 | 3 | Unclear |
| Free of other bias (Description) | Randomisation in several steps: First, teachers were randomly assigned to an intervention semester (fall or spring) and a participation year (first or second) within location (urban, not urban). Second, teachers within each intervention semester and location were randomly assigned to one of two interventions (analyses vs the control not separated). In the first year, one half of teachers assigned to the spring intervention semester (wait-list control) were randomly assigned to the control group in the fall semester and it is not explained why not all are used. In the second year, all teachers assigned to the spring intervention semester were used as control group (wait-list control) in the fall semester. There is a risk that control teachers may be contaminated by previous experience of the intervention, especially the second year control teachers are on wait-list for a year and a half. Results reported are not separated by year (all Fall treated and all serving as control for fall treated merged). Children aged 4 by December 31. The year their teacher participated and who had parent consent were eligible. Not reported if parent consent was sought before or after randomisation but for the second year, it had to be after. The assignment of teachers was known when children in the second year were assigned to classes. All teachers female, some imbalance on education and experience (table 1). Small imbalance on student gender and some imbalance on ethnicity (table 1). Pre-tests on both teachers and students reasonably balanced (table 2) | After random assignment, it became apparent from an informational survey that 2 schools in the control condition had received low-level exposure to the RC intervention. In each school, the principal reported that one of the third-, fourth-, or fifth-grade teachers (\15% of sampled teachers at each school) had received a 1-week RC training. For example, at one school, the principal and some teachers acquired two NEFC-published books and used them to self-teach RC practices. Because of the low-level contamination, one of the two schools was selected at random and placed into the intervention condition, resulting in 13 intervention and 11 control schools. (p. 576). Some imbalance on Free/reduced priced lunch (both student and school level), pre-test (schools level only) (table 1), and school level gender, otherwise reasonably balanced at both school and student level. Is the baseline measurement made after randomization (p. 576)? Unclear how "gathered" should be interpreted. Clearer on p. 579 that baseline occur after randomization. Not clear what students know though so perhaps not a problem. Possible selection of ELL students into a different version of the post-test (p. 580). Mention potential ceiling effects on the math test on p. 597. | Teacher demographics reasonably balanced but there is almost double as many Head Start affiliated classrooms in the treatment condition vs control; pre-tests reasonably balanced (table 2) but not reported how many teachers contributed to the pre-test. (In Hamre et al 2012 it is reported that around 75% of teachers had background data, not reported in any of the studies how many had pre-test) |
| A priori protocol (Judgement) | Unclear | Unclear | Not relevant |
| A priori protocol (Description) | Not mentioned |  |  |
| A priori analysis plan (Judgement) | Unclear | Unclear | Not relevant |
| A priori analysis plan (Description | Not mentioned |  |  |
| Confounding (Judgement) | Not relevant | Not relevant | Not relevant |
| Notes |  | Same trial as Ottmar 2013. Ottmar et al., 2013 reports only teacher outcomes for one of the years whereas Rimm-Kaufman et al., 2014 reports student outcomes for all years. | Same trial as Pianta et al., 2017 and Ansari & Pianta, 2018 but only phase 1 data and unlike the other two, they report teacher (CLASS) outcomes. Scores 5 |

| Study | Saraniero et al., 2014 | Scanlon et al., 2008 | Schwanenflugel et al., 2010 |
| --- | --- | --- | --- |
| Sequence generation (Judgement) | Unclear | Unclear | High |
| Sequence generation (Description) | Stratified on grade, teachers were randomly assigned via lottery, no other information concerning method is provided | Schools matched on SES, risk status for entering kindergarten students, and grade 4 achievements on the New York State English Language Arts assessment were randomly assigned to three conditions. Method not reported. | One school from a neighbour county chosen as control |
| Allocation concealment (Judgement) | Unclear | Unclear | High |
| Allocation concealment (Description) |  |  |  |
| Blinding (Judgement) | 4 | 4 | Not relevant |
| Blinding (Description) | State-administered normed test in English language arts is used but no information provided on blinding of assessors | No information concerning blinding is provided |  |
| Incomplete outcome data addressed (Judgement) | 3 | 4 | Not relevant |
| Incomplete outcome data addressed (Description) | A 3-year study. It is reported that up to 25 teachers were assigned to each of the two intervention groups each year and on average about 39 control teachers each year (p. 5). It is reported there were some attrition of teachers during the years and numbers used for analysis is 60/56/71 for intervention 1, 2 and control group. Attrition rates may be as high as 20%/25%/39%. Reasons stated for attrition is teachers being laid off or reassigned to other grades or schools. | 15 schools randomised, two excluded from the analysis as they shifted from half-day kindergarten during the Baseline Cohort's kindergarten year to full day kindergarten for the subsequent cohorts and one school was excluded because it was closed at the end of the Baseline Cohort's kindergarten year (overall school attrition 20%, not reported by condition). Teachers were reduced from 43 to 38 due to school exclusions. A further teacher attrition (of 10 teachers) occurred as a result of teachers retiring, taking leave, moving, or taking non-teaching positions (overall teacher attrition 35%, not reported by condition). |  |
| Free of selective reporting (Judgement) | 1 | 1 | Not relevant |
| Free of selective reporting (Description) |  |  |  |
| Free of other bias (Judgement) | 2 | 5 | 5 |
| Free of other bias (Description) | Characteristic of teachers randomised not shown, only for those analysed (table 1) and they are reasonably balanced. Student characteristic not considered, except analyses divided by grade and pre-tests are shown (table 10); only small pre-test imbalances. | Not reported if parent consent was sought before or after randomisation. Reported that in all but one school, over 90% of parents agreed to involve their children, not reported which school did have over 90% parent consent and as there are only four schools in each condition it may matter. Student ethnicity, gender and free lunch status in table 1, no large imbalances (only Implementation year is relevant). All teachers were women and Caucasian. There are very large differences between the 9 teachers in the 4 schools in the control condition (intervention only) and the 9 teachers in 4 schools in the Intervention + PD condition in the baseline year (no intervention or PD); both concerning reduction in number of at risk students from start to end of kindergarten (table 2) but also on the growth in student outcome measures (table 4 and 5). All randomised contrasts are comparisons between alternative treatments. | 16 schools in two counties randomised to four intervention condition. The control condition was from one large school from a third county. Cannot separate school/county effect from intervention effect |
| A priori protocol (Judgement) | Unclear | Unclear | Not relevant |
| A priori protocol (Description) | Not mentioned | Not mentioned |  |
| A priori analysis plan (Judgement) | Unclear | Unclear | Not relevant |
| A priori analysis plan (Description | Not mentioned | Not mentioned |  |
| Confounding (Judgement) | Not relevant | Not relevant | Not relevant |
| Notes |  | Only the Intervention only group compared to Intervention + PD group is relevant (the group PD only cannot be used in this review) |  |

| Study | Snow et al. (2014) | Wasik & Hindman, 2011 | Yoshikawa et al. (2015) |
| --- | --- | --- | --- |
| Sequence generation (Judgement) | Unclear | High | Unclear |
| Sequence generation (Description) | Schools randomised using stratification to ensure similar representation of schools across dioceses and rural/metropolitan regions. Method not reported | Three Head Start centres randomised, two to intervention and one to control | 64 schools randomly assigned to condition, method not reported other than it was 'a public lottery' |
| Allocation concealment (Judgement) | Unclear | High | Unclear |
| Allocation concealment (Description) |  |  |  |
| Blinding (Judgement) | 4 | Not relevant | 4 |
| Blinding (Description) | Personnel (SLPs and teachers) experienced in assessing early-years students completed all testing. Only mentioned that the T-unit analysis employed as a measure of expressive grammar (explained on p. 499 what that is) was undertaken by two SLPs who were blind to participant study arm. For the remaining tests, there is no mentioning of blinding. |  | Child outcome data used in the current report were collected by the evaluation team, No mentioning of blinding. Coders of classroom quality were blind to the intervention condition of the videos they coded |
| Incomplete outcome data addressed (Judgement) | 3 | Not relevant | 4 |
| Incomplete outcome data addressed (Description) | Follow-up assessments (RPT, standardized language measures and the narrative task) were performed on 503 (83.5%) students in Stream A, and 568 (87.1%) students in Stream B completed the RPT only, when students were in Grades 1 – 3. Retention was 87.6% in the research arm (across both Streams) and 81.8% across both Streams in the control arm. From table 2 (showing only the outcomes for the standardized language measures and the narrative task) it seems as the missing data rate for treated in stream A is 13% and for control 14%. Differential attrition significant (p. 501). |  | We had complete classroom data and teacher/child demographic data on 85 classrooms at the pre-test (94.4% completion rate): 76 classrooms at the end of prekindergarten (84.4% completion rate), and 72 classrooms at the post-test (80% completion rate). On average, we had complete child outcome data and teacher/child demographic data on 1,584 (language and literacy; 84% completion rate), and 1,204 (self-regulation and low problem behaviour; 64.1% completion rate) children at the pre-test, and 1,354 (language and literacy; 72.1% completion rate), and 1,078 (self-regulation and low problem behaviour; 57.5% completion rate) children at the post-test. Only small differences between conditions. Overall missing data level on children’s socioemotional skills too high to be used in analysis |
| Free of selective reporting (Judgement) | 4 | Not relevant | 4 |
| Free of selective reporting (Description) | The Reading Progress Test (RPT) results only showed for stream A and B together although excluding grade 2 students with no explanation of why. Only total number of treated/control in stream A and B and total number of KG, 1 and 2 grade students reported, not possible to calculate cell numbers |  | Raw means or SD not reported only adjusted means. Pre-tests and other covariates not shown. No reporting of demographic or pre-test differences, only significance results from t tests at school level (few observations and even large differences need not be significant). Results from their robustness check (using ordinary least squares regression with correction of the standard errors (Huber-White) for clustering) is not shown |
| Free of other bias (Judgement) | 5 | 5 | Unclear |
| Free of other bias (Description) | Large difference in mean Socio Economic Indexes for Areas (SEIFA) ranking for treated schools compared to control schools (1.5 SD). Randomly select students at the school for participation (grades KG, 1 and 2). Not reported if (written) parent consent was sought before or after randomisation and not reported how many denied participation, only that these students were not included in the analysis. The author’s state that the total sample of 1254 students was randomly allocated into two streams maintaining the proportional representation of treated and control schools. Baseline measurement possibly done after randomization as well (p. 498). Stream A completed baseline assessments of oral language and reading abilities and stream B underwent classroom-based reading assessment only. It seems however unlikely that the allocation was totally random; no 2 grade students were allocated to stream A, 70% of KG students and 75% of 1 grade students were allocated to stream A; of the total number of treated students 38% were allocated to stream A whereas 64% of the total number of control students were allocated to stream A (only margins are reported, i.e. total number of treated/control in stream A and B and total number of KG, 1 and 2 grade students in stream A and B, it is not possible to calculate the cell numbers from these margins). Numbers do not add up, the margins reported in figure 2 and the numbers reported in table 3 implies there are less students from control schools combining stream A and B than from stream A alone. Student demographics not reported separately for the two streams, overall economically disadvantaged, recipients of disability funding and minority (Aboriginal and Torres Strait Islander) was reasonably balanced whereas there was some imbalance on language background other than English. Imbalance on two of six pre-tests (oral language and narrative language stream A only), 0.3-0.38 SD. Imbalance on the RPT pre-test (stream A and B excluding grade 2 students, unclear why they are excluded), appr. 0.3 SD. No teacher information at all reported. | Three Head Start centres randomised, two to intervention and one to control. Cannot separate centre effect from intervention effect (p. 458). | Consent to participate probably obtained after randomisation, but the recruitment rate for teachers was 99.1% and 98% for students. 2.3% (24 of 1,033) of children assigned to the Full UBC condition moved to the Comparison condition, while 1.4% (12 of 843) of children assigned to the Comparison condition moved to the Full UBC condition. No reporting of demographic or pre-test differences (student nor classrooms), only significance results from t tests at school level (few observations and even large differences need not be significant). The CLASS ratings in this study are based on videos rather than observations (p. 315). (They state that no meaningful differences between live and video observations have been found). (https://curry.virginia.edu/classroom-assessment-scoring-system). However, since teachers can select the videos they send in, ought to be a higher risk of bias than live observations. Some indications that CLASS and vocabulary measures are insensitive to changes (p. 319). |
| A priori protocol (Judgement) | Unclear |  | Unclear |
| A priori protocol (Description) |  |  |  |
| A priori analysis plan (Judgement) | Unclear |  | Unclear |
| A priori analysis plan (Description |  |  |  |
| Confounding (Judgement) | Not relevant | Not relevant | Not relevant |

### Stress reduction

| Study | Flook et al. 2013 |
| --- | --- |
| Sequence generation (Judgement) | Unclear |
| Sequence generation (Description, quote from paper or describe key information) | 18 teachers randomised, method not reported. |
| Allocation concealment (Judgement) | Unclear |
| Allocation concealment (Description, quote from paper or describe key information) |  |
| Blinding (Judgement) | 4 |
| Blinding (Description, quote from paper or describe key information) |  |
| Incomplete outcome data addressed (Judgement) | 3 |
| Incomplete outcome data addressed (Description, quote from paper or describe key information) | No teacher attrition but the Teacher classroom behaviour coding (CLASS) was completed on only a sub-sample of 13 participants (72%), 7 treated (70%) and 6 control (75%), no explanation of why only a subset and how they were chosen |
| Free of selective reporting (Judgement) | 1 |
| Free of selective reporting (Description, quote from paper or describe key information) |  |
| Free of other bias (Judgement) | 5 |
| Free of other bias (Description, quote from paper or describe key information) | Teacher classroom behaviour coding (CLASS) was completed on a sub-sample of 13 participants, 7 treated and 6 control, no explanation of why only a subset and how they were chosen. No demographic by group reported, except age, which is, unbalanced (8-year average difference); otherwise, p-values are reported but with a limited number of participants even large differences may be non-significant. Pre-tests highly unbalanced (-0.87 SD, -0.24 SD, -0.89 SD). |
| A priori protocol (Judgement) | Unclear |
| A priori protocol (Description, quote from paper or describe key information) |  |
| A priori analysis plan (Judgement) | Unclear |
| A priori analysis plan (Description, quote from paper or describe key information) |  |
| Confounders | Not relevant |

## Appendix F: Numerical data

### Social and emotional development, studies used in meta-analysis

| Study | Fukkink & Tavecchio, 2010 | Hickey et al. (2017) | Jennings et al. (2017) |
| --- | --- | --- | --- |
| Type of outcome | Continuous | Continuous | Continuous |
| Professional or other? | Professional | Student | Teacher |
| Outcome (there may be more than one, record them all) | Caregiver interaction scale (Arnett, 1989), Author-constructed scales to capture sensitive responsivity and verbal simulation. VIG-specific measures. Job resource scale and VIG job satisfaction scale. Not relevant outcomes according to review protocol. | Socioemotional skills of the children (emotional symptoms, conduct problems, hyperactivity, peer relationship problems, prosocial behaviour) | Student–teacher interactions, CLASS 3 domains: (i) Emotional Support; (ii) Classroom Organization; (iii) Instructional Support, summaries and all subscales |
| Time Point (s) (record the exact time, there may be more than one, record them all) | End of intervention | End of intervention | End of intervention |
| Source (questionnaire, admin data, other(specify) or unclear) | Caregiver interaction scale (Arnett, 1989): stimulating and authoritarian caregiving behaviour - validated and delivered by trained assessors. Other scales were constructed so not used here. | Strengths and Difficulties Questionnaire (SDQ) - teacher completed | CLASS. The K–3 version of the CLASS was used for all classrooms (K–5) |
| Valid Ns (only applicable for continuous outcome data). Mention treatment and comparison. | 95 teachers in total. n =52 for the experimental group, n = 43 for the control group | Treated: 11 teachers, 110 students and 11 schools; Control: 11 teachers, 107 students and 11 schools | Cohort 1 consisted of 53 teachers from 8 schools, and Cohort 2 consisted of 171 teachers from an additional 28 schools. In total 118 teachers assigned to treatment and 106 assigned to the wait-list control condition. |
| Method of estimation | Pre-test and post-test means and SD. Effect sizes (using corrected means; Covariates included pre-test scores, along with the addition of age and work experience of the childcare teachers.). Also report raw means and SD | Robust multiple linear regression was used to examine post-intervention outcomes for index children and the entire classroom, controlling for treatment group, classroom and baseline score. Report raw means (SD) pre, post, and ICC for all outcomes. Report eta squared effect sizes with 95% CI (we do not use this) | Hierarchical linear regression controlling for pre-test scores, cohort, grade level, classroom type, student teacher ratio, teacher race, proportion of students with an IEP or 504 plan, proportion of students ever suspended, and teacher perceived average level of support for learning in the home. Means and SD (both pre and post) also reported |
| Statistics (risk ratio, odds ratio, standard error, 95 cf, DF, p-value, chi2) | ES | Mean difference from model and pre SD by condition and ICC | Mean difference from regression models reported and effect sizes dividing by unadjusted pooled standard deviations (p. 10). Unadjusted means and SD (both pre and post) |
| Page numbers and notes | Table 2. Only use results for "Stimulating caregiving" and "Authoritarian caregiving" since they are from the validated Arnett-scale. NB that a decrease in the latter is a favourable development. See note about corrected means used for ES estimation | Table 4 | Table 2 and table 5 |
| Level of aggregation | Teacher | Student and clustering taken into account | Teacher |
| Notes | ES as reported, number of teachers T/C: 52/43 | The teacher measure is not standardised | Use only CLASS outcome |
| Used in meta-analysis? | Could not be pooled with other studies but two effect sizes are shown in a forest plot by themselves. Centres are randomised, should correct for clustering, number of centres not reported | Yes, student total score. No need for cluster correction. Teacher outcome cannot be used | Three summary CLASS outcomes used, should correct for clustering. Did not use the reported ES. The adjusted ES are 0.22, 0.19 and 0; whereas the unadjusted are 0.15, 0.14 and -0.03 (all SE=0.13). Should correct for clustering |

| Study | Jensen et al. (2017) | Murray et al. (2014) | Raver et al. (2008) |
| --- | --- | --- | --- |
| Type of outcome | Continuous | Continuous | Continuous |
| Professional or other? | Other (children) | Professional and students | Professional |
| Outcome (there may be more than one, record them all) | Socioemotional skills of the children (emotional symptoms, conduct problems, hyperactivity, peer relationship problems, prosocial behaviour) | Observational change in teacher practices based on CLASS, of which subscales used for this study: Positive Climate, Negative Climate, and Behaviour Management. Student reading and math academic scores and teacher rated social competences. TCI seems to have at least two subscales: Harsh and Competent (see note in Table 1 of paper) | CLASS (classroom quality): 4 subscales: Positive climate, Negative climate, Teacher sensitivity, Behaviour management |
| Time Point (s) (record the exact time, there may be more than one, record them all) | p. 29: Pre (March 2011) and post (March 2013), also collected mid-way in March 2012 but not used in main estimations (rather to do robustness checks on results and check validity of teacher reported SDQ | End of intervention | End of intervention. Spring (March) |
| Source (questionnaire, admin data, other(specify) or unclear) | Strengths and Difficulties Questionnaire (SDQ) - teacher completed. Five subdomains, total SDQ score, SDQ impact score. All SDQ scores are normalised by pre-test SD. in control group | CLASS (Pianta & Hamre, 2005) and the Teacher Coder Impressions Inventory (TCI; Webster-Stratton, Reid, & Hammond, 2001). Revised Teacher Social Competence (R-TSC) scale (teacher-rated); Conners' DSM_IC Inattention scale (Conners, 2001); Academic competence subscale of R-TSC; Star Early Literacy/Reading and Math (STAR) (nationally-normed computerized adaptive test) | Observations using 7-point Likert scales |
| Valid Ns (only applicable for continuous outcome data). Mention treatment and comparison. | Total = 58 pre-schools; 29 control; 29 intervention; 686 children - 396 intervention and 290 control | 97 teachers, 47 allocated to intervention group of which 45 participated. Student data available for analysis: 598 intervention, 560 control | n=18 sites: 9 Intervention and 9 Control |
| Method of estimation | Regression model (pre-test not included - Table 5 (p. 33), and other factors, e.g. gender...) | Unadjusted (raw) means and SDs as well as predicted changes in outcomes taking account of nesting of students within teacher, grade level and school | HLM Difference in means after adjusting for baseline predictors |
| Statistics (risk ratio, odds ratio, standard error, 95 cf, DF, p-value, chi2) | ES and SE | Raw means and SD. Predicted changes, in which various background characteristics are taken account of. | Mean difference coefficient, SE and ES |
| Page numbers and notes | Table 5, p. 33. Total SDQ score. Sub-groups are available | Table 1, p. B-1; (Note that only TCI Competent has been reported. According to note at bottom of table, there is also a TCI Harsh component. Reason for non-reporting is not provided; we use only the CLASS measures for teachers). | Tables 1, 2 and 3 page 12 and pages 23-25 HLM to adjust for clustering |
| Level of aggregation | Child level, but adjusted for clustering | Teachers and students, since there are outcomes for both | Class and site level using HLM to account for clustering at two levels |
| Notes | Adjusted for clustering (p. 33) Table 5 Pre-schools randomised but analysis on child level | TCI not fully reported, we do not use it. |  |
| Used in meta-analysis? | ES and SE reported | Post means and pre SDs. Number of teachers T/C: 45/50. Number of students T/C: 598/560. Eleven schools. 36 KG teachers, 26 1^st^ grade and 35 2^nd^ grade. CLASS measures used (no need for cluster correction as within school randomisation). Student outcome used a simple average of math, reading and early literacy. Within school randomisation of grades (K-2). Should correct student measures for clustering. | ES reported. Number of teachers T/C: 41/41 (assuming an equal number in both groups: (87-4)/2 |

| Study | Reinke et al. (2016) | Rubie-Davies et al. (2015) | Snyder et al. (2018) |
| --- | --- | --- | --- |
| Type of outcome | Continuous | Continuous, test scores range from approx. 1100 -1900 | Continuous |
| Professional or other? | Student | Other (children) | Student |
| Outcome (there may be more than one, record them all) | Disruptive Behaviors, Concentration Problems, Emotional Dysregulation, and Prosocial Behavior subscales from the TOCA-C, the academic competence subscale and the overall social competence scale from the T-COMP (note academic competence is included in the overall social competence scale), standardised academic achievement in Broad Reading and Broad Math. | Mathematics and reading achievement | Reading, language, school readiness, social skills and problem behaviour |
| Time Point (s) (record the exact time, there may be more than one, record them all) | End of intervention | Beginning (mid-end February), middle (mid-end June) and end (end November) of school year. Academic year in NZ runs from beginning February to mid-December. | End of intervention |
| Source (questionnaire, admin data, other(specify) or unclear) | The Teacher Observation of Classroom Adaptation-Checklist (TOCA-C; Koth, Bradshaw, & Leaf, 2009), The Revised Social Competence Scale-Teacher version (T-COMP; Gifford-Smith, 2000), The Woodcock-Johnson III Normative Update Tests of Achievement (WJ III ACH; Woodcock, McGrew, & Mather, 2007) was conducted with each child. | Mathematics and reading achievement assessed using e-asTTIe, an online assessment tool. Curriculum strands selected by first author for this study. | The standardized child outcome measures administered to children were the Bracken Basic Concept Scale–Third Edition: Receptive (BBCS-3R; Bracken, 2006), the Preschool Language Scale–4 (PLS-4; Zimmerman, Steiner, & Pond, 2002), and the Test of Early Reading Ability–Third Edition (TERA-3; Reid, Hresko, & Hamill, 2001). Teachers completed the Preschool and Kindergarten Behavior Scales–2 (PKBS-2; Merrell, 2002) for each target child. |
| Valid Ns (only applicable for continuous outcome data). Mention treatment and comparison. | 9 schools (105 teachers and 1680 students for the analyses of social and behavioural outcomes; 105 teachers and 1685 students for the analyses of academic achievement outcomes). Treated: Teachers (n=53) Students (n=833) and control: Teachers (n=51) Students (n=847) | 84 teachers randomised 43 intervention; 41 control. Students: T/C: Math: 831/808; Reading: 852/707. | T1: 12 teachers 32 students, T2: 11 teachers 33 students, C: 12 teachers 34 students |
| Method of estimation | Hierarchical linear model, 3 levels | Test score means | Means and SD both pre and post. Adjusted means also available but includes interaction terms if the interaction is significant so cannot be used. Glass’s Δ and cluster corrected p-values only reported for outcomes that are significant and/or 'noteworthy (i.e. >0.2 in absolute terms) |
| Statistics (risk ratio, odds ratio, standard error, 95 cf, DF, p-value, chi2) | Coefficient (mean difference) from regression and pre-test SD. Social competence from T-COMP includes emotional regulation and prosocial behaviour. Therefore, emotional dysregulation and prosocial behaviour from the TOCA is not used (measuring the same). The remaining two subscales from TOCA shown separately. Should correct for clustering. | ES | Means and SD both pre and post |
| Page numbers and notes | Tables 2 and 3 | Table 2, p. 80: N, means and SD T1 (beginning), T2 (middle), T3 (end) of school year. | Table 3 |
| Level of aggregation | Student | Student level results | Student |
| Notes |  |  | None of the teacher measures is standardized. |
| Used in meta-analysis? | Average of student math and reading scores, report ICC, used. | Used means from T3 and total SD from T1. Number of students T/C: Math: 831/808; Reading: 852/707. Simple average of math and read ESs. Should correct for clustering | Average of reading and language (2 language subscales, auditory and expressive) outcomes, average of the two interventions. Should correct for clustering. School readiness, social skills and problem behaviour shown separately |

### Social and emotional development, studies not used in meta-analysis

| Study | Murray et al. (2018) | Reinke et al. (2018) | Seabra-Santos et al. (2018) |
| --- | --- | --- | --- |
| Type of outcome |  |  | Continuous |
| Professional or other? |  |  | Student |
| Outcome (there may be more than one, record them all) |  |  | Student Social Skills and Problem Behavior |
| Time Point (s) (record the exact time, there may be more than one, record them all) |  |  |  |
| Source (questionnaire, admin data, other(specify) or unclear) |  |  | Preschool and Kindergarten Behavior Scales—2nd edition (PKBS-2) |
| Valid Ns (only applicable for continuous outcome data). Mention treatment and comparison. |  |  | Treated: 31 teachers, 500 students; Control: 31 teachers, 469 students. Total 52 schools |
| Method of estimation |  |  | Perform linear mixed model regression with lunch status as covariate and use change scores as dependent variable. Report effect size (of the change scores) where the variance of the between-level data (at the classroom level) is 'portioned out' of the calculation (unclear what is meant by 'portioned out'). Impute missing data using the multiple imputation (MI) procedure. Raw means and SD not reported, only 'Estimated marginal means' and Means and SDs reported are from pooled results from m = 30 datasets (30 imputed datasets). Unclear if the SDs reported are with the between classroom SD 'portioned out'. |
| Statistics (risk ratio, odds ratio, standard error, 95 cf, DF, p-value, chi2) |  |  | Estimated marginal means' and SDs from pooled results from m = 30 datasets (30 imputed datasets) both pre and post. Unclear what is meant by 'estimated marginal means' and how the SDs are calculated. |
| Page numbers and notes |  |  | Table 2 |
| Level of aggregation |  |  | Student |
| Notes | Same trial and content as Murray 2014 | Same trial and same content (except descriptive table) as Reinke, 2016 | Mail sent to first author to clarify uncertainty concerning reported means and SDs February 13. 2019 |
| Used in meta-analysis? | Not used, Murray, 2014 used | Not used, Reinke, 2016 used | Not used as the uncertainty is not resolved |

### Language and literacy, studies used in meta-analysis

| Study | Al Otaiba et al., 2011 | Allen et al. (2011) | Buysse et al., 2010 |
| --- | --- | --- | --- |
| Type of outcome | Continuous | Continuous, test scores standardized on a 200-600 point scale. | Continuous |
| Professional or other? | Students | Other (children) | Professionals and students |
| Outcome (there may be more than one, record them all) | Picture Vocabulary (not shown). Letter Sound Fluency. Letter Word, Word Attack, Nonsense Word Fluency (NWF) and Phoneme Segmenting Fluency (PSF) | Student achievement in various courses: math/science, language arts/social studies, p. 2. Teachers: Observations based on videos, which are analysed according to CLASS-S, but this is part of the intervention and hence a mediating factor more than an outcome as such. | Quality of the classroom practices (*ELLCO* (classroom observation scale total, literacy activities rating scale total, literacy environment checklist total) and the ELLCO Addendum for English Language Learners |
| Time Point (s) (record the exact time, there may be more than one, record them all) | End of intervention | End of intervention year and end of post-intervention year (in course of teachers' choice according to study instructions) | End of intervention |
| Source (questionnaire, admin data, other(specify) or unclear) | Vocabulary: Picture Vocabulary subtest of the WJ-III (Woodcock) test (not shown?). Letter Sound Fluency using the AIMSWeb Letter Sound Fluency (Shinn & Shinn, 2004). Word reading skills: WJ-II Letter Word Identification (Woodcock) test. Ability to decode pseudo-words: Word Attack subtest of the WJ-III (Woodcock) test DIBELS Nonsense Word Fluency (NWF) and Phoneme Segmenting Fluency (PSF) tasks | Commonwealth of Virginia Standards of Learning (SOL) testing system. | The Early Language and Literacy Classroom Observation (ELLCO) Toolkit (Education Development Center, 2002) and the ELLCO Addendum for English Language Learners (Castro, 2005). Woodcock Language Proficiency Battery-Revised: English and Spanish Forms (WLPB-R; Woodcock, 1991;Woodcock & Munoz-Sandoval, 1995); the Peabody Picture Vocabulary Test (PPVT-III; Dunn & Dunn, 1997) and corresponding Test de Vocabulario en Imágenes Peabody (TVIP; Dunn, Padilla, Lugo, & Dunn, 1986); the Phonological Awareness Tasks (PAT; Miccio & Hammer, 2002); Naming Letters (National Center for Early Development & Learning, 2003); and Where’s My Teddy Story and Print Concepts (FACES: The Head Start Child and Family Experiences Survey, 2003) |
| Valid Ns (only applicable for continuous outcome data). Mention treatment and comparison. | Treatment: 305 students, 23 teachers, 7 schools; Control 251 students, 21 teachers, 7 schools | 78 teachers; Intervention year: 1267 students in 76 classrooms. Post-intervention year: 970 additional students in 61 classrooms (61 teachers). | Treatment: 92 students, 26 teachers; Control: 101 students, 29 teachers |
| Method of estimation | None (other than means) that can be used in the review | End-of-year student achievement test scores I vs C, but unclear where these are raw scores or whether they have been adjusted for achievement test scores from previous year, teacher, and student demographic characteristics, etc. See paper p. 3. and note at bottom of Table S1 p. 2 in SOM. | Means |
| Statistics (risk ratio, odds ratio, standard error, 95 cf, DF, p-value, chi2) | Means, SD (post and some pre) and number of students and teachers | Post-intervention year (2 year study period by design): End of Year Achievement Test Scores, MEAN (SD) | Means, SD (pre and post) and number of students and teachers all divided by grade |
| Page numbers and notes | Table 4 p. 552 | Supplementary Online Material Table S1. Achievement Test Scores I vs C in Intervention Year p. 1 and Post-Intervention Year p. 2. Means and SD. | Table 2 p. 200 and Table 4 p. 201 |
| Level of aggregation | Student | Student | Classroom and student |
| Notes | Schools are randomised and data is provided on student level | NB: Note at bottom of table states "Analyses used hierarchical linear models". Not clear whether results in S1 are raw or adjusted scores but assume the note is for Table S2 reporting regression results (which we cannot use as they are not in SD units) | An analysis of students adjusting for clustering is available but it analyses gain scores so means are used for meta-analysis |
| Used in meta-analysis? | Means and pre SD used. Average of five outcomes. Report ICC of 0.2 which we used to correct for clustering | Used post intervention year means and prior year SDs (in order to use SDs from the same students as post means). Number of students T/C: 419/551; number of classes 61 Teachers randomised. Should correct for clustering | Means and pre SD used. Average of eight outcomes given in Spanish and English (total sixteen). Should correct for clustering. Three teacher outcomes from two sources also available. Used an average of the three ELLCO outcomes (no need for cluster correction as classrooms randomised) |

| Study | Cabell et al., 2011 | Early et al. (2017) | Garet et al., 2008 |
| --- | --- | --- | --- |
| Type of outcome | Continuous | Continuous | Continuous |
| Professional or other? | Students | Professional | Students |
| Outcome (there may be more than one, record them all) | Children’s language skills (4 measures) and emergent literacy skills (3 measures) | Teacher - child interactions using validated CLASS, 3 domains: (i) Emotional Support; (ii) Classroom Organization; (iii) Instructional Support. Additional teacher outcomes (p. 61-62) | Reading |
| Time Point (s) (record the exact time, there may be more than one, record them all) | End of intervention | End of intervention | End of intervention and one year follow up |
| Source (questionnaire, admin data, other(specify) or unclear) | Children’s language skills: Grammar using “composite of two subtests from the standardized, norm- referenced CELF Preschool–2 (Wiig et al., 2004)”- The Word Structure subtest and the Sentence Structure Subtest. Receptive vocabulary – Peabody Picture Vocabulary Test-III (Dunn and Dunn, 1997). Expressive Vocabulary subtest of the CELF Preschool-2. Emergent literacy skills: Print concept knowledge - 14-item Preschool Print and Word Awareness test (Justice, Bowles, & Skibbe, 2006). Upper-Case Alphabet Knowledge and the Lower-Case Alphabet Knowledge tasks of the Phonological Awareness Literacy Screening for Preschool (Invernizzi, Sullivan, Meier, & Swank, 2004) | Classroom Assessment Scoring System (CLASS; Pianta et al. 2008) | Reading test scores, collected from district records. |
| Valid Ns (only applicable for continuous outcome data). Mention treatment and comparison. | Treatment: 130 students, 19 teachers; Control: 121 students, 19 teachers. State that 38 centres were randomised. | Final sample (p.60): Total (over the three years): 486 teachers in 336 schools/centre at pre-test and who had pre- and post-test CLASS observations. MMCI: 175; MTP: 151; Control: 160. | Total: 5500 students, 270 teachers, 89 schools |
| Method of estimation | Means | Pre-test and post-test means, SD., range. HLM model | A three-level hierarchical model with students nested within teachers and teachers nested within schools is applied. Regression-adjusted mean outcome levels for treatment group A, treatment group B, and the control group are reported, the adjustments were made using the observed mean covariate values for the control group. Non-adjusted standardized means are also reported |
| Statistics (risk ratio, odds ratio, standard error, 95 cf, DF, p-value, chi2) | Means, SD (pre and post) and number of students and centres. ICC for each measure reported (and used) | Raw means and SD. Effect sizes while controlling for pre-test scores (HLM regression). (Predicted odds of reaching predefined cut points at post-test also available) | Effect size and SE. Only effect sizes based on standardized test scores reported (as different tests are used by districts). Student test scores were standardized by using the overall mean and standard deviation within each district for the 2004–2005 baseline cohort, including only the schools participating in the study |
| Page numbers and notes | Table 3 and 4 p. 323 and 324 | Table 2, p. 62, raw means. Table 3, p. 65 has results (ES) which take account of pre-test scores and clustering. Table 4, p. 66 has predicted odds of reaching cut-points at post-test. | Table 4-3 and L-3 (without covariates) |
| Level of aggregation | Student but ICC for each outcome reported | Teacher | Student |
| Notes | An analysis of students adjusting for clustering is available but it analyses gain scores so means and the reported ICC are used for meta-analysis. At the centre level, ICCs ranged from .003 on receptive vocabulary to .25 on alphabet knowledge.  Same trial as in Piasta et al., 2012. Piasta et al., 2012 reports on students (but transcribed from video only, another outcome than reported in Cabell et al., 2011) | Cluster RCT: schools/centres in Cohort 1 (first year) blocked by region and randomly allocated to one of 3 arms. In years two and three (cohorts 2 and 3) classrooms (not teachers) were randomly allocated (no blocking), i.e. allowing different classrooms within same school/centre to have same prob. for participation in each of the 3 conditions. Report ICC: Intraclass correlation coefficients (ICC), a measure of the ratio of the variance that lies between school/centre to the total variance, were 0.19 for Emotional Support, 0.21 for Classroom Organization, and 0.35 for Instructional Support. | As the adjustments were made using the observed mean covariate values for the control group, I think we should use the non-adjusted ESs. Note that no cluster adjustment is necessary |
| Used in meta-analysis? | Means and pre SD used. Average of seven measures. Correct for clustering using the reported ICCs for each outcome. | Report ESs. Number of teachers used: MMCI: 175; MTP: 151; Control: 160. Used only the 3 CLASS outcomes. Report ICC but clustering is taken into account. | Non-adjusted ES used. Average of the two interventions. No adjustment for clustering is necessary. Only used end of intervention time point. |

| Study | Jayanthi et al. (2018) | Johnson et al. (2017) | Kammermeyer et al., 2016 |
| --- | --- | --- | --- |
| Type of outcome | Continuous | Continuous | Continuous |
| Professional or other? | Student | Teacher | Students |
| Outcome (there may be more than one, record them all) | Student reading and Oral vocabulary | Student–teacher interactions, CLASS 3 domains: (i) Emotional Support; (ii) Classroom Organization; (iii) Instructional Support, summaries and all subscales | Phonological awareness and Numerical competencies |
| Time Point (s) (record the exact time, there may be more than one, record them all) | End of intervention | End of intervention | End of intervention |
| Source (questionnaire, admin data, other(specify) or unclear) | Woodcock Johnson III (WJ) Reading Vocabulary and Oral Vocabulary subtests (Woodcock, McGrew, & Mather, 2001), Group Reading Assessment, and Diagnostic Evaluation (GRADE) Word Meaning subtest (Williams, 2001). | Pre-K Classroom Assessment Scoring System (CLASS; Pianta et al. 2008a). | To measure literacy and numeracy achievement wortgewandt & zahlenstark (‘eloquent & strong mathematical’) von Moser and Berweger (2007) was used |
| Valid Ns (only applicable for continuous outcome data). Mention treatment and comparison. | 16 districts, 4 states, 30 treated, and 31 control schools. Treated students 863 and control 817 | Two treated schools with a total of 12 teachers, two control schools with a total of 12 teachers | Treatment 1: 87 students, 26 teachers; Treatment 2: 107 students, 26 teachers; Control: 67 students, 28 teachers |
| Method of estimation | Multilevel regression with outcomes standardized to have a mean of zero and standard deviation of one. Schools were at Level 2. Student pre-test, gender, ethnicity and eligibility for free/reduced-price lunch, were used as student-level covariates. At the school level, rates of limited English proficient, minority students, free and reduced-price lunch, and economically disadvantaged students were used. | Means and pre SD | Means |
| Statistics (risk ratio, odds ratio, standard error, 95 cf, DF, p-value, chi2) | Hedge's g and p-value | Means and pre SD | Means, SD (pre and post) |
| Page numbers and notes | Table 7 | Table 1 | Table 4 p. 165 |
| Level of aggregation | Student | Teacher | Student |
| Notes | Two teacher measures reported but they altered the teacher measures, which means they invalidated any standardisation. Student scores are standardized at student level according to reply on mail from Prof. Jayanthi 21.03.2019 | Should correct for clustering | Number of students and teachers reported but not number of classes |
| Used in meta-analysis? | Yes, student average of the 3 measures. No need for cluster correction. Two teacher outcomes cannot be used Used as uncertainty concerning level of standardisation of SDs has been resolved | Used in meta-analysis (Teacher). Three summary CLASS measures | Means and pre SD used. Average of the two interventions. Should correct for clustering. A Numerical competencies measure also available (not used) |

| Study | Neuman & Cunningham, 2009 | Olson et al. (2017) Year one | Ottmar et al. (2013) |
| --- | --- | --- | --- |
| Type of outcome | Continuous | Dichotomous | Continuous. |
| Professional or other? | Professionals | Students | Professional |
| Outcome (there may be more than one, record them all) | The quality of language and literacy practices | Reading test, pass rates | Use of standards-based practices |
| Time Point (s) (record the exact time, there may be more than one, record them all) | End of intervention | End of intervention | 2008 –2009 school year, Three 3-month windows of observation were established: fall (September to November), winter (December to February), and spring (March to May)." The reported outcome is an average of these time points were the teachers were observed once for math and morning instruction during each observation window. |
| Source (questionnaire, admin data, other(specify) or unclear) | ELLCO for centre-based providers and the Child/Home Early Language and Literacy Observation (CHELLO; Neuman, Dwyer, & Koh, 2007) for home-based providers | California High School Exit Exam (CAHSEE) Pass Rates | Mathematics Scan (M-Scan; Berry et al., 2010); eight dimensions measured on a 7-point scale |
| Valid Ns (only applicable for continuous outcome data). Mention treatment and comparison. | Treatment 1: 80 teachers; Treatment 2: 85 teachers; Control 126 teachers | 16 schools (T and C in all schools). Year one: Control: 46 teachers, 1,493 students. Treatment: 49 teachers, 1,705 students. Year two: Control: 40 teachers, 939 students. Treatment: 41 teachers, 887 students. Only the outcome California High School Exit Exam (CAHSEE) is relevant for this review and only reported for 10th grade: year one: 262 control students and 313 treated; year two: 114 control students and 122 treated; number of 10th grade teachers not reported | 24 schools in total (13 intervention, 11 control schools); 94 teachers out of a possible 100 in the participating schools |
| Method of estimation | Means | Logistic regression | p. 445 "Model 2 tested the impact of the RC approach on the use of standards-based practices and included teacher-level variables (MKT, PMTE, MTOE, teacher experience, and average classroom achievement) and school-level variables (school assignment [RC vs. control], AYP status, and Title 1 status). Model 3 included the FOI variable. All level 1 variables were grand-mean cantered; while all three level 2 (school) predictors were uncentered." |
| Statistics (risk ratio, odds ratio, standard error, 95 cf, DF, p-value, chi2) | Means, SD (pre and post) | Number pass and fail and OR and T statistics | Mean difference coefficient and SD |
| Page numbers and notes | Table 5 p. 555 | Table 7 and 8 (number pass and fail) and table 9 (OR and T statistics) | Table 2, p. 447 "Table 2. Results from Two-Level HLM Model Examining the Impact of the RC Approach and the Contribution of MKT, Teacher Self-Efficacy, FOI, and School Contextual Factors on Mathematics Teaching Practices". Table 1 SD |
| Level of aggregation | Teacher (provider) | Student | The mean difference coefficient is on school level and the SD is probably on teacher level |
| Notes | Use the overall score only | Also included in school review (report 2015 version), do what we did there. Standard errors have been adjusted for clustering using the Huber-White “sandwich” estimator in STATA. Use the estimate with covariates but no interaction terms. Calculated SE from t value and transformed to SMD using the Cox transformation. Use only year one as risk of bias is lowest in year one. | Same trial as Rimm-Kaufman 2014. Rimm-Kaufman et al., 2014 reports student outcomes for all years. |
| Used in meta-analysis? | Means and pre SD used for the overall score. Average of the two interventions. No need for cluster correction | Used in meta-analysis (student). Only year one (least RoB) | Mean difference coefficient from model 2 (school level coefficient) and SD from table 1 (probably on teacher level). Number of teachers T/C 43/45 and schools 13/11. Report ICC of 0.13 which we use to correct SE. |

| Study | Parkinson et al. (2015) | Powell et al., 2010 | Rimm-Kaufman et al. (2014) |
| --- | --- | --- | --- |
| Type of outcome | Continuous | Continuous | Continuous |
| Professional or other? | Teachers and students | Professional and students | Students |
| Outcome (there may be more than one, record them all) | The quality of teachers’ classroom environment and literacy instruction (two subscales reported). Students' reading achievement. | The General Classroom Environment and the Language, Literacy, and Curriculum subscales of the ELLCO (Smith, Dickinson, Sangeorge, & Anasatosopoulos, 2002). Children’s receptive vocabulary skills, Letter Word Identification, understanding of print concepts, alphabet knowledge, blending and Initial sound matching. | Reading and mathematics |
| Time Point (s) (record the exact time, there may be more than one, record them all) | End of intervention | End of intervention | End of intervention (a 3 year intervention during 3rd, 4th and 5th grade) |
| Source (questionnaire, admin data, other(specify) or unclear) | Early Language and Literacy Classroom Observation (ELLCO) K–3 Research Tool and the Predictive Assessment of Reading (PAR) for kindergarten and the Group Reading Assessment and Diagnostic Evaluation (GRADE) for grade 1 and 2. | ELLCO (Smith, Dickinson, Sangeorge, & Anasatosopoulos, 2002). Peabody Picture Vocabulary Test—Third Edition (PPVTIII). Woodcock-Johnson III Tests of Achievement. The Concepts About Print (Clay, 1985). The Letter Naming assessment employed in Head Start’s Family and Child Experiences Survey (FACES; Zill & Resnick, 2006). A prepublication version of the Test of Preschool Early Literacy (Lonigan, Wagner, & Torgesen, 2007). The Alliteration Individual Growth and Development Indicator from Get it Got it Go! (Center for Early Education and Development, University of Minnesota, 2005). | The fifth-grade state standardized test, the Standards of Learning (Virginia Department of Education [VDOE], 2010). |
| Valid Ns (only applicable for continuous outcome data). Mention treatment and comparison. | 39 treated schools and 39 control schools. 4 districts and 3 states. Number of classrooms and teachers not reported. Number of students: Kindergarten cohort 1 sample size = 39 CLI schools and 38 control schools; 2,275 CLI students and 2,058 control students. Kindergarten cohort 2 sample size = 38 CLI schools and 37 control schools; 2,305 CLI students and 2,012 control students. Grade 1 cohort 1 sample size = 39 CLI schools and 38 control schools; 2,095 CLI students and 1,813 control students. Grade 1 cohort 2 sample size = 38 CLI schools and 37 control schools; 2,189 CLI students and 1,884 control students. Grade 2 cohort 1 sample size = 39 CLI schools and 38 control schools; 1,945 CLI students and 1,812 control students | Treatment: 310 students, 42 teachers; Control: 258 students, 31 teachers | Teacher participants (n = 276) were third-, fourth-, and fifth-grade teachers during the years of 2008–2009, 2009–2010, and 2010–2011 (95 third, 92 fourth, and 89 fifth). All second graders attending the 24 study schools in spring 2008 were enrolled in the study. Students entering third grade in 2008–2009, fourth grade in 2009–2010, and fifth grade in 2010–2011 at the 24 schools were included as participants. Final sample of students n = 2,904, 1467 in treatment schools and 1437 in control schools. |
| Method of estimation | Two-level hierarchical model with schools as second level and including students baseline achievement and percent ELLs in the school estimated within each of the four districts and weighting each districts estimate in proportion to the number of control schools in the study sample. Further: "Randomization block fixed effects were also included in the model. In cases with missing covariate measures, the missing data were replaced with district means, and a dichotomous variable indicating the missing status of a given covariate for each observation was added to the impact analysis model." (footnote 11). Regarding teachers: "The Impact model included randomization blocks as fixed effects and school-average percentage of students identified as ELL. Impact estimates were calculated for each district within a single model, and the overall difference between the CLI and control group was computed as a precision-weighted average across the four districts." (footnote 7) | Means | Structural equation modelling (SEM) results and raw means reported |
| Statistics (risk ratio, odds ratio, standard error, 95 cf, DF, p-value, chi2) | ES (using control group SD) and p-value | Means, SD (pre and post) | Standardised coefficients from SEL and raw means and SD as well as ICC |
| Page numbers and notes | Figures 3 and 4 and text | Table 2 p. 307 | Table 1 report raw means and SD and ICC reported on p. 585 |
| Level of aggregation | Teacher and student | Teacher and students | Student |
| Notes | Clustering is taken into account by the hierarchical models but unclear how p-values are calculated (they are pretty high even considering that clustering is taken into account) |  | P. 585: Intraclass correlation (ICC) values were computed to determine the percentage of school-level versus child-level variance for fifth-grade math and reading achievement. ICC values were 0.04 and 0.14, indicating that 4% of the total math variance and 14% of the total reading variance could be attributed to the school level. Same trial as Ottmar 2013. Ottmar et al., 2013 reports only teacher outcomes for one of the years |
| Used in meta-analysis? | Clarifying question concerning p-values and numbers of teachers send to Terry Salinger 14.1.2019. Average of the two teacher outcomes used. Average of the five student outcomes used. Reported effect sizes and p-values used | Means and pre SD used. Average of the seven student measures. Should correct for clustering (teachers randomised). Used average of the two ELLCO subscales. | Included in meta-analysis (student). Average ES of math and reading. Used reported ICC to correct for clustering (at school level) |

| Study | Saraniero et al., 2014 | Yoshikawa et al. (2015) |
| --- | --- | --- |
| Type of outcome | Continuous | Continuous |
| Professional or other? | Students | Students and teachers |
| Outcome (there may be more than one, record them all) | English language arts | Classroom quality (only Emotional support, Instructional support and Classroom organization), receptive language skills of students and early literacy skills of students (Vocabulary, Letter-word identification and Early writing). Children’s socioemotional skills (has too high attrition rate to be used) |
| Time Point (s) (record the exact time, there may be more than one, record them all) | End of intervention | End of intervention |
| Source (questionnaire, admin data, other(specify) or unclear) | State-administered normed test | CLASS; Pianta, La Paro, & Hamre, 2008. Subtests of the Woodcock-Muñoz Language Survey, Revised Spanish Form (WMLS-R; Woodcock, Muñoz-Sandoval, Ruef, & Alvarado, 2005). The Picture Vocabulary subtest was used to examine receptive language skills, and the Letter-Word Identification and Dictation subtests were used to examine early literacy skills. The Early Development Instrument (EDI; Janus & Offord, 2007), the Teacher Observation of Child Adaptation (TOCA–R; Werthamer-Larsson, Kellam, & Wheeler, 1991), and the Social Competence Scale, Teacher Version (Conduct Problems Prevention Research Group [CPPRG], 1990) and adapted for Chile. |
| Valid Ns (only applicable for continuous outcome data). Mention treatment and comparison. | Treatment 1: 677 students, 40 teachers; Treatment 2: 622 students, 34 teachers; Control: 792 students, 46 teachers | 32 schools, 51 classrooms, 66 teachers, 54 aides, and 1,033 children in the Full UBC condition and 32 schools, 39 classrooms, 53 teachers, 40 aides and 843 children in the Comparison condition (before attrition and missing data). After attrition and missing data: 39 classrooms and 833 students in treatment and 33 classrooms and 715 students in control, teacher attrition not reported |
| Method of estimation | Means | Multilevel linear regression models that accounted for the nesting of students within classrooms (for the child-level analysis) and classrooms within schools (for both the individual- and the classroom-level analysis. However: Note that not all child-level analyses required a classroom-level random intercept (e.g., when the intracluster correlation at the classroom was zero) and accordingly, a classroom-level random intercept was left out of models for those outcomes (specifically, the language and literacy outcomes). The investigators calculated effect sizes in both child- and classroom-level analyses by dividing the parameter by the standard deviation of the control group on the relevant outcome measure at post-test (not reported if this standard deviation was corrected for clustering but most likely, it was not). |
| Statistics (risk ratio, odds ratio, standard error, 95 cf, DF, p-value, chi2) | Means, SD (pre and post) and number of students and teachers all divided by grade | Adjusted means and effect sizes (no SD reported) |
| Page numbers and notes | Table 10 p. 18 | Table 1 and 2 |
| Level of aggregation | Student | Students and classroom |
| Notes | Teachers are randomised and data is provided on student level | Note they report no need for classroom correction on student outcomes so only need correction for school level (both student and classroom outcomes). Use only post-test (after kindergarten) as it was a two-year intervention and the measures taken after pre-kindergarten thus are intermediate effects. Used reported effect sizes. |
| Used in meta-analysis? | Means and pre SD used. Average of 3rd and 4th grade results of the two interventions used. Should correct for clustering | Student: average of three measures: Vocabulary, Letter-word identification, Early writing. Teachers: CLASS Emotional support, Instructional support and Classroom organization |

### Language and literacy, studies not used in meta-analysis

| Study | Allen et al. (2015) | Ansari et al. (2018) | Babinski et al. (2018) |
| --- | --- | --- | --- |
| Type of outcome | Continuous |  | Continuous |
| Professional or other? | Students |  | Student |
| Outcome (there may be more than one, record them all) | Course content specific: language arts/social studies/history vs. math/science |  | Picture Vocabulary, Verbal Analogies, Letter-Word Identification, Dictation, Understanding Directions, Story Recall, Passage Comprehension. |
| Time Point (s) (record the exact time, there may be more than one, record them all) | End of intervention |  | End of intervention |
| Source (questionnaire, admin data, other(specify) or unclear) | The Commonwealth of Virginia Standards of Learning (SOL) testing system (Commonwealth of Virginia, 2005; Hambleton et al., 2000). |  | Woodcock Munoz Language Survey-Revised |
| Valid Ns (only applicable for continuous outcome data). Mention treatment and comparison. | 5 schools, 44/659 treated teachers/students analysed and 41/536 control |  | Treated: 6 schools, 14 teachers, 64 students; control: 6 schools, 16 teachers, 41 students |
| Method of estimation | Hierarchical with student as level 1 and grade as level 2, note there are two grade levels (high school vs. middle school). Effect on student achievement obtained in level 2. Hedges g for simple comparison of means also reported. |  | Multilevel linear regression models that accounted for the nesting of students within teachers |
| Statistics (risk ratio, odds ratio, standard error, 95 cf, DF, p-value, chi2) | Regression coefficient (mean difference) from hierarchical model obtained from level 2 (grade). Hedges g mentioned in text, unclear at what level SDs for this is obtained. Something is wrong: They state in text: 'Hedge’s g for the simple comparison of outcome SOL scores between the MTP-S and the control group was .31. After inclusion of the covariates shown in Table 2, Hedge’s g was .48.' but the coefficient in table 2 (adjusted for covariates) gives a mean difference of 14.67 and using the SD in table 1 (although from prior year and not exactly same content) gives a Hedges g of 0.31. So how can it be that the simple comparison reported is 0.31 and it increases to 0.48 after adjustment |  | Adjusted means, SD (unclear if they are raw or 'adjusted'). |
| Page numbers and notes | Table 2 |  | Table 5, p. 134 |
| Level of aggregation | Grade level |  | Student |
| Notes | Cannot identify effect of PD on students as they only report second year outcomes and students in second year treatment (control) is an unknown mix of students who may have been treated for 1 and 2 years (unknown mix of students who may have been treated for 0 and 1 year) | Use same data as Pianta et al 2017 (scores 5) | The teacher measures are aggregated across the three observations to construct the outcome variables reported, cannot be used. Unclear if we can use student outcomes for this study, unclear if the standard deviation is the unadjusted, Table 5, p. 134. Mail has been send to authors 6 December 2018. In addition, they estimated 'a path-analysis analogue of a multivariate multiple linear regression model'. A bit unclear what that is and if they have any intermediate variables included. Note that the reported Hedge's g are: an estimated group difference in *change* in W scores. Later, when explaining models of subgroups they state: 'We conducted separate models for each of the seven subtests because the grouping was specific to a given subtest.' Therefore, it could be expected that the model of the seven subtest is actually a multivariate model. |
| Used in meta-analysis? | Not used in meta-analysis | Not used in meta-analysis | Not used in meta-analysis as uncertainty has not been resolved |

| Study | Bos et al., 1999 | Brownell et al. (2017) | Dickinson & Caswell, 2007 |
| --- | --- | --- | --- |
| Type of outcome | Continuous | Continuous | Continuous |
| Professional or other? | Students | Students | Professionals |
| Outcome (there may be more than one, record them all) | Spelling and dictation (all grades, K-2), sound identification (K only) and reading fluency (2nd grade only) | Student reading | Classroom Observation Scale, Literacy Environment Checklist, Literacy Activities Rating Scale and Learning Environment subscale |
| Time Point (s) (record the exact time, there may be more than one, record them all) | End of intervention | End of intervention | End of intervention |
| Source (questionnaire, admin data, other(specify) or unclear) | Three measures from the Woodcock-Johnson Tests of Achievement III | None of the teacher measures can be used are either modified or not standardized. The student tests included two subsections (Word Identification and Word Attack) of the Woodcock Reading Mastery Test–Revised (Woodcock, 1998) and the Dynamic Indicators of Basic Early Literacy Skills (DIBELS; Good & Kaminiski, 2002) assessment of fluency with connected text and word reading (The Oral Reading Fluency (ORF) test and The Nonsense Word Fluency subtest) | Three components of the Early Language and Literacy Observation (ELLCO) Toolkit (Smith et al., 2002) and two subscales from the Assessment Profile (Abbott-Shim & Sibley, 1998), Learning Environment and Interacting, both were used but only Learning Environment is shown |
| Valid Ns (only applicable for continuous outcome data). Mention treatment and comparison. | Treatment: 116 students; Control 205 students | 22/94 treated teachers/students and 20/76 comparison. 29 schools in total, not reported by condition but probably half in each condition | For the Classroom Observation Scale, the Literacy Environment Checklist, and the Learning Environment subscale, n = 62. For the Literacy Activities Rating Scale, n = 67. |
| Method of estimation | Means | Means and adjusted means and SD reported | Regression |
| Statistics (risk ratio, odds ratio, standard error, 95 cf, DF, p-value, chi2) | Means, SD (pre and post) and number of students all divided by grade | Means and adjusted means and SD reported. ICC reported for student measures | Coefficients and SE. Means and SD also provided |
| Page numbers and notes | Table 3 p. 235 | Text p. 156 and table 1 | Table 2 p. 250 |
| Level of aggregation | Student | Student | Teacher |
| Notes | An informal letter sound test also available. Scores 5 in confounding | Scores 5 on Other bias | Unclear if interaction terms are included, if they are the results cannot be used in the meta-analysis. Scores 5 on confounding |
| Used in meta-analysis? | Not used in meta-analysis | Not used in meta-analysis | Not used in meta-analysis |

| Study | Gallagher et al., 2011 | Gersten et al. (2010) | Hamre et al. (2012) |
| --- | --- | --- | --- |
| Type of outcome | Continuous | Continuous |  |
| Professional or other? | Students | Students (none of the teacher outcomes can be used in this review) |  |
| Outcome (there may be more than one, record them all) | 13 Family and Child Experience Survey (FACES) outcomes, 4 Preschool Learning Behaviors Scale outcomes and 6 Language and Emergent Literacy Assessment outcomes | Reading: Oral Vocabulary, Reading Vocabulary, and Passage Comprehension |  |
| Time Point (s) (record the exact time, there may be more than one, record them all) | End of intervention | End of intervention |  |
| Source (questionnaire, admin data, other(specify) or unclear) | Collectively termed as FACES outcomes: Peabody Picture Vocabulary Test (3rd ed), Woodcock-Johnson Psychoeducational Battery, Leiter-R Attention Sustain Scale, McCarthy Dra a design scale, Story and Print concepts. In addition to this: The Preschool Learning Behaviours Scale (PLBS), The Language and Emergent Literacy Assessment (LELA) | Teachers: Reading Comprehension and Vocabulary (RCV) Observation Measure (not a standardized measure), Relevant items from Content Knowledge for Teaching Reading (not relevant for this review). Students: three Dynamic Indicators of Basic Early Literacy Skills (DIBELS) measures (Good & Kaminski, 2002; Kaminski & Good, 1996) – Letter Naming Fluency, Phonemic Segmentation Fluency, and Oral Reading Fluency, and two sub-tests of the Woodcock Diagnostic Reading Battery (WDRB) – Word Attack, Letter-Word Identification for use as covariates and for exploratory analyses. Three other sub-tests of the WDRB – Oral Vocabulary, Reading Vocabulary, and Passage Comprehension were the main outcome measures. |  |
| Valid Ns (only applicable for continuous outcome data). Mention treatment and comparison. | It varies by outcome (see RoB the Incomplete outcome data item) |  |  |
| Method of estimation | Means | A two-level hierarchical model with students/teachers nested within schools (disregard classes) and intervention effect at school level |  |
| Statistics (risk ratio, odds ratio, standard error, 95 cf, DF, p-value, chi2) | Means, SD (pre and post) | Regression coefficients using measures standardized to have a mean of zero and standard deviation of one. Report only effects at the school level. |  |
| Page numbers and notes | Tables 5-7 | Table 7 p. 68 |  |
| Level of aggregation | Student | School |  |
| Notes | Score 5 on the Incomplete outcome data item | Cannot be used in meta-analysis as no results on student level. None of the teacher outcomes can be used in this review. Mail to request raw means and SD on student level sent 12.12.2018 to Dimino and Gersten | Same data as Sandilos, scores 5 on Incomplete outcome data |
| Used in meta-analysis? | Not used in meta-analysis | Not used as usable data not received | Not used in meta-analysis |

| Study | Hindman & Wasik, 2012 | Howlin et al., 2007 | Landry et al., 2009 |
| --- | --- | --- | --- |
| Type of outcome | Continuous | Raw scores were recoded into ordinal categories (0; 1 to 20; >20) and results presented as Odds Ratios (from ordinal regression) | Continuous |
| Professional or other? | Student and teachers | Students | Students |
| Outcome (there may be more than one, record them all) | Child vocabulary, alphabet knowledge and sound awareness. Classroom quality. | Expressive and receptive language | Vocabulary, composite language, phonological awareness, letter knowledge, and print awareness |
| Time Point (s) (record the exact time, there may be more than one, record them all) | End of intervention | End of intervention | End of intervention |
| Source (questionnaire, admin data, other(specify) or unclear) | The Peabody Picture Vocabulary Test—III (PPVT), the Uppercase Alphabet subtest of the Phonological Awareness Literacy Screening (PALS—PreK) and the Rhyme and Beginning Sound Awareness subtests of the PALS—PreK. ELLCO and CLASS. | The Expressive One Word Picture Vocabulary Test (EOWPVT: Academic Therapy Publications, 2000) and the British Picture Vocabulary Scales (BPVS: Dunn, Dunn, Whetton, & Burley, 1997) | The Expressive One-Word Picture Vocabulary Test (EOWPVT; Brownell, 2000), The Auditory Comprehension subtests of the English and Spanish versions of the Preschool Language Scale—fourth edition (PLS–4; Zimmerman, Steiner, & Pond, 2002), English and Spanish versions of the Developing Skills Checklist (DSC and La Lista, respectively), The Preschool Comprehensive Test of Phonological Processing (PCTOPPP; Lonigan, Wagner, Torgesen, & Rashotte, 2003) |
| Valid Ns (only applicable for continuous outcome data). Mention treatment and comparison. | Treated: 16 teachers and 626 students; Control: 10 teachers and 357 students | Total 84 | Total number of students varied between 1,607 and 1,678. Not reported by group |
| Method of estimation | Means | Multilevel ordinal regression models | ANCOVA |
| Statistics (risk ratio, odds ratio, standard error, 95 cf, DF, p-value, chi2) | Means, SD (pre and post) and number of students and teachers all divided by year and cohort | OR and SE | Means and SD not reported, means (with what is probably CIs) only shown in unclear figure where values cannot be read and only for two outcomes. Otherwise only a variety of F-values (conditions, site interaction, pre-test interaction) and sum of two by two treatment groups vs control t-values but only significant values are reported |
| Page numbers and notes | Table 1 and 2 | Text p. 478 | Text p. 458-460 and figure 3 |
| Level of aggregation | Student and teacher | Student | Student |
| Notes | One Head Start centre is control and two centres are treated. Cannot separate centre from treatment effect | Many children obtained standardised scores of zero on the language assessments, resulting in highly skewed distributions, so raw scores were recoded into ordinal categories (0; 1 to 20; >20) and ordinal regression model used. Cannot use in meta-analysis. | Cannot extract data to calculate effect size and SE. Score 5 on selective outcome reporting |
| Used in meta-analysis? | Not used in meta-analysis | Not used in meta-analysis | Not used in meta-analysis |

| Study | McCollum et al., 2013 | Murphy et al. (2017) | Pianta et al. (2017) |
| --- | --- | --- | --- |
| Type of outcome | Continuous | Continuous | Continuous |
| Professional or other? | Teachers | Student | Students |
| Outcome (there may be more than one, record them all) | Learning Environment Checklist, General Classroom Environment, Language, Literacy, and Curriculum, Literacy Activities Rating Scale | Combined average test scores across maths and reading. Test scores are converted into percentiles with a national average of 50 | PPVT Receptive Language, WJ-III Picture Vocabulary, TOPEL Phonological Awareness, TOPEL Print Knowledge |
| Time Point (s) (record the exact time, there may be more than one, record them all) | End of intervention | End of intervention (cohort 1) and 12 months follow up (cohort 2) | One year follow up for course treatment and end of intervention for coaching |
| Source (questionnaire, admin data, other(specify) or unclear) | ELLCO | Key Stage 2 tests. | PPVT = Peabody Picture Vocabulary Test; WJ-III = Woodcock–Johnson III Psychoeducational Battery; TOPEL = Test of Preschool Early Literacy; PLBS = Preschool Learning Behavior Scale. |
| Valid Ns (only applicable for continuous outcome data). Mention treatment and comparison. | Treated: 7, Control: 5 | ITT analysis: 89 treated schools and 92 control schools. Treated/control students in cohort 1: 3157/3284 and cohort 2: 3177/3129. ATE analysis: 69 treated schools and 92 control. Treated/control students in cohort 1 and cohort 2 not reported | For course treatment/control: 60 teachers, 218 students/69 teachers, 261 students. For coaching treatment/control: 35 teachers, 134 students/28 teachers, 107 students. |
| Method of estimation | Means | Regression with covariates | None we can use (unclear what comparison is used in the model) |
| Statistics (risk ratio, odds ratio, standard error, 95 cf, DF, p-value, chi2) | Means, SD (pre and post) | Effect sizes | Means and SD reported pre and post |
| Page numbers and notes | Table 3 p. 33 | Table 5.1 | Table 3 |
| Level of aggregation | Teacher | Student | Student |
| Notes | Score 5 on the Other bias item | Scores 5 on Other bias (the ATE (which is actually a LATE), the ITT scores 4 but cannot be used as it is uncertain what standard deviation is used. Not sure the standard deviations are used for calculating effect sizes but rather standard errors from the model (see p. 15) | Scores 5 on several RoB items |
| Used in meta-analysis? | Not used in meta-analysis | Not used in meta-analysis | Not used in meta-analysis |

| Study | Piasta et al., 2012 | Sandilos et al. (2018) | Scanlon et al., 2008 |
| --- | --- | --- | --- |
| Type of outcome | Continuous | Continuous | Continuous |
| Professional or other? | Students | Teacher | Professionals and students |
| Outcome (there may be more than one, record them all) | Children's linguistic productivity and complexity | Student–teacher interactions, | Teachers: instructional characteristics. Kindergarten: Rhyme Awareness, Beginning Sound Awareness, Alphabet Knowledge, Letter-Sound Knowledge and Spelling components. 1st grade: Spelling and Word Recognition, Basic Reading Skills Cluster |
| Time Point (s) (record the exact time, there may be more than one, record them all) | End of intervention | Between the midterm date of the course and 2 weeks after the last day of class | End of intervention and 1 year follow up |
| Source (questionnaire, admin data, other(specify) or unclear) | Videos: systematic analysis of language transcripts (SALT) | CLASS 3 domains: (i) Emotional Support; (ii) Classroom Organization; (iii) Instructional Support, summaries and all subscales | The Classroom Language Arts Systematic Sampling and Instructional Coding (CLASSIC). The kindergarten version of the Phonological Awareness and Literacy Screening Battery (PALS-K, Invernizzi, Meir, Swank & Juel, 1999–2000). PALS 1–3 (Invernizzi & Meir, 2000–2001) and Woodcock–Johnson III Tests of Achievement (WJIII, Woodcock, McGrew, & Mather, 2001). |
| Valid Ns (only applicable for continuous outcome data). Mention treatment and comparison. | Not reported | Total 239 teachers | Treated: 9 teachers and 145 students; Control: 9 teachers and 124 students |
| Method of estimation | Means | Means | Means |
| Statistics (risk ratio, odds ratio, standard error, 95 cf, DF, p-value, chi2) | Means, SD | Means and SDs | Means, SD (pre (only kindergarten) and post) |
| Page numbers and notes | Table 6, p. 396 | Table 2 | Tables 4-7 |
| Level of aggregation | Unclear, only number of total videos of student and teacher small-group interactions is reported | Teacher | Student and teacher |
| Notes | Score 5 on Other bias. Same trial as reported in Cabell et al., 2011. Cabell et al., 2011 reports on student outcomes and Piasta et al., 2012 reports on students (but transcribed from video only, another outcome than reported in Cabell et al., 2011) | Scores 5 on Incomplete outcome data | Score 5 on Other bias item |
| Used in meta-analysis? | Not used in meta-analysis | Not used in meta-analysis | Not used in meta-analysis |

| Study | Schwanenflugel et al., 2010 | Snow et al. (2014) | Wasik & Hindman, 2011 |
| --- | --- | --- | --- |
| Type of outcome | Continuous | Continuous | Continuous |
| Professional or other? | Students and teachers | Students | Student and teachers |
| Outcome (there may be more than one, record them all) | Alphabet Knowledge, Phonological awareness, Vocabulary knowledge, Early literacy indicators and early decoding skills. Classroom behaviour of teachers | Reading (both Streams) and oral language ability (only Stream A) | Child vocabulary, alphabet knowledge and sound awareness. Classroom quality. |
| Time Point (s) (record the exact time, there may be more than one, record them all) | End of intervention | End of intervention | End of intervention |
| Source (questionnaire, admin data, other(specify) or unclear) | Phonological Awareness Test; PPVT-III: Peabody Picture Vocabulary Test (3rd edition); The kindergarten DIBELS. Expressive Vocabulary. The Revised Early Childhood Environmental Rating Scale (ECERS-R) and the Early Language and Literacy Classroom Observation (ELLCO) | The Reading Progress Test (RPT); the Picture Vocabulary and Syntactic Understanding sub-tests from the Test of Language Development: Primary – Fourth Edition (TOLD-4) although only for Stream A | The Peabody Picture Vocabulary Test—III (PPVT), the Uppercase Alphabet subtest of the Phonological Awareness Literacy Screening (PALS—PreK) and the Rhyme and Beginning Sound Awareness subtests of the PALS—PreK. ELLCO and CLASS. |
| Valid Ns (only applicable for continuous outcome data). Mention treatment and comparison. | Unclear: it is reported that the teachers and students were drawn from 37 classrooms and that there is a total of 350 students participating, number of teachers not reported. | Unclear, see RoB assessment | Treated: 19 teachers and 358 students; Control: 11 teachers and 183 students |
| Method of estimation | Hierarchical regression for student outcomes and means for teacher outcomes | Means | Means |
| Statistics (risk ratio, odds ratio, standard error, 95 cf, DF, p-value, chi2) | Pre-test Adjusted HLM coefficients and SE. Means and SD | Means and SD | Means, SD (pre) and number of students and teachers |
| Page numbers and notes | Tables 3, 5, 6 and 7 | Table 2 | Table 1 |
| Level of aggregation | Class | Students | Student and teacher |
| Notes | One school is control, cannot separate school from treatment effect. Cannot extract data to calculate ES and SE for teacher outcomes | Scores 5 in Other bias | One Head Start centre is control and two centres are treated. Cannot separate centre from treatment effect |
| Used in meta-analysis? | Not used in meta-analysis | Not used in meta-analysis | Not used in meta-analysis |

## Appendix G: Sensitivity figures and funnel plot

### Social and emotional development

Figure G1: Student academic scores, adjusting for clustering ICC=0.05


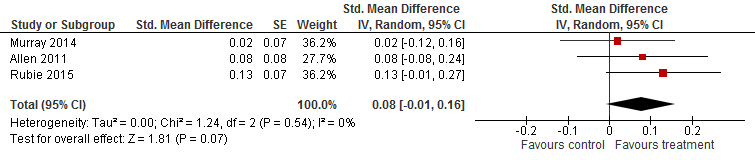


Figure G2: Student academic scores, adjusting for clustering ICC=0.1


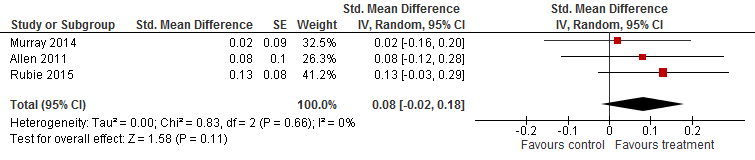


*Figure G3: Student academic scores, adjusting for clustering ICC=0.2*2


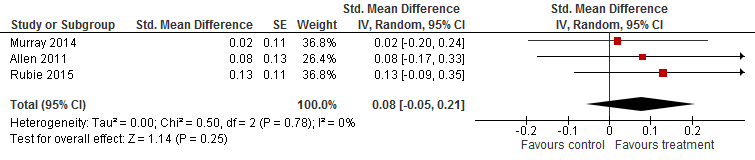


### Language and literacy

*Figure G4: Student academic scores, adjusting for clustering ICC=0.05*


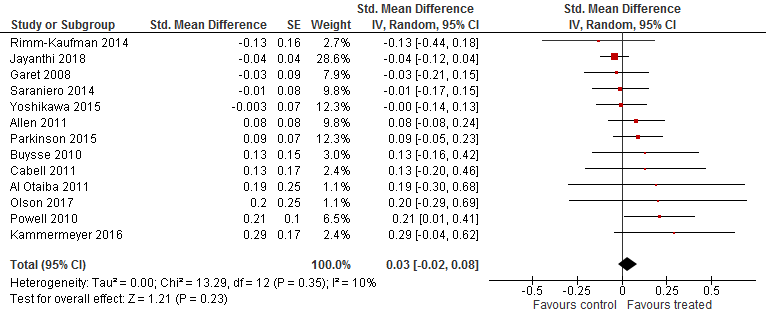


*Figure G5: Student academic scores, adjusting for clustering ICC=0.10*

**
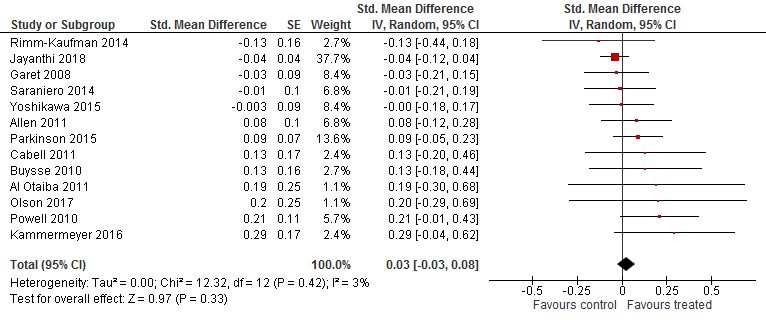
**

*Figure G6: Student academic scores, adjusting for clustering ICC=0.22*


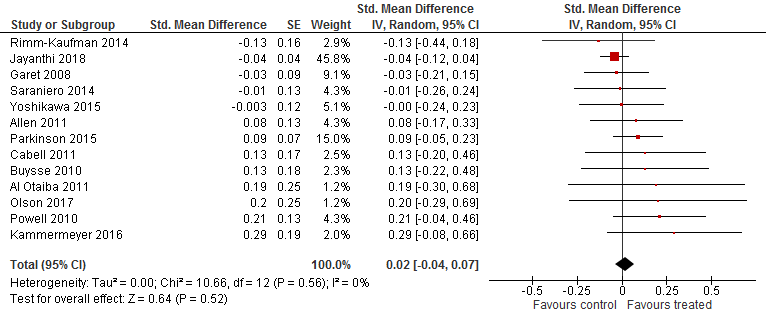


*Figure G7: Summary CLASS, cluster adjusted using ICC: 0.19 for Emotional Support, 0.21 for Classroom Organization, and 0.35 for Instructional Support*


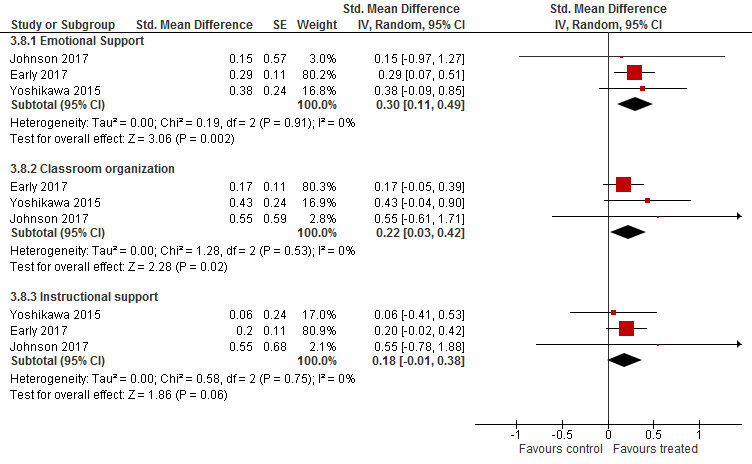


### Funnel plot

*Figure G8: Funnel plot, Language and literacy development, student academic outcomes*


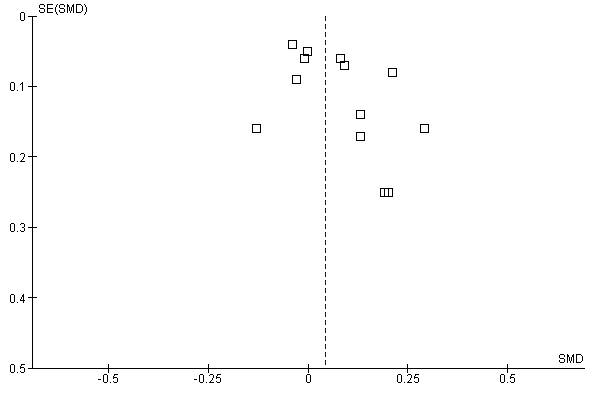

Supplement: Supplementary file 1 — Supporting information [file CL2-15-e1060-s001.docx]
